# New Biocides Based on *N*^4^-Alkylcytidines: Effects on Microorganisms and Application for the Protection of Cultural Heritage Objects of Painting

**DOI:** 10.3390/ijms25053053

**Published:** 2024-03-06

**Authors:** Liudmila A. Alexandrova, Ivan A. Oskolsky, Dmitry A. Makarov, Maxim V. Jasko, Inna L. Karpenko, Olga V. Efremenkova, Byazilya F. Vasilyeva, Darya A. Avdanina, Anna A. Ermolyuk, Elizaveta E. Benko, Stanislav G. Kalinin, Tat’yana V. Kolganova, Maria Ya. Berzina, Irina D. Konstantinova, Alexander O. Chizhov, Sergey N. Kochetkov, Alexander A. Zhgun

**Affiliations:** 1 Engelhardt Institute of Molecular Biology RAS, 32 Vavilov Str., Moscow 119991, Russia; oskosky@yandex.ru (I.A.O.); dmitmakarov_97@mail.ru (D.A.M.); 2003_maxim@mail.ru (M.V.J.); ikarikki@gmail.com (I.L.K.); snk1952@gmail.com (S.N.K.); 2 Gause Institute of New Antibiotics, 11 Bol’shaya Pirogovskaya, Moscow 119021, Russia; ovefr@yandex.ru (O.V.E.); bfvas@yandex.ru (B.F.V.); 3 Research Center of Biotechnology RAS, 33 Leninsky Ave, Moscow 119071, Russia; d.avdanina@gmail.com (D.A.A.); anya_ermolyuk@mail.ru (A.A.E.); elizavetabenko8@gmail.com (E.E.B.); stanislav-kalinin-1990@mail.ru (S.G.K.); moldiag@biengi.ac.ru (T.V.K.); 4 Shemyakin-Ovchinnikov Institute of Bioorganic Chemistry RAS, 16/10 Miklukho-Maklaya str., Moscow 117997, Russia; berzina_maria@mail.ru (M.Y.B.); kid1968@yandex.ru (I.D.K.); 5 Zelinsky Institute of Organic Chemistry RAS 47 Leninsky Ave, Moscow 119991, Russia; chizhov@ioc.ac.ru

**Keywords:** nucleosides, *N*^4^-alkylcytidines, antifungal activity, antibacterial activity, biocides, biodeterioration, protection of cultural heritage

## Abstract

The rapid increase in the antibiotic resistance of microorganisms, capable of causing diseases in humans as destroying cultural heritage sites, is a great challenge for modern science. In this regard, it is necessary to develop fundamentally novel and highly active compounds. In this study, a series of *N*^4^-alkylcytidines, including 5- and 6-methylcytidine derivatives, with extended alkyl substituents, were obtained in order to develop a new generation of antibacterial and antifungal biocides based on nucleoside derivatives. It has been shown that *N*^4^-alkyl 5- or 6-methylcytidines effectively inhibit the growth of molds, isolated from the paintings in the halls of the Ancient Russian Paintings of the State Tretyakov Gallery, Russia, Moscow. The novel compounds showed activity similar to antiseptics commonly used to protect works of art, such as benzalkonium chloride, to which a number of microorganisms have acquired resistance. It was also shown that the activity of *N*^4^-alkylcytidines is comparable to that of some antibiotics used in medicine to fight Gram-positive bacteria, including resistant strains of *Staphylococcus aureus* and *Mycobacterium smegmatis*. *N*^4^-dodecyl-5- and 6-methylcytidines turned out to be the best. This compound seems promising for expanding the palette of antiseptics used in painting, since quite often the destruction of painting materials is caused by joint fungi and bacteria infection.

## 1. Introduction

The discovery of antibiotics was a revolutionary event in the history of mankind, since it became possible to treat a significant number of diseases and save human lives in previously hopeless situations [1,2,3]. However, the widespread introduction of antibiotics into medical practice since the 1950s, at the beginning of the period called the Golden Age of Antibiotics, led to the development of resistance in numerous pathogenic strains against antibiotics of various classes [4,5,6]. This led to the end of the Golden Age of Antibiotics in the early 1970s, and mankind again faced the urgent task of searching for new and improved antimicrobial drugs [7,8,9,10,11,12,13].

Microorganisms can not only be a cause of infectious diseases but also of the destruction of objects of cultural heritage [14,15]. Microorganisms from various systematic groups (especially filamentous fungi) capable of damaging works of art, e.g., tempera painting and oil painting on canvas, have been extensively studied in recent years [16,17,18,19]. There are quite a lot of compounds of various classes used to protect cultural heritage sites, but the number of antiseptics used in painting is extremely limited and has decreased significantly in recent years [20]. Moreover, the compounds used to protect paintings, such as benzalkonium chloride (BAC), do not affect the entire range of microorganisms that destroy painting materials [21]. Moreover, the widespread use of BAC leads to the emergence of resistance in microorganisms against these antiseptics [22].

Therefore, the creation and/or identification of fundamentally new compounds that act on new targets of pathogens and are active against resistant strains of microorganisms is one of the most important problems facing researchers [8,23,24].

Derivatives of natural nucleosides are one of the promising classes of organic compounds, among which prototypes of new drugs are searched. Compounds obtained as a result of the modification of nucleosides are actively studied as drugs to combat viral diseases and bacterial infections, as well as some types of cancer. Currently, nucleoside analogs and derivatives are important elements of antitumor and antiviral therapy and can also be used as antifungal agents [25,26,27,28,29].

**Figure 1:**
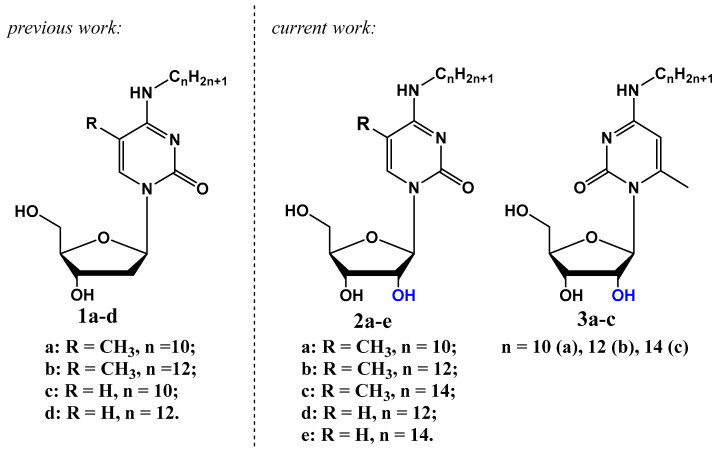
Synthesized *N*^4^-alkylcytidines with antibacterial and antifungal activity.

Previously, we discovered the antibacterial and antifungal activity of a number of modified pyrimidine *N*^4^-alkyl-2′-deoxynucleosides [30,31], containing extended alkyl substituents at the C4 position of the cytosine residue (Figure 1).

These compounds turned out to be active against a number of drug-resistant strains of Gram-positive bacteria, as well as filamentous fungi, while the activity of *N*^4^-derivatives of 5-methyl-2′-deoxycytidine (**1a**,**b**) noticeably exceeded the activity of C4-modified 2′-deoxycytidines (**1c**,**d**) with the same substituent at C4 and was comparable to currently used antibiotics [30]. Ribonucleoside derivatives have not previously been studied in detail, although in the case of decyl derivatives of uridine, we have previously shown that they have better solubility in aqueous media (and, therefore, bioavailability) compared to similar 2′-deoxy analogs and can exhibit pronounced antibacterial activity.

Since the presence of a methyl group fundamentally affects the activity of these compounds, in order to further study the effect of structure on biological activity, we synthesized *N*^4^-cytidine derivatives (**2a**–**e**, **3a**–**c**), both containing a methyl group in the fifth or sixth position of the cytosine residue or not. We hypothesized that nucleoside derivatives containing a substituent at the C6 position of the pyrimidine base may exhibit unusual properties and are also promising objects for research, since they exist in the syn-conformation even in an aqueous environment (unlike most pyrimidine nucleosides) [32,33,34,35]. It was previously shown that 6-modified uridine derivatives can exhibit significant anti-tuberculosis activity [36] and also act as potential antimetabolites; for example, 6-thiocarboxamide-UMP, a structural analog of orotidine-5′-phosphate (OMP), is a potent inhibitor of OMP decarboxylase [37].

## 2. Results

### 2.1. Chemistry

The classical method of modification at the C4 position of pyrimidine nucleosides is the introduction of a good leaving group followed by its replacement with suitable nucleophiles [38,39]. As a rule, derivatives of pyrimidine nucleosides used for this purpose are contained at C4 thio-, thiomethyl-, chloro-, 1,2,4-triazol-1-yl-, 1,2,3,4-tetrazol-1-yl-, arylsulfonyl groups, and a number of others.

To synthesize the target compounds, we decided to use the convenient one-pot method we had previously used for the preparation of *N*^4^-alkyl-2′-deoxynucleosides [30], based on the Diwakar and Reese procedure [40] in its later modification [41], namely, the condensation of nucleosides protected by acetyl groups with 1,2,4-triazole and 2-chlorophenyldichlorophosphate in pyridine followed by a reaction with the corresponding 1-alkylamine and final deblocking.

**Scheme 1:**
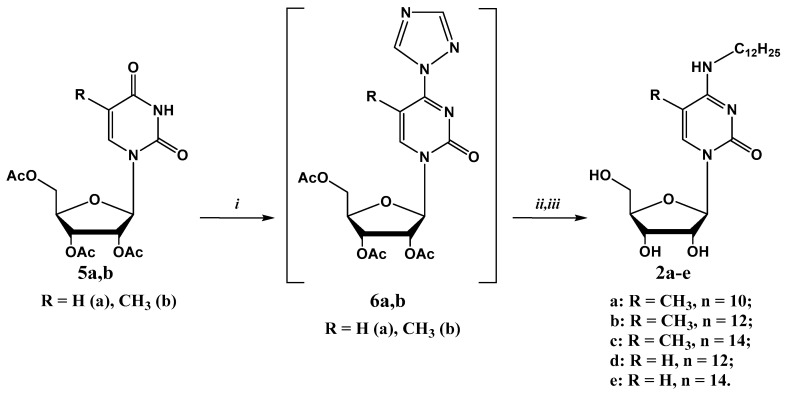
Reagents and conditions: (*i*) 1,2,4-triazole, 2-chlorophenyl dichlorophosphate, pyridine, RT, overnight, (*ii*) C_n_H_2n+1_NH_2_, DIPEA, EtOH, RT, overnight, (*iii*) NH_3_, H_2_O, EtOH, RT, overnight.

It was in this way that we synthesized *N*^4^-alkylamino-5-methylcytidines (**2a**–**c**) and *N*^4^-alkylaminocytidines (**2d**,**e**) in good yields (Scheme 1) starting from 2′,3′,5′-tri-*O*-acetyluridine (**5a**) and 2′,3′,5′-tri-*O*-acetyl-5-methyluridine (**5b**).

**Scheme 2:**
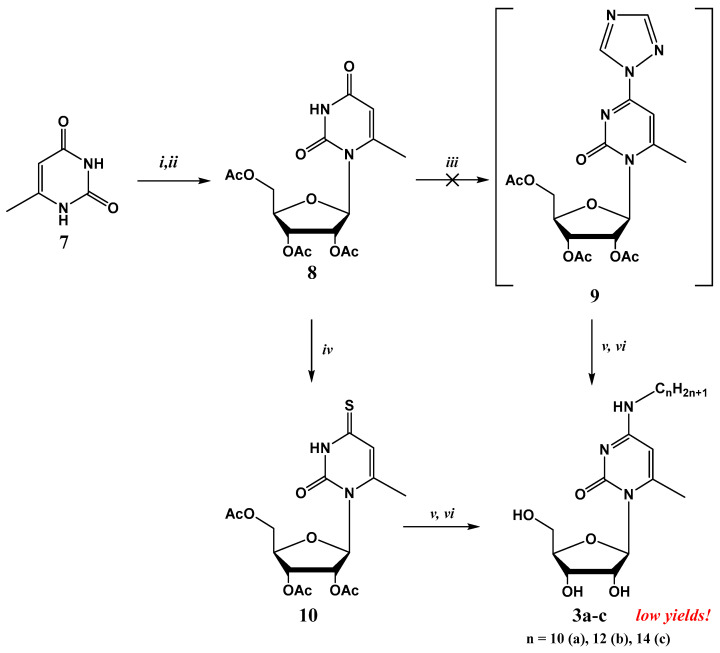
Reagents and conditions: (*i*) bis(trimethylsilyl)-acetamide, DCE or AN, Δ, 15 min; (*ii*) 1′,2′,3′,5′-tetra-*O*-acetylribose, TMSOTf, DCE or AN, Δ, 3 h; (*iii*) 1,2,4-triazole, 2-chlorophenyl dichlorophosphate, pyridine, RT, overnight, (*iv*) Lawesson’s reagent, dioxane, Δ, 3 h; (*v*) C_n_H_2n+1_NH_2_, DIPEA, dioxane (for **9**), EtOH (for **10**), RT, overnight; (*vi*) NH_3_, H_2_O, EtOH, RT, overnight.

The first stage of the synthesis of 6-methyluridine derivatives was the preparation of 2′,3′,5′-tri-*O*-acetyl-6-methyluridine (**7**) by *N*-glycosylation of silylated 6-methyluracil (**6**) 1′,2′,3′,5′-tetra-*O*-acetylribose according to the Vorbruggen method, using trimethylsilyl trifluoromethanesulfonate as a catalyst [36,42] (Scheme 2).

Both dichloroethane and acetonitrile were tried as solvents for these reactions. When *N*-glycosylation is run in acetonitrile, the yield of compound (**8**) is higher than when using dichloroethane. Moreover, in dichloroethane, there is a significant formation of the *N*^3^-isomer of protected 6-methyluridine in an almost equal ratio with the *N*^1^-isomer, while in acetonitrile the formation of the *N*^3^-isomer was practically not observed. As a result, 2′,3′,5′-tri-*O*-acetyl-6-methyluridine (**8**) was synthesized with a 67% yield.

The second stage of the synthesis was the replacement of oxygen at the C4 position with a good leaving group. First of all, we tried the same approach that we used earlier [30], namely, the replacement of the oxygen atom with a 1,2,4-triazolyl group in the presence of 2-chlorophenyldichlorophosphate in pyridine (Scheme 2), but it was not possible to obtain a triazolyl derivative. Apparently, the 6-methyl group in the meta position with respect to the 4-carbonyl group leads to a reduced reactivity of the latter.

**Scheme 3:**
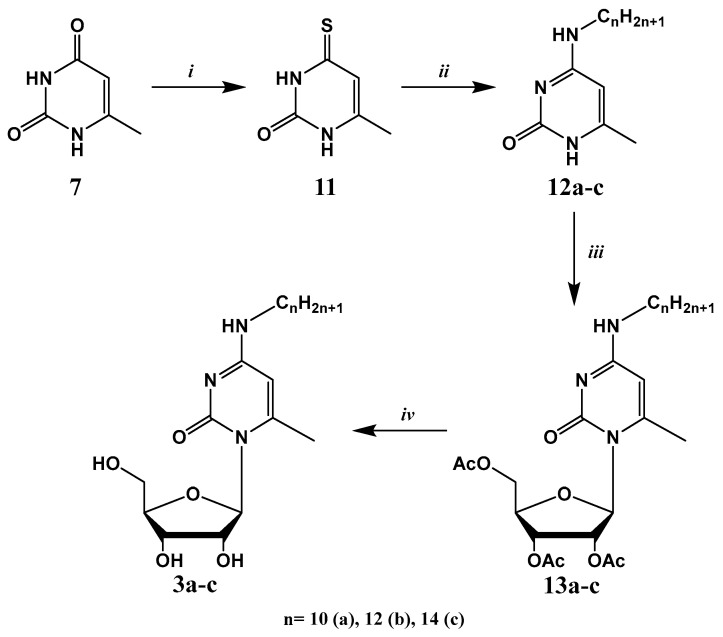
Reagents and conditions: (*i*) Lawesson’s reagent, dioxane, pyridine, Δ, 3 h; (*ii*) C_n_H_2n+1_NH_2_, 7-methylquinoline, ethylene glycol, Δ, 2-3 h; (*iii*) (1) bis(trimethylsilyl)acetamide, AN, RT, 15 min; (2) 1′,2′,3′,5′-tetra-O-acetylribose, TMSOTf, AN, Δ, 3 h; (*iv*) NH_3_, H_2_O, EtOH, RT, overnight.

Next, we synthesized 2′,3′,5′-tri-*O*-acetyl-6-methyl-4-thiouridine (**10**) using Lawesson’s reagent [43] by boiling compound (**8**) in dioxane under argon atmosphere for 4 h (Scheme 2) with a yield of 50%; however, attempts to replace the thio group with the corresponding alkylamine led to compounds (**3a**–**c**) with unsatisfactory yields. *N*^4^-Alkyl derivatives of 6-methylcytidine (**3a**–**c**) were synthesized starting from *N*^4^-alkyl-6-methylcytosines (**12a**–**c**) in accordance with Scheme 3. The first stage of the synthesis was the preparation of 6-methyl-4-thiouracil (**11**) by replacing the oxygen atom in the C4 position of 6-methyluracil (**7**) with a sulfur atom using Lawesson’s reagent when heated in a mixture of pyridine and dioxane (Scheme 3) for 4 h at boiling. Next, *N*^4^-alkyl-6-methylcytosines (**13**) were synthesized by analogy with the known procedure for the preparation of 5-aminouracils [44,45] (Scheme 3). Base (**11**) and the corresponding 1-alkylamine were refluxed in ethylene glycol in the presence of 7-methylquinoline for 1 h. The product was isolated by precipitating it from the reaction mixture with water and then washing the resulting precipitate with ethyl acetate.

The next stage of the synthesis is the *N*-glycosylation of the resulting *N*^4^-alkyl-6-methylcytosines (**12a**–**c**) with 1′,2′,3′,5′-tetra-*O*-acetylribose (Scheme 3) according to the Vorbruggen method [42]. The condensation of silylated *N*^4^-alkyl-6-methylcytosines (**12a**–**c**) with 1′,2′,3′,5′-tetra-*O*-acetylribose in acetonitrile was carried out catalyzed by trimethylsilyl trifluoromethane sulfonate. When heated at a temperature of 82 °C, the reaction took 3.5 h and was stopped after the complete consumption of sugar; however, better yields (65–80%) were achieved when the reaction was carried out at 37 °C for several days. To obtain the target nucleoside derivatives (**3a**–**c**), protected *N*^4^-alkyl-6-methylcytidines (**13a**–**c**) were deblocked with aqueous 25% ammonia in ethanol (Scheme 3) for 24 h at 20 °C and isolated by column chromatography.

The purity and structure of the target compounds were confirmed by ^1^H- and ^13^C-NMR spectroscopy and high-resolution mass spectrometry.

### 2.2. Enzymology

Due to the complexity of the synthesis of 6-methylcytidine derivatives, it seemed appropriate to us to study the possibility of the enzymatic synthesis of nucleosides using nucleoside phosphorylases (NPs). The use of (NPs) in the enzymatic synthesis of nucleosides is well known and has recently received considerable attention. NP-catalyzed nucleoside transglycosylation uses a readily available nucleoside such as natural uridine or thymidine as a starting compound [46,47].

It was shown previously that a number of uracil derivatives with 6-CH_2_OH and 6-CH_2_F substituents are substrates of *Escherichia coli* pyrimidine nucleoside phosphorylase [33]; however, 6-methyluracil was not a substrate of uridine phosphorylase from *E. coli* [46]. 6-Methyluridine is slowly converted into 6-methyluracil, and the substrate binding is significantly weaker compared to the 5-methyl derivative [46].

**Scheme 4:**
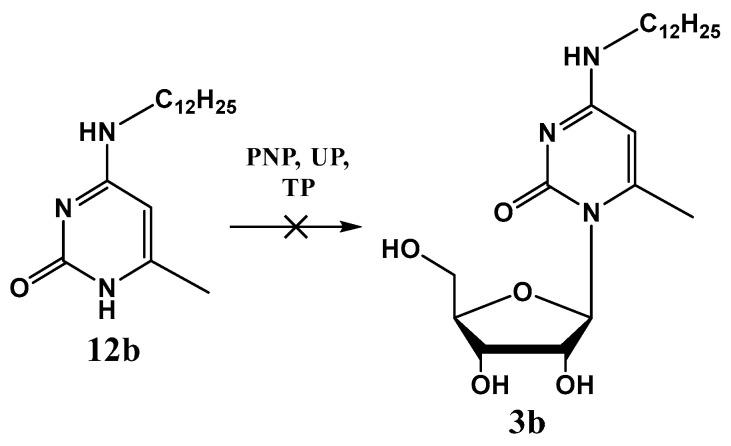
Enzymes: purine nucleoside phosphorylase (PNP), uridine phosphorylase (UP), thymidine phosphorylase (TP).

We studied the enzymatic glycosylation of *N*^4^-dodecyl-6-methylcytosine (**12b**), catalyzed by purine nucleoside phosphorylase (PNP), uridine phosphorylase (UP), and thymidine phosphorylase (TP) (Scheme 4).

Unfortunately, *N*^4^-dodecyl-6-methylcytosine (**12b**) was not a substrate for any of the enzymes used. (See Supplementary Materials Figure S1).

### 2.3. Cytotoxicity

The cytotoxicity of the synthesized compounds (CD_50_) was estimated by an MTT assay [48] in *HeLa* and *Vero* E6 cell lines. The compounds **2a**–**e** and **3a**–**c** demonstrated cytotoxic activity at concentrations of 25–60 μM.

### 2.4. In Vitro Study of Antibacterial Activity of the Obtained Compounds

#### In Vitro Study of Antibacterial Activity of the Obtained Compounds

The antibacterial effect of the obtained compounds was studied by their ability to inhibit in vitro the growth of Gram-positive and Gram-negative bacterial strains, listed in the Methods. Antimicrobial activity was observed only against Gram-positive bacteria, including methicillin-resistant staphylococcus and two drug-resistant strains of mycobacteria (Table S1, Supplementary Materials). A previously developed method was used [49,50].

**Figure 2:**
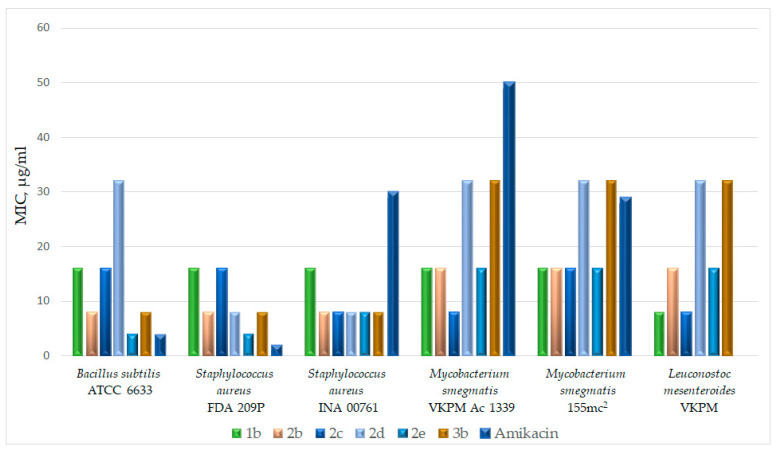
The inhibitory effect (MIC, μg/mL) of the most active compounds and the antibiotic amikacin against a number of Gram-positive bacteria.

Figure 2 schematically shows the inhibitory effect of the most active compounds and the antibiotic amikacin against a number of Gram-positive bacteria.

Among the *N*^4^-alkylcytidine derivatives studied, *N*^4^-decylcytidine **2e** has the lowest MIC value in the range from 4 to 16 μg/mL. The MIC of both *N*^4^-dodecyl derivatives **2b** and **3b** against five Gram-positive bacteria is 8 μg/mL, with the exception of the MIC of **3b** against *M. luteus* NCTC 8340 (32 μg/mL); the MIC against the two strains of *Myc. smegmatis* is slightly higher and amounts to 16 and 32 μg/mL, respectively. The 2′-deoxy derivative **1b** showed a uniform MIC value against all seven Gram-positive bacteria. The antibacterial effect of compound **2e** and *N*^4^-dodecyl derivatives **1b**, **2b**, and **3b** is comparable to the effect of a number of antibiotics used in medical practice. Compounds **3a** and **3c** with decyl and tetradecyl substituents and cytosine derivatives **12a**–**c** did not demonstrate antibacterial activity in the studied concentration range.

We have shown that the effects of *N*^4^-alkyl derivatives of 5- and 6-methylcytidine are different. While *N*^4^-dodecyl-6-methylcytidine (**3b**) exhibits bactericidal activity with an MIC value of 8 μg/mL against Gram-positive bacteria and an MIC of 32 μg/mL against two strains of mycobacteria, *Myc. smegmatis* VKPM Ac 1339 and *Myc. smegmatis* mc^2^ 155, both *N*^4^-dodecyl-5-methylcytidine (**2b**) and its 2′-deoxy analog (**1b**) also have an MIC of 8–16 μg/mL against all of the bacteria listed above; however, against two strains of mycobacteria, the activity exhibited is bacteriostatic in nature since, despite the absence of mycobacterial growth for 5 days, upon subsequent reseeding of the bacteria on fresh medium without these compounds, growth resumes.

### 2.5. In Vitro Study of the Antifungal Activity of the Obtained Compounds

Previously, we have shown that 4-modified derivatives of 5-methyl-2′-deoxycytidine with extended alkyl substituents exhibit high inhibitory activity against filamentous fungi, which cause the biodegradation of organic materials used in tempera painting of the 15th–16th centuries [30,31]. The antifungal activity of the obtained nucleoside derivatives, as compared with the previously obtained *N*^4^-dodecyl-5-methyl-2′-deoxycytidine **1b** [30,31], was studied against 12 filamentous fungi, belonging to the types *Ascomycota* and *Mucoromycota*, isolated in the halls of the Ancient Russian Paintings of the State Tretyakov Gallery and capable of the biodegradation of ancient Russian icons [15,20,51].

Previous experiments showed that, among *N*^4^-alkyl-5-methyl-2′-deoxycytidines in the C8-C10-C12 alkyl series, the dodecyl derivative has the greatest antifungal activity (**1b**) [30]. Then we demonstrated that this activity could be somewhat enhanced by replacing the hydroxyl group at the 3′ position of deoxyribose with an amino group or methyl-, ethyl-, or dimethyl-amino groups [31]. However, a number of other modifications of the side radicals of the molecule lead to an almost complete loss of antimycotic activity [52]. In particular, we have identified the dependence of the antifungal activity of alkyl derivatives of pyrimidine nucleosides on the position of the long alkyl substituent in the nitrogenous base residue [53]. It appeared that changing the position from N-4 to C-5 to introduce an extended alkyl substituent, leads to a complete loss of antifungal activity.

#### 2.5.1. The Role of the Hydroxyl Group in the 2′-Position of Pentose

**Figure 3:**
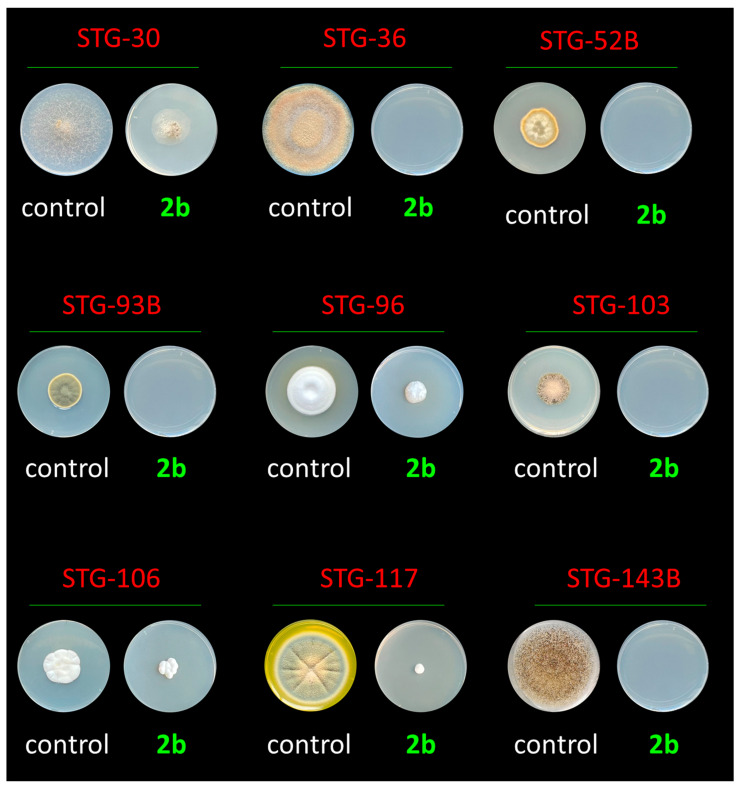
The phenotype of fungi strains on CDA medium, supplemented with the 200 µM addition of 2b, or without additives (control). Petri dishes were captured in 5 days (for STG-30 and STG-143B) or in 12 days (all other strains) after inoculation.

**Figure 4:**
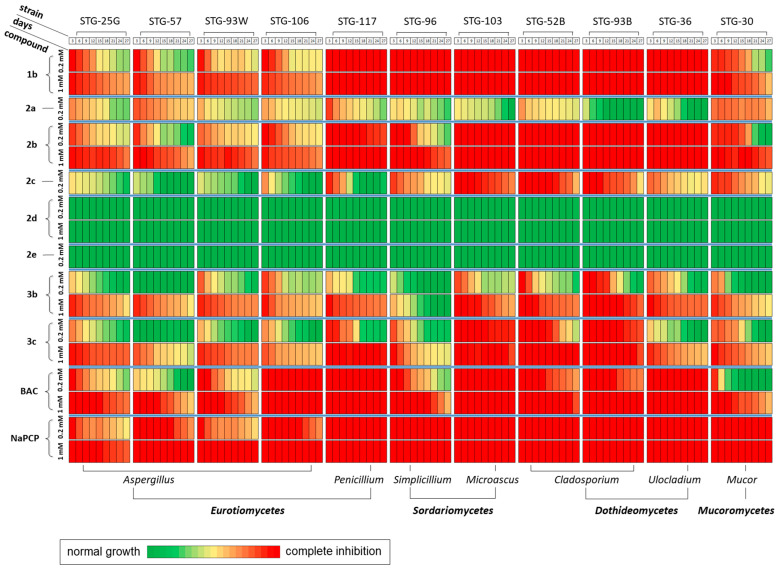
The dynamics of the relative growth inhibition (%) for STG strains on the CDA medium with the synthesized compounds, BAC, and NaPCP (all tested at concentrations of 200 μM and 1000 μM). Data were collected within 3–27 days after inoculation every 3 days.

In this work, we showed that the addition of an OH- group to the 2′-position of deoxyribose does not affect the antimycotic activity. Thus, ribo-derivative **2b** (*N*^4^-dodecyl-5-methyl-cytidine) has similar activity as its 2′-deoxy analog **1b** (Figure 3 and Figure 4). It turned out that the addition of **1b** and **2b** at both tested concentrations, 200 and 1000 μM, led to almost the same inhibition dynamics profile in all tested strains (Figure 4). The exceptions were strain STG-25G, against which **2b** was more active after 15 days, and STG-96, against which **1b** was more active after the same period of time. However, the differences were insignificant, which indicates a fundamentally similar effect of **1b** and **2b** on test cultures of fungi-destructors.

#### 2.5.2. The Role of the Methyl Group in the Fifth or Sixth Position of Cytidine Derivatives

The results obtained, i.e., that the replacement of 2′-deoxyribose with ribose in the sugar residue of the nucleoside does not significantly affect the antimycotic activity, allowed us to study the role of the methyl group and the length of the alkyl radical in the cytosine residue also in ribo-derivatives of cytidines. As a result, we demonstrated for the first time that the removal of the methyl group at the fifth position leads to a complete decrease in the activity of compounds of this class. Moreover, a complete loss of activity in the tested concentration range (200–1000 μM) was observed in both **2d** (*N*^4^-dodecylcytidine) and **2e** (*N*^4^-tetradecylcytidine) (Figure 4). The replacing of the methyl group from the fifth to the sixth position resulted in a significant loss of activity; compounds **3b** (*N*^4^-dodecyl-6-methylcytidine) and **3c** (*N*^4^-tetradecyl-6-methylcytidine) turned out to be 3–8 times less active than **2b** (*N*^4^-dodecyl-5-methylcytidine).

#### 2.5.3. The Role of the Size of Alkyl Group in the N^4^ Position of Cytidine Derivatives

**Figure 5:**
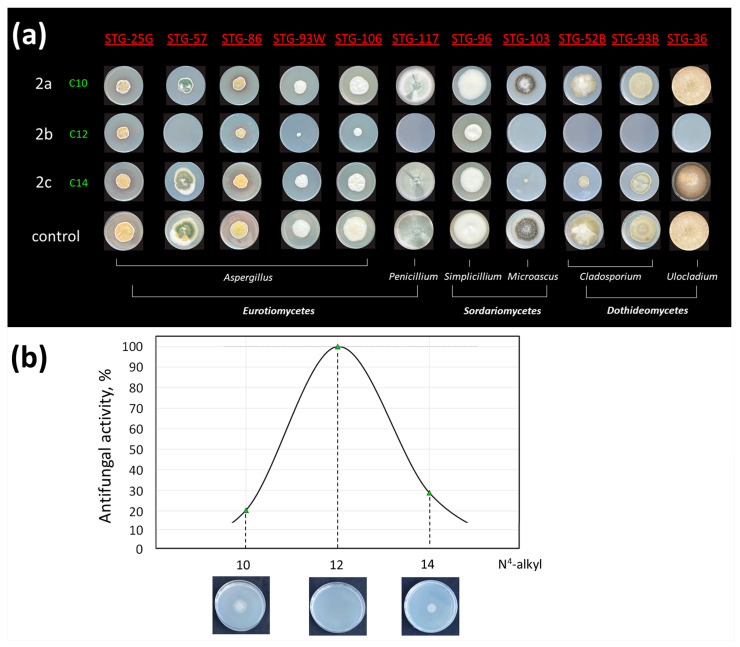
The effect of the size of the alkyl group at *N*^4^ on the fungicidal activity of compounds: (**a**) the phenotype of fungi strains on CDA medium, supplemented with the 200 µM addition of **2a**–**c** or without additives (control); Petri dishes were captured in 12 days after inoculation; (**b**) antifungal activity related to the activity of 2**b**.

For *N*^4^-alkyl-5-methylcytidines in the series C10-C12-C14, a clear bell-shaped dependence of activity on the size of the alkyl substituent at *N*^4^ was observed (Figure 5). For all strains, the most active compound was the *N*^4^-dodecyl derivative. The activity dropped significantly both when the alkyl radical increased to C14 (about three times) and when it decreased to C10 (about five times), Figure 5.

In the series *N*^4^-alkyl-6-methylcytidines C12–C14, cross-activity was observed; for 25% of the studied fungal strains, the C12 derivative **3b** was more active, for 42%, the C14 derivative **3c** was more active, and in 33% of cases, both compounds showed similar activity. It is possible that this cross-effect is due to differences in resistance mechanisms in different fungal strains, on which C12 and C14 derivatives of 6-methylcytidines have different effects. This cross-activity suggests that the optimal broad-spectrum antiseptic based on 6-methylcytidines derivatives should presumably consist of a cocktail of *N*^4^-dodecyl- and *N*^4^-tetradecylcytidines. However, the movement of the methyl group to the fifth position of cytidine makes the compound not only more active but also universal in the size of the alkyl radical: only C12 derivatives show the best activity against all test cultures; making a cocktail with C10 or C14 derivatives to expand the spectrum of action does not seem promising.

## 3. Discussion

For further research into microbial inhibitors, rational methods for the synthesis of *N*^4^-alkyl derivatives of cytidines, 5- and 6-methylcytidines, were developed. In contrast to the simple one-pot synthesis of *N*^4^-alkyl 5-methylcytidines (**2a**–**c**) and cytidines (**2d**,**e**), *N*^4^-alkyl-6-methylcydine (**3a**–**c**) was obtained only by N-glycosylation of the corresponding *N*^4^-alkyl-6-methylcytosines (**12a**–**c**).

It was shown that *N*^4^-tetradecyl-6-methylcytosines (**12c**) are not substrates of the three nucleoside phosphorylases usually used for the enzymatic synthesis of nucleosides.

The antibacterial effect of the obtained compounds was studied by their ability to inhibit in vitro the growth of a number of microorganisms: seven strains of Gram-positive bacteria, including drug-resistant strains of *Myc. smegmatis* and *S. aureus*, and two strains of Gram-negative bacteria. *N*^4^-Alkyl-6-methylcytosines (**12a**–**c**) did not demonstrate inhibitory properties. The synthesized *N*^4^-alkyl derivatives of nucleosides (**2a**–**e**) effectively inhibited the growth of Gram-positive bacteria but did not affect Gram-negative bacteria. The antibacterial effect of *N*^4^-decylcytidine **2e** and *N*^4^-dodecyl derivatives, both 2′-deoxy-5-methylcytidine **1b**, and 5- or 6-methylcytidine (**2b** or **3b**) is comparable to the effect of a number of antibiotics used in medical practice. Compounds **3a**, **3c** with decyl and tetradecyl substituents, and cytosine derivatives **12a**–**c** did not demonstrate antibacterial activity in the studied concentration range.

As expected, the effects of *N*^4^-alkyl derivatives of 5- and 6-methylcytidine are different. While *N*^4^-dodecyl-6-methylcytidine (**3b**) exhibits bactericidal activity against two strains of mycobacteria, *Myc. smegmatis* VKPM Ac 1339 and *Myc. smegmatis* mc^2^ 155, both *N*^4^-dodecyl-5-methylcytidine (**2b**) and its 2′-deoxy analog (**1b**) are also active against all of the bacteria listed above; however, against two strains of mycobacteria, the activity exhibited is bacteriostatic in nature.

During antifungal experiments, several patterns were established. (i) The presence of a methyl group at the fifth position of *N*^4^-alkyl-cytidines is critical for antifungal activity (Figure 4). The removal of the methyl group or its replacement to the sixth position results in either a complete or significant loss of activity (3–8 times), respectively. (ii) The presence of a hydroxyl group in the 2′ position does not significantly affect the activity of compounds of this class. (iii) Among the group of *N*^4^-alkyl-5-methylcytidines, which turned out to be the most active compounds against the tested molds, in the C10-C12-C14 series, the *N*^4^-dodecyl derivative works best. Reducing or increasing the length of the alkyl substituent leads to a loss of activity by 3–5 times. Less active *N*^4^-alkyl-6-methylcytidines have cross-activity, some fungal strains are more sensitive to C12, others to C14, and there are also strains on which both compounds act equally.

**Figure 6:**
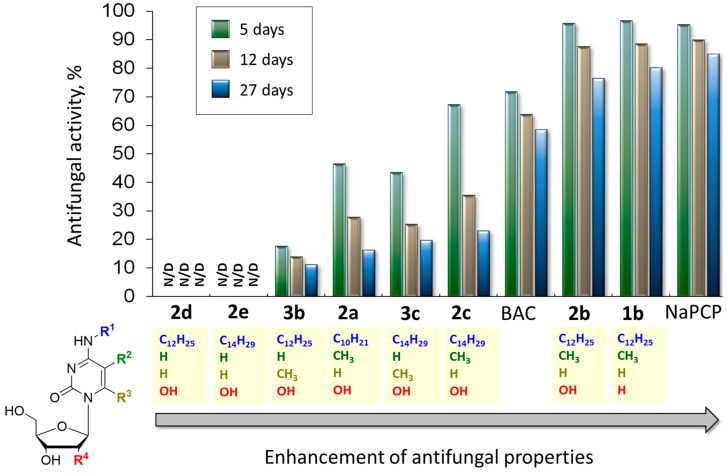
The relative antifungal activity of 200 μM synthetized compounds, BAC, and NaPCP at 5, 12, and 27 days after inoculation. Grown as the ratio of the size of the colonies of all fungal strains on the medium supplemented with compound in relation to the size of the colonies in the control. The compounds are arranged in order of increasing antifungal activity on day 27.

To evaluate the possible application of alkylnucleosides for the protection of paintings, the activity of standard antiseptics used for biodamaged paintings, such as BAC and NaPCP, was also studied. It turned out that NaPCP exhibits the best activity among the studied drugs, but currently, it is in most cases withdrawn from restoration practice due to its high toxicity to humans [54] (Figure 6).

Antiseptic BAC, actively used to protect paintings, works generally worse than **1b** and **2b** and slightly better than **2a**,**c** and **3b**,**c**. It is also necessary to take into account that BAC is a cocktail of quaternary amines, in which one of the substituents is an alkyl with a variable value from C8 to C18. In this case, a wide spectrum of action is achieved due to the fact that different groups of microorganisms are most sensitive to compounds with different lengths of alkyl radical. Thus, the demonstrated resistance of a number of strains to it, such as STG-25G, STG-30, STG-52B, STG-57, STG-93W, and STG-96, can no longer be controlled by varying the size of this alkyl radical. On the other hand, various modifications in alkyl nucleosides can lead to cross-activity, for example, replacing the hydroxyl group in the 3′-position of a sugar residue with an amino group leads to additional activity against *Aspergillus* [31]. From this point of view, cocktails based on alkyl nucleosides may potentially have an even wider spectrum of activity than BAC.

Since some of the studied compounds exhibit high activity (at the level of applied antiseptics) against a specific group of microorganisms that destroy paintings, these alkylcytidines can potentially be used as targeted antiseptics. At the next stage of research, it will be necessary to study both the antimicrobial activity of these new compounds in the composition of painting materials and the level of impact on their spectral and surface properties.

## 4. Materials and Methods

### 4.1. General Information

Commercial reagents from Fluka (Buchs, Switzerland), Sigma-Aldrich (St. Louis, MO, USA), and Acros Organics (Geel, Belgium) were used in this work. The commercial antiseptics used to protect paintings are as follows: Benzalkonium chloride (BAC, commercial name Katamin AB) from Neochemax (Domodedovo, Russia); sodium pentachlorophenolate (NaPCP) from IndiaMART (Noida, India).

Solvents were purified using standard methods. Column chromatography was performed using Kieselgel 60 (40–63 μm) silica gel (Merck, Darmstadt, Germany). ^1^H and ^13^C NMR spectra were recorded with a Bruker (Bremen, Germany) AM300 at ambient temperature in DMSO-*d*_6_ and CDCl_3_ solutions. Chemical shift values are given in δ scale relative to Me_4_Si. The *J* values are given in hertz. UV spectra were recorded on a Perkin Elmer lambda 25 spectrophotometer (Perkin Elmer, Shelton, CT, USA) in methanol. HR-ESI-MS were measured on a Bruker Daltonics micrOTOF II instrument (Bruker Daltonik GmbH, Bremen, Germany). All reactions were monitored with thin-layer chromatography (TLC) and carried out with Merck (Darmstadt, Germany) precoated plates DC-AlufolienKieselgel60 F254.

*N*^4^-Dodecyl-5-methyl-2′-deoxycytidine **1b** was obtained using the method found in [30].

#### General Method for the Synthesis of Compounds **2a**–**e**

2-Chlorophenyl dichlorophosphate (0.255 g, 0.173 mL, 1.05 mmol) was added to a solution of acetyl-protected uridine (**5a**) or 5-methyluridine (**5b**) (0.5 mmol) and 1,2,4-triazole (0.2 g, 3 mmol) in anhydrous pyridine, cooled to 0 °C. The mixture was stirred for 20 h at room temperature and then evaporated. The residue was partitioned between chloroform and 0.5 M sodium bicarbonate; the chloroform layer was washed with water, dried over Na_2_SO_4_, evaporated, and dissolved in anhydrous dioxane (3 mL). The corresponding 1-alkylamine (0.6 mmol) and diisopropylethylamine (75 mg, 0.1 mL, 0.6 mmol) were added to a solution and cooled to 0 °C. The mixture was stirred for 20 h at room temperature, then 3 mL of conc. aq. ammonia solution was added, and the mixture was stirred for 40 h at room temperature and then evaporated; the compounds were purified on a column of silica gel (2 × 15 cm) in chloroform or ethyl acetate eluted with a gradient of ethanol in chloroform (0–15%) or in ethyl acetate (0–10%), respectively. The target fractions were evaporated in a vacuum to give the expected compound yields as colorless amorphous mass with 60–85% yields.

*N*^4^-Decyl-5-methylcytidine (**2a**). Prepared according to the general procedure from **5b** (0.192 mg) and 1-decylamine (0.079 mg). Yield 0.150 g (76%). UV: λ_max_ = 272 nm. ^1^H NMR (300 MHz, DMSO-*d_6_*) δ 7.64 (q, *J* = 1.2 Hz, 1H, 6-H), 7.12 (t, *J* = 5.6 Hz, 1H, 4-NH), 5.77 (d, *J* = 3.8 Hz, 1H, 1′-H), 5.23 (d, *J* = 4.7 Hz, 1H, 2′-OH), 5.06 (t, *J* = 5.2 Hz, 1H, 5′-OH), 4.94 (d, *J* = 5.0 Hz, 1H, 3′-OH), 3.91–4.02 (m, 2H, 2′-H + 3′-H), 3.81 (dt, *J* = 3.6, 3.5 Hz, 1H, 4′-H), 3.67 (ddd, *J* = 12.0, 5.0, 3.2 Hz, 1H, 5′-Ha), 3.55 (ddd, *J* = 12.1, 5.3, 3.4 Hz, 1H, 5′-Hb), 3.33–3.23 (m, 2H, NH-CH_2_-), 1.84 (d, *J* = 1.0 Hz, 3H, 5-CH_3_), 1.46–1.57 (m, 2H, NH-CH_2_-CH_2_-), 1.19–1.37 (m, 14H, NH-(CH_2_)_2_-(CH_2_)_7_-), 0.85 (t, *J* = 6.9 Hz, 3H, -CH_2_-CH_3_). ^13^C NMR (75 MHz, DMSO-*d_6_*) δ 162.66 (4-C), 155.44 (2-C), 137.73 (6-C), 101.54 (5-C), 88.99 (1′-C), 84.08 (4′-C), 73.78 (2′-C), 69.48 (3′-C), 60.72 (5′-C), 40.24 (NH-CH_2_), 31.29, 28.96, 28.66, 26.49, 22.08, 13.92, 13.06 (NH-CH_2_-(CH_2_)_8_-CH_3_ + 5-CH_3_). HRMS (ESI) of C_20_H_35_N_3_O_5_, *m*/*z*: calcd [M + H]^+^ 398.2649, found: 398.2645.

*N*^4^-Dodecyl-5-methylcytidine (**2b**). Prepared according to the general procedure from **5b** (0.192 g) and 1-dodecylamine (0.111 g). Yield 0.165 g (78%). UV: λ_max_ = 272 nm. ^1^H NMR (300 MHz, DMSO-*d_6_*) δ 7.63 (q, *J* = 1.2 Hz, 1H, 6-H), 7.11 (t, *J* = 5.6 Hz, 1H, 4-NH), 5.77 (d, *J* = 3.8 Hz, 1H, 1′-H), 5.21 (d, *J* = 4.7 Hz, 1H, 2′-OH), 5.05 (t, *J* = 5.2 Hz, 1H, 5′-OH), 4.93 (d, *J* = 4.9 Hz, 1H, 3′-OH), 3.90–4.00 (m, 2H, 2′-H + 3′-H), 3.81 (ddd, *J* = 3.5, 3.4, 3.2 Hz, 1H, 4′-H), 3.66 (ddd, *J* = 12.0, 5.2, 3.2 Hz, 1H, 5′-Ha), 3.54 (ddd, *J* = 12.0, 5.4, 3.5 Hz, 1H, 5′-Hb), 3.24–3.32 (m, 2H, NH-CH_2_-), 1.83 (d, *J* = 1.0 Hz, 3H, 5-CH_3_), 1.43–1.57 (m, 2H, NH-CH_2_-CH_2_-), 1.20–1.32 (m, 18H, NH-(CH_2_)_2_-(CH_2_)_9_-), 0.85 (t, *J* = 6.9 Hz, 3H, -CH_2_-CH_3_). ^13^C NMR (75 MHz, DMSO-*d_6_*) δ 162.65 (4-C), 155.39 (2-C), 137.73 (6-C), 101.48 (5-C), 88.96 (1′-C), 84.07 (4′-C), 73.74 (2′-C), 69.48 (3′-C), 60.73 (5′-C), 40.22 (NH-CH_2_), 31.28, 28.97, 28.65, 26.49, 22.08, 13.92, 13.06 (NH-CH_2_-(CH_2_)_10_-CH_3_ + 5-CH_3_). HRMS (ESI) of C_22_H_39_N_3_O_5_, *m*/*z*: calcd [M + H]^+^ 426.2962, found: 426.2951.

*N*^4^-Tetradecyl-5-methylcytidine (**2c**). Prepared according to the general procedure from **5b** (0.192 g) and 1-tetradecylamine (0.128 g). Yield 0.177 g (78%). UV: λ_max_ = 272 nm. ^1^H NMR (300 MHz, DMSO-*d_6_*) δ 7.63 (q, *J* = 1.2 Hz, 1H, 6-H), 7.11 (t, *J* = 5.7 Hz, 1H, 4-NH), 5.77 (d, *J* = 3.8 Hz, 1H, 1′-H), 5.21 (d, *J* = 5.3 Hz, 1H, 2′-OH), 5.05 (t, *J* = 5.2 Hz, 1H, 5′-OH), 4.93 (d, *J* = 5.16 Hz, 1H, 3′-OH), 3.91–4.00 (m, 2H, 2′-H + 3′-H), 3.81 (dt, *J* = 3.7, 3.5, 3.5 Hz, 1H, 4′-H), 3.67 (ddd, *J* = 12.0, 5.1, 3.2 Hz, 1H, 5′-Ha), 3.54 (ddd, *J* = 12.0, 5.3, 3.5 Hz, 1H, 5′-Hb), 3.24–3.32 (m, 2H, NH-CH_2_-), 1.84 (d, *J* = 1.0 Hz, 3H, 5-CH_3_), 1.44–1.59 (m, 2H, NH-CH_2_-CH_2_-), 1.20–1.33 (m, 22H, NH-(CH_2_)_2_-(CH_2_)_11_-), 0.86 (t, *J* = 6.9 Hz, 3H, -CH_2_-CH_3_). ^13^C NMR (75 MHz, DMSO-*d_6_*) δ 162.65 (4-C), 155.38 (2-C), 137.72 (6-C), 101.47 (5-C), 88.97 (1′-C), 84.06 (4′-C), 73.74 (2′-C), 69.46 (3′-C), 60.71 (5′-C), 40.21 (NH-CH_2_), 31.28, 29.02, 28.65, 26.50, 22.07, 13.90, 13.04 (NH-CH_2_-(CH_2_)_12_-CH_3_ + 5-CH_3_). HRMS (ESI) of C_24_H_43_N_3_O_5_, *m*/*z*: calcd [M + H]^+^ 454.3275, found: 454.3281.

*N*^4^-Dodecylcytidine (**2d**). Prepared according to the general procedure from **5a** (0.185 g) and 1-dodecylamine (0.111 g). Yield 0.138 g (67%). UV: λ_max_ = 272 nm. ^1^H NMR (300 MHz, DMSO-*d_6_*) δ 7.63 (q, *J* = 1.2 Hz, 1H, 6-H), 7.11 (t, *J* = 5.7 Hz, 1H, 4-NH), 5.77 (d, *J* = 3.8 Hz, 1H, 1′-H), 5.21 (d, *J* = 5.3 Hz, 1H, 2′-OH), 5.05 (t, *J* = 5.2 Hz, 1H, 5′-OH), 4.93 (d, *J* = 5.2 Hz, 1H, 3′-OH), 3.91–4.00 (m, 2H, 2′-H + 3′-H), 3.81 (dt, *J* = 3.7, 3.5, 3.5 Hz, 1H, 4′-H), 3.67 (ddd, *J* = 12.0, 5.1, 3.2 Hz, 1H, 5′-Ha), 3.54 (ddd, *J* = 12.0, 5.3, 3.5 Hz, 1H, 5′-Hb), 3.24–3.32 (m, 2H, NH-CH_2_-), 1.84 (d, *J* = 1.0 Hz, 3H, 5-CH_3_), 1.44–1.59 (m, 2H, NH-CH_2_-CH_2_-), 1.20–1.33 (m, 22H, NH-(CH_2_)_2_-(CH_2_)_11_-), 0.86 (t, *J* = 6.9 Hz, 3H, -CH_2_-CH_3_). ^13^C NMR (75 MHz, DMSO-*d_6_*) δ 162.65 (4-C), 155.38 (2-C), 137.72 (6-C), 101.47 (5-C), 88.97 (1′-C), 84.06 (4′-C), 73.74 (2′-C), 69.46 (3′-C), 60.71 (5′-C), 40.21 (NH-CH_2_), 31.28, 29.02, 28.65, 26.50, 22.07, 13.90, 13.04 (NH-CH_2_-(CH_2_)_12_-CH_3_ + 5-CH_3_). HRMS (ESI) of C_21_H_37_N_3_O_5_, *m*/*z*: calcd [M + H]^+^ 412.2806, found: 412.2799.

*N*^4^-Tetradecylcytidine (**2e**). Prepared according to the general procedure from **5a** (0.185 g) and 1-tetradecylamine (0.128 g). Yield 0.158 g (72%). UV: λ_max_ = 272 nm. ^1^H NMR (300 MHz, DMSO-*d_6_*) δ 7.77 (d, *J* = 7.5 Hz, 1H, 6-H), 7.66 (t, *J* = 5.5 Hz, 1H, 4-NH), 5.76 (d, *J* = 3.6 Hz, 1H, 1′-H), 5.72 (d, *J* = 7.5 Hz, 1H, 5-H), 5.26 (d, *J* = 5.0 Hz, 1H, 2′-OH), 5.02 (t, *J* = 5.2 Hz, 1H, 5′-OH), 4.95 (d, *J* = 4.7 Hz, 1H, 3′-OH), 3.88–4.00 (m, 2H, 2′-H + 3′-H), 3.78–3.86 (m, 1H, 4′-H), 3.66 (ddd, *J* = 12.0, 5.1, 3.1 Hz, 1H, 5′-Ha), 3.54 (ddd, *J* = 12.0, 5.3, 3.5 Hz, 1H, 5′-Hb), 3.18–3.28 (m, 2H, NH-CH_2_-), 1.39–1.56 (m, 2H, NH-CH_2_-CH_2_-), 1.22–1.31 (m, 22H, NH-(CH_2_)_2_-(CH_2_)_11_-), 0.86 (t, *J* = 6.8 Hz, 3H, -CH_2_-CH_3_). ^13^C NMR (75 MHz, DMSO-*d_6_*) δ 163.28 (4-C), 155.43 (2-C), 140.12 (6-C), 94.55 (5-C), 89.20 (1′-C), 84.03 (4′-C), 73.98 (2′-C), 69.43 (3′-C), 60.65 (5′-C), 39.79 (NH-CH_2_), 31.30, 29.05, 28.71, 26.53, 22.09 (NH-CH_2_-(CH_2_)_8_-), 13.92 (-CH_2_-CH_3_). HRMS (ESI) of C_23_H_41_N_3_O_5_, *m*/*z*: calcd [M + H]^+^ 440.3119, found: 440.3115.

2′,3′,5′-Tri-*O*-acetyl-6-methyluridine (**8**). 6-Methyluracil (0.788 g, 6.2 mmol) was suspended with stirring in 25 mL of acetonitrile; bis-trimethylsilylacetamide (4 mL, 2.538 g, 3.0 mmol) was added, refluxed for 30 min, evaporated and then re-evaporated with toluene (2 × 5 mL). A solution of 1′,2′,3′,5′-tetra-*O*-acetylribose (1 g, 3.14 mmol) and trimethylsilyl triflate (0.763 g, 3.43 mmol) in acetonitrile (20 mL) was then added. The reaction mixture was refluxed for 3 h. After cooling, the reaction medium was sequentially treated with 5 mL of 50% aqueous pyridine. The resulting solution was evaporated, re-evaporated with toluene (2 × 5 mL), dissolved in chloroform (20 mL), and extracted with a saturated NaHCO_3_ aqueous solution, pure water and a saturated sodium chloride aqueous solution (5 mL each). The organic phase was dried with anhydrous sodium sulfate and evaporated. The product was isolated by column chromatography on silica gel using the system chloroform:ethyl acetate:ethanol (42.5:42.5:15). Yield 0.556 g (46%). ^1^H NMR (300 MHz, DMSO-*d_6_*) δ 11.29 (s, 1H, 3-H), 6.18 (d, *J* = 2.6 Hz, 1H, 1′-H), 5.66 (dd, *J* = 6.6, 2.6 Hz, 1H, 2′-H), 5.51 (s, 1H, 5-H), 5.49 (dd, *J* = 7.9, 6.6 Hz, 1H, 3′-H), 4.34 (dd, *J* = 11.6, 3.2 Hz, 1H, 5′-Ha), 4.14 (ddd, *J* = 7.9, 6.2, 3.2 Hz, 1H, 4′-H), 4.05 (dd, *J* = 11.6, 6.2 Hz, 1H, 5′-Hb), 2.00–2.08 (m, 12H, 3(CH_3_-COO-) + 6-CH_3_).

2′,3′,5′-Tri-*O*-acetyl-6-methyl-4-thiouridine (**10**). Lawesson’s reagent (0.235 g, 0.6 mmol) was added to the solution of 2′,3′,5′-tri-*O*-acetyl-6-methyluridine (8, 0.140 g, 0.4 mmol) dissolved in 15 mL of dioxane. The reaction mixture was refluxed for 3 h in an argon atmosphere and evaporated. The product was isolated by column chromatography on silica gel using the ethyl acetate: hexane (1:2) system. Yield 0.073 Г (50%). ^1^H NMR (300 MHz, CDCl_3_) δ 10.42 (s, 1H, 3-H), 7.50 (br. s, 1H, 5-H), 6.51 (m, 1H, 1′-H), 5.84 (dd, *J* = 6.7, 2.3 Hz, 1H, 2′-H), 5.60 (dd, *J* = 8.2, 6.7 Hz, 1H, 3′-H), 4.46 (dd, *J* = 11.4, 2.9 Hz, 1H, 5′-Ha), 4.28 (ddd, *J* = 8.2, 6.5, 2.9 Hz, 1H, 4′-H), 4.20 (dd, *J* = 11.4, 6.5 Hz, 1H, 5′-Hb), 2.06–2.13 (m, 12H, 3 (CH_3_-COO-) + 6-CH_3_); ^13^C NMR (75 MHz, CDCl_3_) δ 191.50 (4-C), 170.85, 170.08, 169.73 (3(-COO-)), 150.50 (2-C), 145.15 (6-C), 114.73 (5-C), 90.62 (1′-C), 78.78 (4′-C), 73.20 (2′-C), 70.25 (3′-C), 63.64 (5′-C), 20.86, 20.66, 20.51 (3(CH_3_-COO-)), 18.27 (6-CH_3_).

6-Methyl-4-thiouracil (**10**). 6-Methyluracil (0.2 g, 1.8 mmol) and Lawesson’s reagent (0.866 g, 2.1 mmol) were dissolved in a mixture of dioxane and pyridine (20 mL, 1:1). The reaction mixture was refluxed. The extent of the reaction was monitored using TLC, and then the reaction mixture was evaporated and suspended in water (25 mL). The precipitate that formed was filtered off, washed with water (50 mL) and ethyl acetate (200 mL), and then dried in a vacuum desiccator. Yield 0.193 Г (84%). ^1^H NMR (300 MHz, DMSO-*d*_6_) δ 12.20 (s, 1H, 1-H), 11.49 (s, 1H, 3-H), 6.14 (q, *J* = 1.33 Hz, 1H, 5-H), 2.03 (br. s, 3H, 6-CH_3_):

The general method for the synthesis of *N*^4^-alkyl-6-methylcytosines **11a**–**c**. 6-Methyl-4-thiouracil (10, 0.400 g, 2.7 mmol) was dissolved in 10 mL of ethylene glycol and the corresponding alkylamine (5.4 mmol) and 7-methylquinoline (0.550 mL, 0.604 g, 4.05 mmol) were added to the solution. The reaction mixture was refluxed. The extent of the reaction was monitored using the TLC method. After the completion of the reaction, the reaction mixture was evaporated and precipitated with water (25 mL); the precipitate that formed was filtered off and washed with water (50 mL) and ethyl acetate (200 mL) and then dried in a vacuum desiccator.

*N*^4^-Decyl-6-methylcytosine (**11a**). Yield 0.335 g (45%). UV: λ_max_ = 276 nm. ^1^H NMR (300 MHz, DMSO-*d*_6_) δ 10.20 (s, 1H, 1-H), 7.33 (t, *J* = 5.4 Hz, 1H, 4-NH), 5.38 (s, 1H, s, 1H, 5-H), 3.20 (td, *J* = 7.0, 5.4 Hz, 2H, -NH-CH_2_-), 1.97 (s, 3H, 6-CH_3_), 1.41–1.52 (m, 2H, -NH-CH_2_-CH_2_-), 1.23–1.34 (m, 14H, -NH-(CH_2_)_2_-(CH_2_)_7_-), 0.86 (t, *J* = 6.6 Hz, 3H, -CH_2_-CH_3_). HRMS (ESI) of C_15_H_27_N_3_O, *m*/*z*: calcd [M + H]^+^ 266.2227, found: 266.2234.

*N*^4^-Dodecyl-6-methylcytosine (**11b**). Yield 0.730 g (79%). UV: λ_max_ = 276 nm. ^1^H NMR (300 MHz, DMSO-*d*_6_) δ 10.20 (s, 1H, 1-H), 7.33 (t, *J* = 5.4 Hz, 1H, 4-NH), 5.38 (s, 1H, 5-H), 3.20 (td, *J* = 7.0, 5.4 Hz, 2H, -NH-CH_2_-), 1.97 (s, 3H, 6-CH_3_), 1.41–1.54 (m, 3H, -NH-CH_2_-CH_2_-), 1.21–1.29 (m, 18H, -NH-(CH_2_)_2_-(CH_2_)_9_-), 0.84 (t, *J* = 6.6 Hz, 3H, -CH_2_-CH_3_). HRMS (ESI) of C_17_H_31_N_3_O, *m*/*z*: calcd [M + H]^+^ 294.2540, found: 294.2539.

*N*^4^-Tetradecyl-6-methylcytosine (**11c**). Yield 1.05 g (82%). UV: λ_max_ = 276 nm. ^1^H NMR (300 MHz, DMSO-*d*_6_) δ 10.28 (s, 1H, 1-H), 7.37 (t, *J* = 5.5 Hz, 1H, 4-NH), 5.40 (s, 1H, 5-H), 3.21 (td, *J* = 7.0, 5.5 Hz, 2H, -NH-CH_2_-), 1.97 (s, 3H, 6-CH_3_), 1.40–1.51 (m, 2H, -NH-CH_2_-CH_2_-), 1.21–1.28 (m, 22H, NH-(CH_2_)_2_-(CH_2_)_11_-), 0.83 (t, *J* = 6.6 Hz, 3H, -CH_2_-CH_3_). HRMS (ESI) of C_19_H_35_N_3_O, *m*/*z*: calcd [M + H]^+^ 322.2853, found: 322.2854.

Synthesis of 2′,3′,5′-tri-*O*-acetyl-*N*^4^-alkyl-6-methylcytidine (**12a**–**c**).

*N*^4^-alkyl-6-methylcytosine (0.5 mmol) was suspended with stirring in dichloroethane (10 mL) and bis-trimethylsilylacetamide (0.679 mL, 0.432 g, 0.5 mmol) was added, leaving the mixture at room temperature until the precipitate would not dissolve. Then the reaction mixture was evaporated to oil and re-evaporated with toluene; a solution of 1′,2′,3′,5′-tetraacetylribose (0.154 g, 0.5 mmol) and trimethylsilyl triflate (0.119 g, 0.097 mL, 1.0 mmol) was added in dichloroethane (10 mL). The reaction mixture was kept at 37 °C for several days. The extent of the reaction was monitored using the TLC method. The reaction was stopped by adding 50% aqueous pyridine (4 mL). The resulting solution was evaporated and re-evaporated twice with toluene (5 mL). The mixture was dissolved in chloroform (25 mL) and extracted with water (2 × 5 mL) and a saturated sodium chloride solution (5 mL). The organic phase was then dried with anhydrous sodium sulfate. The product was isolated by column chromatography on silica gel using the chloroform–alcohol system (60:1).

2′, 3′, 5′-Tri-*O*-acetyl-*N*^4^-decyl-6-methylcytidine (**12a**). Yield 0.178 g (68%). ^1^H NMR (300 MHz, DMSO-*d_6_*) δ 7.72 (t, *J* = 5.6 Hz, 1H, 4-NH), 5.49–5.66 (m, 3H, 1′-H, 2′-H, 5-H), 5.14–5.26 (m, 1H, 3′-H), 4.28–4.38 (m, 1H, 4′-H), 4.00–4.13 (m, 2H, 5′-Ha, 5′-Hb), 3.22 (td, *J* = 6.9, 5.6 Hz, 2H, -NH-CH_2_-), 2.17 (s, 3H, 6-CH_3_), 2.00–2.06 (m, 9H, 3(CH_3_-COO-)), 1.44–1.50 (m, 2H, -NH-CH_2_-CH_2_-), 1.22–1.30 (m, 14H, -NH-(CH_2_)_2_-(CH_2_)_7_-), 0.83–0.88 (m, 3H, -CH_2_-CH_3_).

2′,3′,5′-Tri-*O*-acetyl-*N*^4^-dodecyl-6-methylcytidine (**12b**). Yield 0.22 g (80%). ^1^H NMR (300 MHz, DMSO-*d*_6_) δ 7.70 (t, *J* = 5.6 Hz, 1H, 4-NH), 5.50–5.66 (m, 4H, 1′-H, 2′-H, 3′-H, 5-H), 4.26–4.38 (m, 1H, 4′-H), 4.03–4.21 (m, 2H, 5′-Ha, 5′-Hb), 3.22 (td, *J* = 6.9, 5.6 Hz, 2H, -NH-CH_2_-), 2.17 (s, 3H, 6-CH_3_), 1.98–2.10 (m, 6H, 2(CH_3_-COO-)), 2.01 (s, 3H, CH_3_-COO-), 1.42–1.52 (m, 2H, -NH-CH_2_-CH_2_-), 1.22–1.33 (m, 18H, -NH-(CH_2_)_2_-(CH_2_)_9_-), 0.81–0.91 (m, 3H, -CH_2_-CH_3_).

2′,3′,5′-Tri-*O*-acetyl-*N*^4^-tetradecyl-6-methylcytidine (**12c**). Yield 0.179 g (62%). ^1^H NMR (300 MHz, DMSO-*d*_6_) δ 7.70 (t, *J* = 5.6 Hz, 1H, 4-NH), 5.50–5.65 (m, 4H, 1′-H, 2′-H, 3′-H, 5-H), 4.27–4.39 (m, 1H, 4′-H), 4.05–4.19 (m, 2H, 5′-Ha, 5′-Hb), 3.22 (td, *J* = 6.9, 5.6 Hz, 2H, -NH-CH_2_-), 2.17 (s, 3H, 6-CH_3_), 1.98–2.10 (m, 9H, 3(CH_3_-COO-)), 1.42–1.52 (m, 2H, -NH-CH_2_-CH_2_-), 1.22–1.27 (m, 22H, -NH-(CH_2_)_2_-(CH_2_)_11_-), 0.81–0.91 (m, 3H, -CH_2_-CH_3_).

The general method for the synthesis of *N*^4^-alkyl-6-methylcytidines **3a**–**c**.

The corresponding 2′,3′,5′-tri-*O*-acetyl-*N*^4^-alkyl-6-methylcytidine (**12a**–**c**) was suspended in 4 mL of a mixture of an aqueous 25% solution of ammonia and ethanol (1:1 *v*/*v*). The reaction mixture was kept at 37 °C overnight. The resulting solution was evaporated. The compound was isolated using column chromatography eluting with a 9:1 chloroform–ethanol system.

*N*^4^-Decyl-6-methylcytidine (**3a**) (0.068 g, 0.1 mmol). Yield: 0.047 Г (90%). UV: λ_max_ 272 nm. ^1^H NMR (300 MHz, DMSO-*d*_6_) δ 7.61 (t, *J* = 5.6 Hz, 4-NH), 5.59 (s, 1H, 5-H), 5.47 (d, *J* = 4.7 Hz, 1H, 1′-H), 5.03 (d, *J* = 5.8 Hz, 1H, 2′-OH), 4.77– 4.93 (m, 2H, 5′-OH, 3′-OH), 4.62 (ddd, *J* = 5.8, 5.5, 4.7 Hz, 1H, 2′-H), 4.14 (ddd, *J* = 5.8, 5.7, 5.5 Hz, 1H, 3′-H), 3.55– 3.78 (m, 3H, 4′-H, 5′-Ha, 5′-Hb), 3.15–3.28 (m, 2H, -NH-CH_2_-), 2.23 (s, 3H, 6-CH_3_), 1.38–1.52 (m, 2H, -NH-CH_2_-CH_2_-), 1.21–1.34 (m, 14H, -NH-(CH_2_)_2_-(CH_2_)_7_-), 0.92–0.81 (m, 3H, -CH_2_-CH_3_). ^13^C NMR (75 MHz, DMSO-*d_6_*) δ 162.78 (4-C), 155.98 (2-C) 152.47 (5-C), 95.72 (6-C), 91.67 (1′-C), 85.10 (4′-C), 70.81 (2′-C), 70.13 (3′-C) 62.13 (5′-C), 39.71 (-NH-CH_2_-), 31.26, 28.99, 28.94, 28.77, 28.66, 26.45, 22.84, 22.06 (-NH-CH_2_-(CH_2_)_8_-), 19.83 (6-CH_3_), 13.90 (-CH_2_-CH_3_). HRMS (ESI) of C_20_H_35_N_3_O_5_, *m*/*z*: calcd [M + H]^+^ 398.2649, found: 398.2647.

*N*^4^-Dodecyl-6-methylcytidine (**3b**) (0.411 g, 0.7 mmol). Yield: 0.292 g (92%). UV: λ_max_ 272 nm. ^1^H NMR (300 MHz, DMSO-*d*_6_) δ 7.64 (t, *J* = 5.6 Hz, 1H, 4-NH), 5.59 (s, 1H, 5-H), 5.48 (d, *J* = 4.6 Hz, 1H, 1′-H), 5.05 (d, *J* = 5.8 Hz, 1H, 2′-OH), 4.79–4.94 (m, 2H, 5′-OH, 3′-OH), 4.62 (ddd, *J* = 5.8, 5.5, 4.6 Hz, 1H, 2′-H), 4.14 (ddd, *J* = 6.2, 5.5, 4.6 Hz, 1H, 3′-H), 3.75 (ddd, *J* = 4.6, 4.6, 2.9 Hz, 1H, 4′-H), 3.61 (ddd, *J* = 11.7, 6.9, 2.9 Hz, 1H, 5′-Ha), 3.46 (ddd, *J* = 11.7, 6.9, 4.6 Hz, 1H, 5′-Hb), 3.18–3.28 (m, 2H, -NH-CH_2_-), 2.23 (s, 3H, 6-CH_3_), 1.39–1.51 (m, 2H, -NH-CH_2_-CH_2_-), 1.20–1.33 (m, 18H, -NH-(CH_2_)_2_-(CH_2_)_9_-), 0.81–0.91 (m, 3H, -CH_2_-CH_3_). ^13^C NMR (75 MHz, DMSO-*d*_6_) δ 162.76 (4-C), 155.97 (2-C) 152.56 (5-C), 95.74 (6-C), 91.71 (1′-C), 85.10 (4′-C), 70.84 (2′-C), 70.16 (3′-C), 62.20 (5′-C), 39.57 (-NH-CH_2_-), 31.28, 29.04, 29.01, 28.79, 28.69, 28.65, 26.47, 22.46, 22.08 ((-NH-CH_2_-(CH_2_)_10_-), 19.84 (6-CH_3_), 13.92 (-CH_2_-CH_3_). HRMS (ESI) of C_22_H_39_N_3_O_5_, *m*/*z*: calcd [M + H]^+^ 426.2962, found: 426.2960.

*N*^4^-Tetradecyl-6-methylcytidine (**3c**) (0.400 g, 0.7 mmol). Yield: 0.304 g (90%). UV: λ_max_ 272 nm. ^1^H NMR (300 MHz, DMSO-*d*_6_) δ 7.62 (t, *J* = 5.6 Hz, 1H, 4-NH), 5.59 (s, 1H, 5-H), 5.48 (d, *J* = 4.6 Hz, 1H, 1′-H), 5.03 (d, *J* = 5.8 Hz, 1H, 2′-OH), 4.77–4.93 (m, *J* = 12.8, 4.7 Hz, 2H, 5′-OH, 3′-OH), 4.62 (q, *J* = 5.8, 5.5, 4.7 Hz, 1H, 2′-H), 4.14 (ddd, *J* = 6.2, 5.5, 4.6 Hz, 1H, 3′-H), 3.75 (ddd, *J* = 4.6, 4.6, 2.9 Hz, 1H, 4′-H), 3.38–3.53 (m, 2H, 5′-Ha, 5′-Hb), 3.15–3.27 (m, 2H, -NH-CH_2_-), 2.23 (s, 3H, 6-CH_3_), 1.41–1.52 (m, 2H, -NH-CH_2_-CH_2_-), 1.21–1.34 (m, 22H, -NH-(CH_2_)_2_-(CH_2_)_11_-)_,_ 0.81–0.92 (m, 3H, -CH_2_-CH_3_). ^13^C NMR (75 MHz, DMSO-*d_6_*) δ 162.71 (4-C), 152.48 (2-C), 95.70 (5-C), 91.67 (6-C), 85.09 (1′-C), 79.13 (4′-C), 70.80 (2′-C), 70.11 (3′-C), 62.16 (5′-C), 39.57 (-NH-CH_2_-), 31.25, 29.00, 28.97, 28.77, 28.66, 28.62, 26.45, 22.04 (-NH-(CH_2_)_2_-(CH_2_)_12_-), 19.82 (6-CH_3_) 13.88 (-CH_2_-CH_3_). HRMS (ESI) of C_24_H_43_N_3_O_5_, *m*/*z*: calcd [M + H]^+^ 454.3275, found: 454.3276.

### 4.2. Enzymatic Synthesis of Nucleosides Using Nucleoside Phosphorylases

Enzymes: purine nucleoside phosphorylase (PNP), uridine phosphorylase (UP), thymidine phorphorylase (TP). The following recombinant *E. coli* enzymes [55] were used in the present study: UP with a specific activity of 100 units per mg of protein, 17 mg per mL; PNP 50 units per mg, 28 mg per mL, TP 80 units per mg, 4 mg per mL. Enzymes remain active in a mixture of DMF—potassium phosphate buffer (7 mM, pH 7.0), 3: 2 (*v*/*v*).

#### Enzymatic Reactions

1. To 0.5 mL of a 1 mM solution of compound **11c** in a solution of 60% DMF and 40% phosphate buffer (7 mM, pH 7.0), a 2-deoxyribose donor (2′-deoxyuridine 1.14 mg or 2′-deoxyadenosine 1.35 mg) was added to the concentration at 10 mM and dissolved with vigorous stirring. A total of 1 µL of PNP solution (1400 units of activity per mL, 1.4 units of activity) and 1 µL of UP (1700 units of activity per mL, 1.7 units of activity) were added. The reaction mixture was incubated at 50 °C in a thermostat for 4 days, and the control was carried out by HPLC by a joint injection with the starting compound **11c**.

2. To 0.36 mL of a solution of 0.18 mg of compound **12b** in DMF, 0.25 mL of a 1.75 mg solution of ribose donor adenosine hydrate in potassium phosphate buffer was added with stirring. The final volume of the reaction mixture was 0.61 mL, 60% DMF, **11c**, 1 mM, adenosine 10 mM. A total of 10 μL of PNP (14 activity units), UP (17 activity units), and TP (3.2 activity units) was added. The reaction mixture was incubated at 50 °C in a thermostat for 2 days.

### 4.3. Biological Evaluation

#### 4.3.1. Antibacterial Effect

##### Bacterial Strains

The following test strains were used [49,50]: Gram-positive bacteria *Bacillus subtilis* ATCC 6633, *Staphylococcus aureus* FDA 209P and INA 00761 (MRSA), and *Leuconostoc mesenteroides* VKPM B-4177; mycobacteria *Mycobacterium smegmatis* mc^2^ 155 and *M. smegmatis* VKPM Ac-1339; Gram-negative bacteria *E. coli* ATCC 25922 and *Pseudomonas aeruginosa* ATCC 27853 from the collection of the Gause Institute of New Antibiotics.

##### In Vitro Study of the Antibacterial Effect

Test strains were incubated in modified Gause’s nutrient medium № 2. The level of infection with test cultures was 10^6^ cells/mL. A compound being tested was dissolved in 30% aq. methanol. Ten volume percent of the tested compound was added to the nutrient medium. Samples without the addition of substances, antibiotics in medical use (amikacin, ciprofloxacin, isoniazid, rifampicin, oxacillin, and vancomycin), and samples of medium supplemented with a mixture of solvents served as controls of the test culture growth. *L. mesenteroides* was incubated at 28 °C, and all other strains were incubated at 37 °C.

#### 4.3.2. Fungal Growth Inhibition

##### Fungal Strains

All strains used in the work were isolated from the exhibits and surfaces of the halls of the Ancient Russian Paintings (56, 57, and 61) or in the Storage Fund of the main historical building of the State Tretyakov Gallery (10 Lavrushinsky per., Moscow, Russia) [15]. Twelve strains of filamentous fungi, previously isolated in the halls of the Ancient Russian Paintings (No. 56, 57, and 61) or in the Storage Fund, both located in the State Tretyakov Gallery (10 Lavrushinsky per., Moscow, Russia) [15] were used as test cultures to determine the antimycotic activity of studied compounds. *Aspergillus versicolor* STG-25G (SRX7729174; MK260015.1), *Mucor circinelloides* STG-30 (SRX7729212; MK260195.1), and *Ulocladium* sp. AAZ-2020a STG-36 (MW590700.1; SRX7729176) were isolated from the icon “the Church Militant” (dated 1550s). *Cladosporium halotolerans* STG-52B (SRX7729178; MK258720.1) was isolated from a bust fragment of the statue “Holy Great Martyr George the Victorious” (1464, Lime Stone, tempera). *Aspergillus creber* STG-57 (SRX7729151; MK266993.1) was isolated from the icon “Holy Great Martyr Demetrius of Thessaloniki” (dated 16th century). *Aspergillus versicolor* STG-86 (SRX7729182; MK262781.1), *Aspergillus creber* STG-93W (SRX7729186; MW575292.1), *Cladosporium parahalotolerans* STG-93B (SRX7729188; MK262909.1), and *Simplicillium lamellicola* STG-96 (SRX7729192; MK262921.1) were isolated from the surfaces of hall № 61. *Microascus paisii* STG-103 (SRX7729190; MW591474.1) was isolated from the hall № 57. *Aspergillus protuberus* STG-106 (SRX7729192; MK268342.1) was isolated from the hall № 56. *Penicillium chrysogenum* STG-117 (MW556011.1) was isolated from the surface of the icon ‘‘Prophet Solomon’’ (dated 1731).

##### Fungal Growth Inhibition

The filamentous fungi were grown on slants of Czapek-Dox agar (CDA) medium (30 g/L sucrose, 2 g/L NaNO_3_, 1 g/L K_2_HPO_4_, 0.5 g/L MgSO_4_ × 7 H_2_O, 0.5 g/L KCl, 0.01 g/L FeSO_4_ × 7 H_2_O, 20 g/l agar, pH 7.0–7.4). To determine the toxicity effect of **2a**–**2e**, **3b**, and **3c** on the mycelial growth, the drop-dilution method was used as described earlier with some modifications [56,57]. Cells were collected from agar slants and diluted with 0.9% NaCl solution up to OD600 = 0.5 (basic concentration), followed by a tenfold dilution with the same solvent (working concentration). Then, 3 μL of cell suspension from dilution 10-2 was inoculated onto Petri dishes with CDA prepared with or without the addition of alkyl-nucleosides (**1b**, **2a**–**2e**, **3b**, and **3c**), BAC (for positive control), NaPCP (for positive control) in the concentration 200 µM. The inoculated plates were incubated for 45 days at 26 °C. The inhibitory effects were measured every three days after inoculation and evaluated by the ratio of the colony growth on CDA medium supplemented with the relevant compound to the control growth (CDA medium without any additions). To determine the percent of fungal growth inhibition (FGI), we used the following formula: FGI % = [(Dc − Dt)/Dc] × 100 (1), where Dc indicates the colony diameter in the control set, and Dt indicates the colony diameter in the treatment set, as described earlier [58,59]. The data recorded were measured in triplicate and repeated at least twice. To determine the total antifungal activity against all test cultures (FGIav), we used the following formula: FGIav % = [(Dcav − Dtav)/Dcav] × 100 (2), where Dcav indicates the average diameter of colonies of all strains on a specific measurement day in the control set, and Dtav indicates the average diameter of colonies of all strains on the same day in the treatment set.

## 5. Conclusions

Our studies have shown that new *N*^4^-alkylcytidines are promising prototypes of biocides with a wide spectrum of action against both bacteria and mold fungi that destroy painting materials.

It turned out that the most active compounds, **1b** and **2b**, act on molds previously isolated in the State Tretyakov Gallery and are capable of damaging paintings, at the level of standard antiseptics used in modern restoration. This is very important for further research in order to expand the palette of antiseptics, which is necessary for the effective protection of cultural heritage works during various types of biodeterioration.

## Data Availability

Data are contained within the article and Supplementary Materials.

## Supplementary Materials

The following supporting information can be downloaded at: https://www.mdpi.com/article/10.3390/ijms25053053/s1.
