# Supplementary material for: New Biocides Based on N4-Alkylcytidines: Effects on Microorganisms and Application for the Protection of Cultural Heritage Objects of Painting
Source: Int J Mol Sci. 2024 Mar 6;25(5):3053. doi: 10.3390/ijms25053053 (PMC10931757; doi:10.3390/ijms25053053)
Supplement: Supplementary file 1 [file ijms-25-03053-s001.zip › ijms-2896261-supplementary.pdf]

New Biocides Based on N^4-alkylcytidines: Effects on Microorganisms and Application for the Protection of Cultural Heritage Objects of Painting

Liudmila A. Alexandrova[1*], Ivan A. Oskolsky[1], Dmitry A. Makarov[1], Maxim V. Jasko[1], Inna L. Karpenko[1], Olga V. Efremenkova[2], Byazilya F. Vasilyeva[2], Darya A. Avdanina[3], Anna A. Ermolyuk[3], Elizaveta E. Benko[3], Stanislav G. Kalinin[3], Tat'yana V. Kolganova[3], Maria Ya. Berzina[4], Irina D. Konstantinova[4], Alexander O. Chizhov[5], Sergey N. Kochetkov[1], Alexander A. Zhgun[3*]

[1] Engelhardt Institute of Molecular Biology RAS, 32 Vavilov str., Moscow 119991, Russia; ala2004_07@mail.ru (L.A.A.); oskosky@yandex.ru (I.A.O.); dmitmakarov_97@mail.ru (D.A.M.); 2003_maxim@mail.ru (M.V.J.); ikarikki@gmail.com (I.L.K.); snk1952@gmail.com (S.N.K.)

[2] Gause Institute of New Antibiotics RAMS, 11 Bol'shaya Pirogovskaya, Moscow 119021, Russia; bfvas@yandex.ru (B.F.V.); ovefr@yandex.ru (O.V.E.)

[3] Research Center of Biotechnology RAS, 33 Leninsky Ave, 119071, Moscow, Russia; d.avdanina@gmail.com (D.A.A.); anya_ermolyuk@mail.ru (A.A.E.); stanislav-kalinin-1990@mail.ru (S.G.K.); elizavetabenko8@gmail.com (E.E.B.); moldiag@biengi.ac.ru (T.V.K.); zzhgun@mail.ru (A.A.Z.)

[4] Shemyakin-Ovchinnikov Institute of Bioorganic Chemistry, 16/10 Miklukho-Maklaya str., Moscow, 117997, Russia; kid1968@yandex.ru (I.D.K.), berzina_maria@mail.ru (M. Ya. B.)

[5] Zelinsky Institute of Organic Chemistry RAS 47 Leninsky Ave, Moscow, 119991, Russia; chizhov@ioc.ac.ru (A.O.C.)

* Correspondence: ala2004_07@mail.ru (L.A.A.), tel. +7(926)547 1421; zzhgun@mail.ru (A.A.Z.), tel. +7 (916)9749769

Table of contents

Table S1 - Antibacterial activity	Page S2
Figure S1 - Enzymatic glycosylation of N^4-dodecyl-6-methylcytosine (**12b**)	Page S3
Figure S2 - NMR spectra of new compounds	Page S4
Figure S3 - Mass spectra of new compounds	Page S30

Table S1. Minimal bacterial growth inhibitory concentration (μg/ml) of N^4-alkylcytidine derivatives

Bacteria / Compounds	*Bacillus subtilis* ATCC 6633	*Staphylococcus aureus* FDA 209P	*Staphylococcus aureus* INA 00761	*Micrococcus luteus* NCTC 8340	*Leuconostoc mesenteroides* VKPM B-4177	*Mycobacterium smegmatis* VKPM Ac 1339	*Mycobacterium smegmatis* mc^{2}155
**1b**	16	16	16	16	16	16	16
**2a**	64	32	32	32	32	32	32
**2b**	8	8	8	8	8	16	16
**2c**	16	16	8	8	16	8	16
**2d**	32	8	8	16	16	32	32
**2e**	4	4	8	16	16	16	16
**3a**	>64	>64	>64	>64	>64	>64	>64
**3b**	8	8	8	32	8	32	32
**3c**	>64	>64	>64	>64	>64	>64	>64
**12a,12b**	n/a	n/a	n/a	n/a	n/a	n/a	n/a
Antibiotics used in clinics*	AN 4	AN 2, CIP 4, OX 1	AN 30, CIP 4, OX 32	AN 1.7	VA>400	AN 30 CIP 4, RFP 8, INZ 0.25	AN 85 RFP 4, INZ 4

*Amikacin (AN), Ciprofloxacin (CIP), Isoniazid (INZ), Rifampicin (RFP), Oxacillin (OX), Vancomycin (VA).

Figure S1. Enzymatic glycosylation of N^4-dodecyl-6-methylcytosine (**12b**)

BT

Project Name: TESTLast_1
Reported by User: System

Breeze

SAMPLE INFORMATION

Sample Name:	MBf-67 19h + Osk-5	Acquired By:	System
Sample Type:	Unknown	Date Acquired:	27.06.2023 11:54:02
Vial:	1	Acq. Method:	100B_dual_280
Injection #:	1	Date Processed:	27.06.2023 12:22:49
Injection Volume:	10,00 ul	Channel Name:	2487Channel 2
Run Time:	25,00 Minutes	Sample Set Name:	
Column Type:			

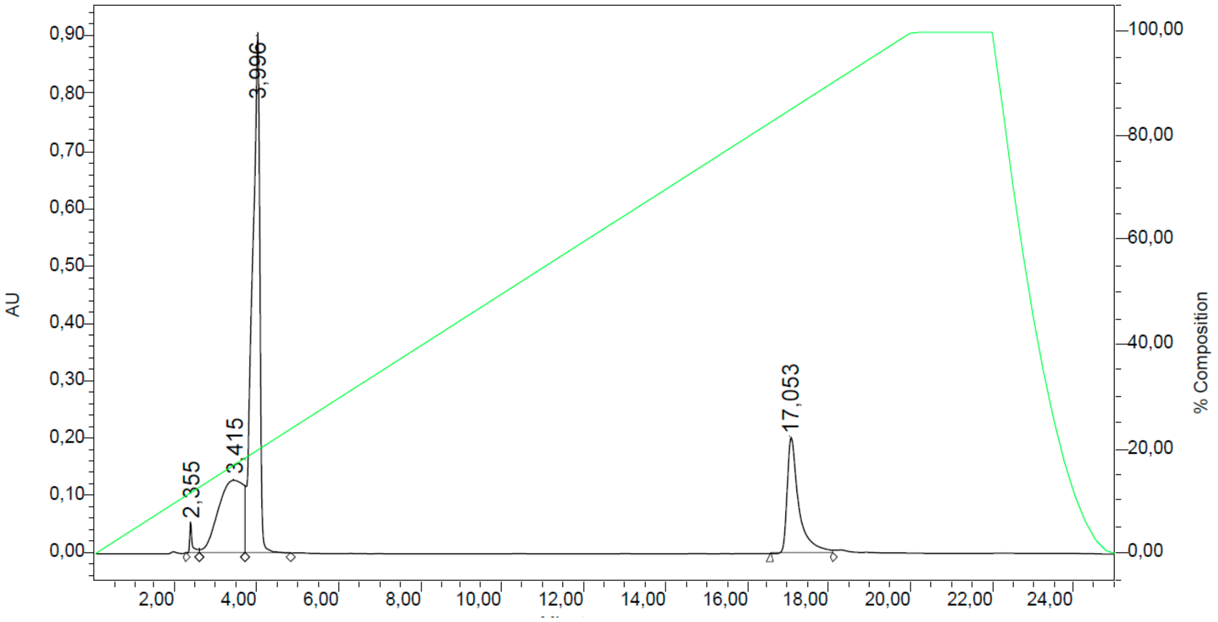

	RT (min)	Area (V*sec)	% Area	Height (V)	% Height
1	2,355	271420	1,27	51987	4,03
2	3,415	5621924	26,28	127774	9,90
3	3,996	11401358	53,29	907931	70,38
4	17,053	4098894	19,16	202367	15,69

Figure S1. Chromatogram of reaction mixture with purine nucleoside phosphorylase (PNP). Reaction involving adenosine (peak Rt 3.9 minutes) and N^4-dodecyl-6-methylcytosine (**12b**) (Rt 17.0 minutes). Adenine is also present in the reaction mixture (Rt 3.4 minutes), the peak is broadened due to the presence of DMF.

Figure S2. NMR spectra of new compounds

1) *N*[4]-decyl-5-methylcytidine (2a)

[1]H

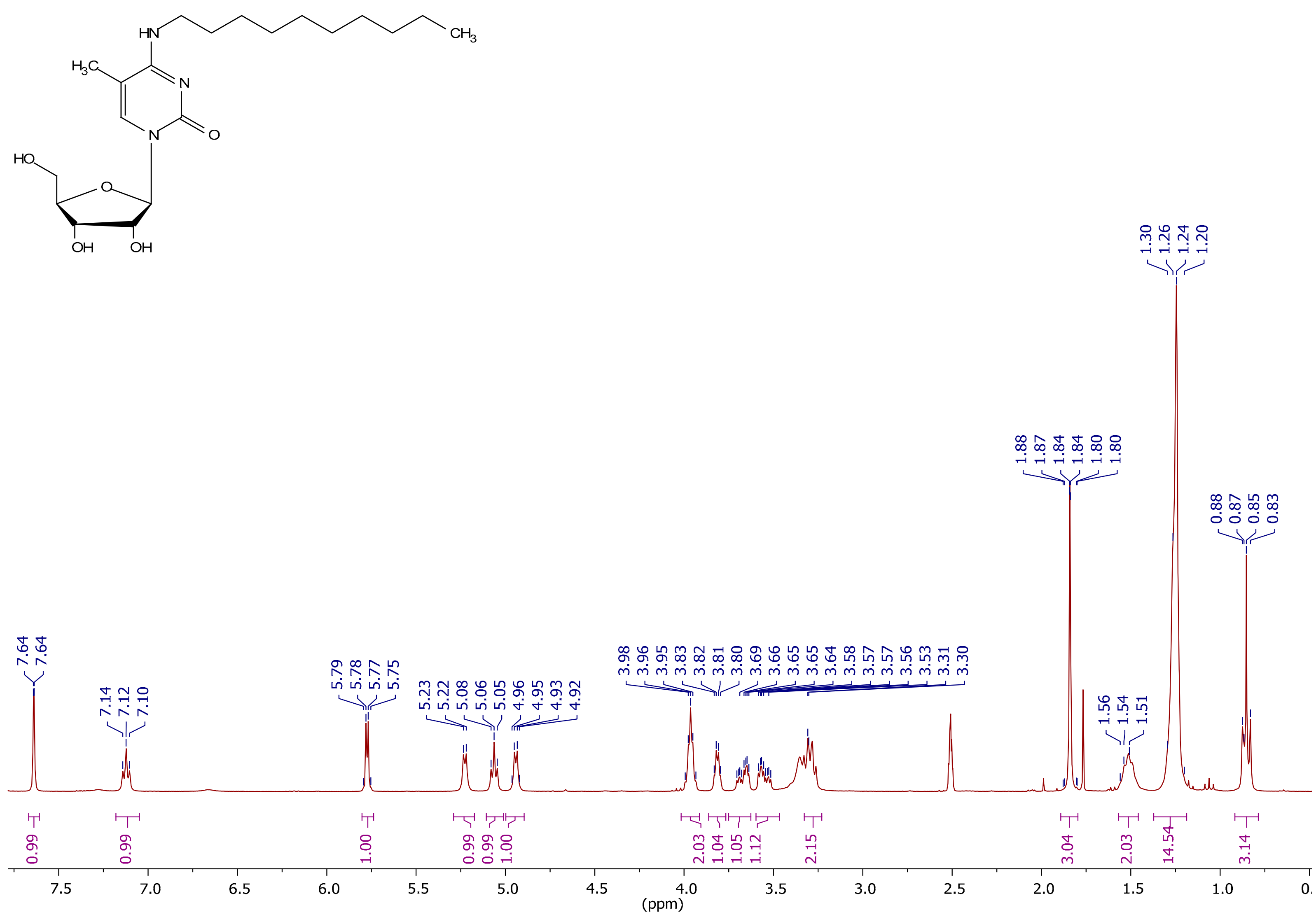

^{13}C

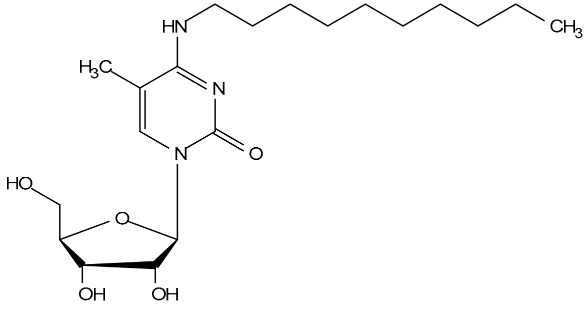
HN
CH3
H3C
N
N
O
HO
O
OH
OH

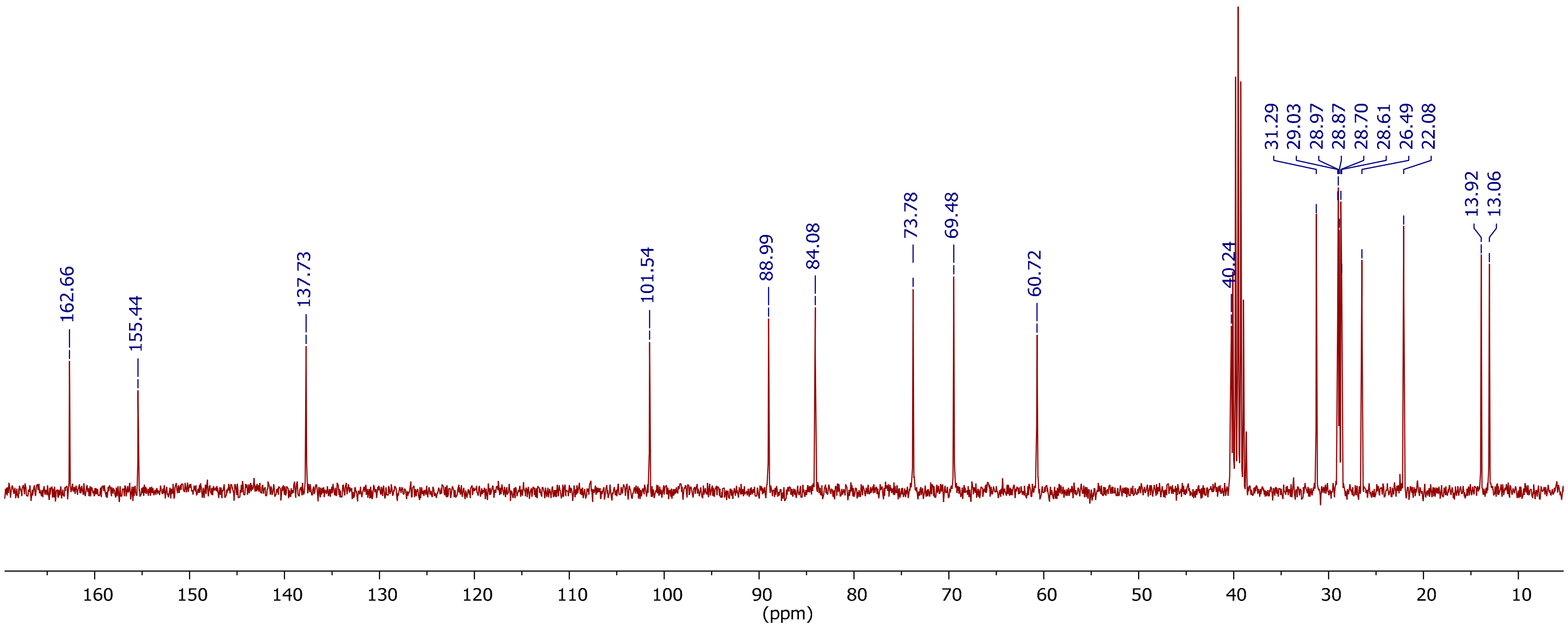
162.66
155.44
137.73
101.54
88.99
84.08
73.78
69.48
60.72
40.24
31.29
29.03
28.97
28.87
28.70
28.61
26.49
22.08
13.92
13.06
160
150
140
130
120
110
100
90
80
70
60
50
40
30
20
10
(ppm)

2) *N*⁴-dodecyl-5-methylcytidine (2b)

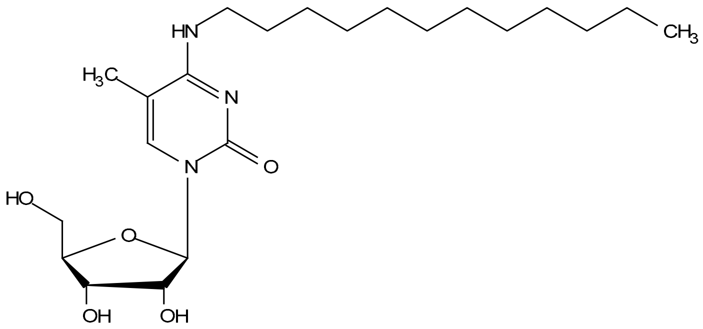

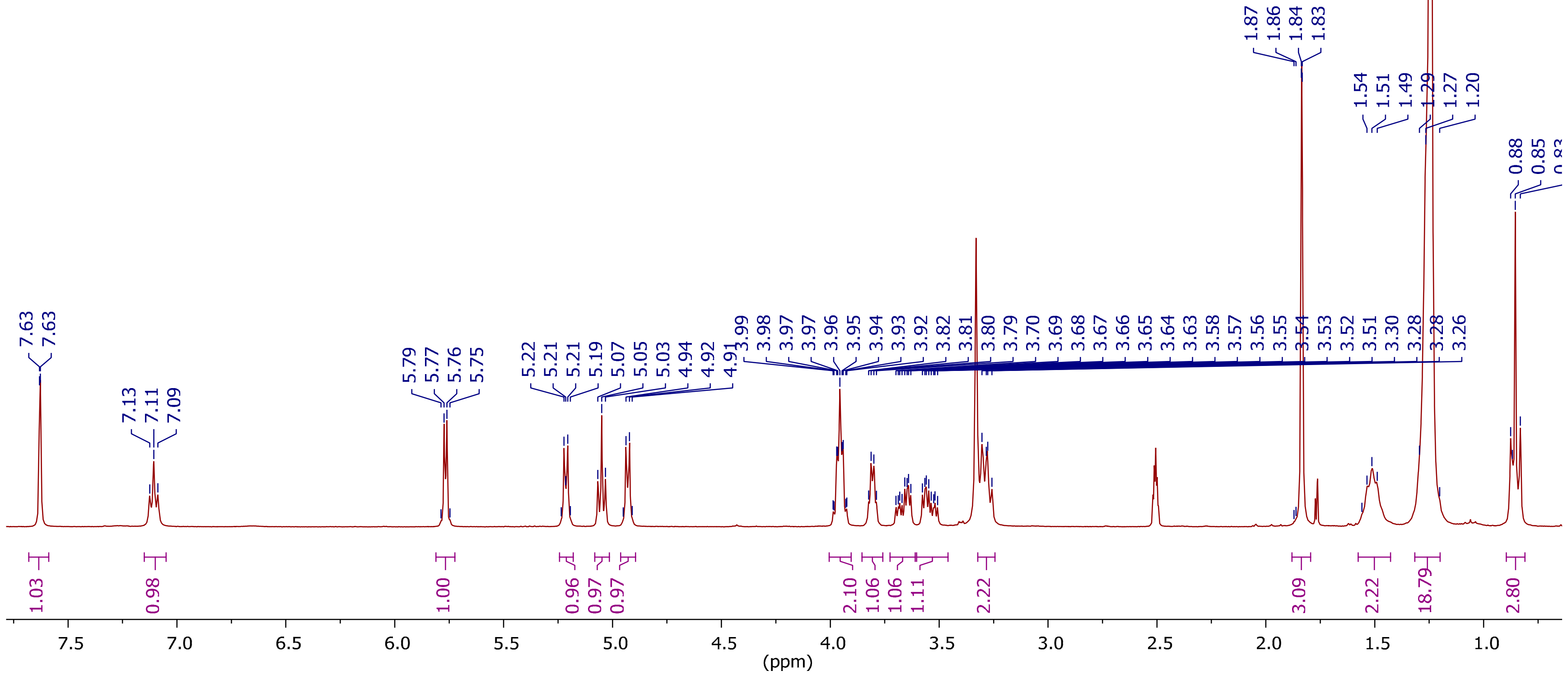

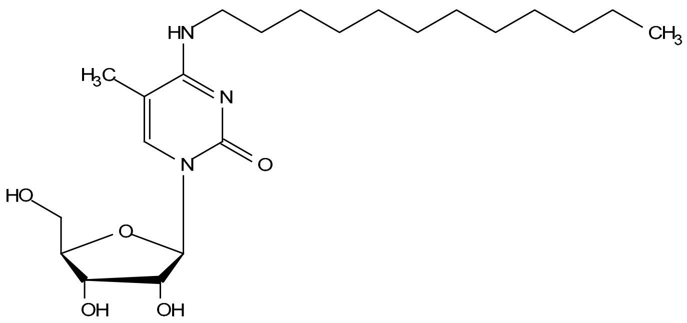
13C
HN
CH3
H3C
N
O
N
HO
O
OH
OH

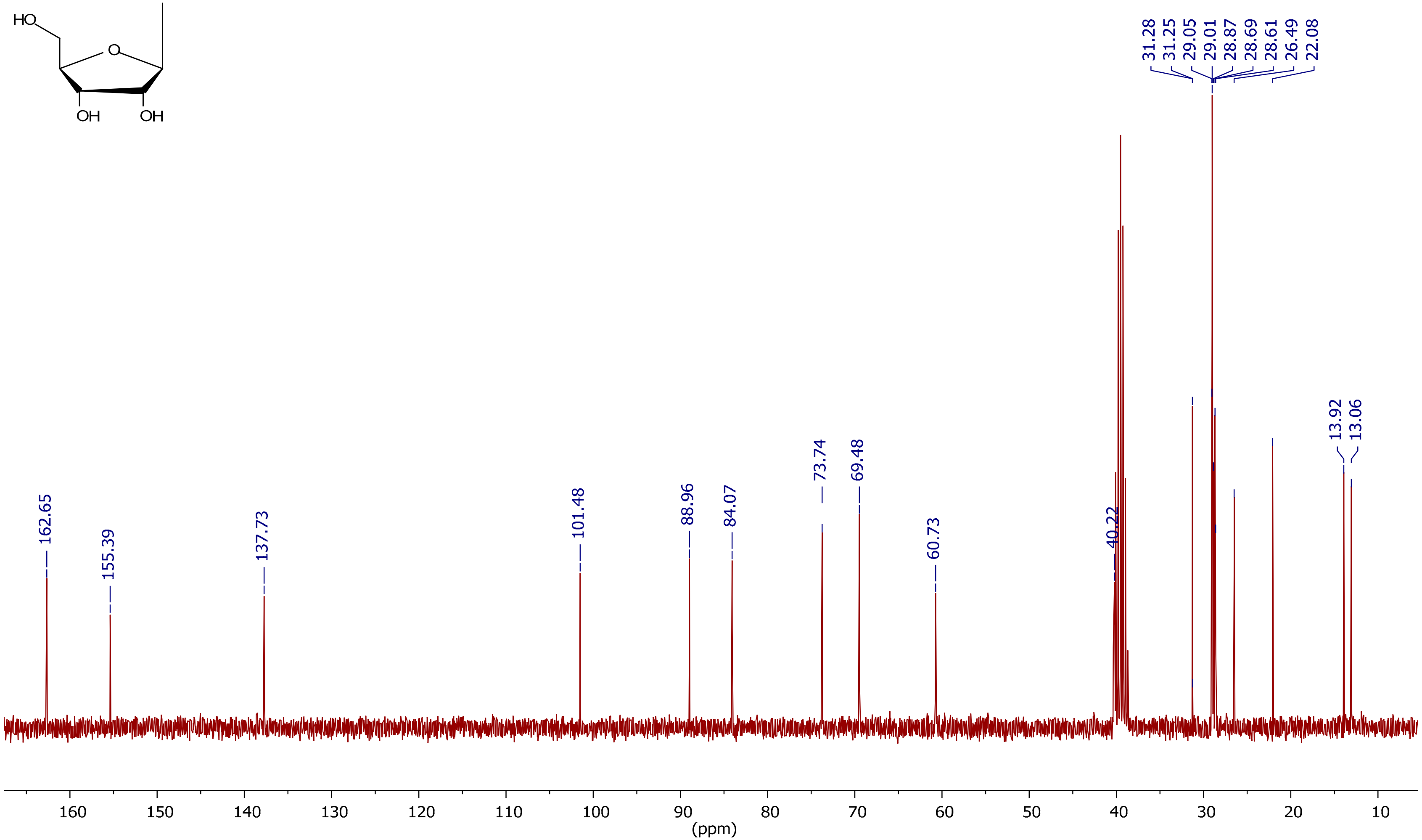
31.28
31.25
29.05
29.01
28.87
28.69
28.61
26.49
22.08
13.92
13.06
162.65
155.39
137.73
101.48
88.96
84.07
73.74
69.48
60.73
40.22
160
150
140
130
120
110
100
90
80
70
60
50
40
30
20
10
(ppm)

3) *N*[4]-tetradecyl-5-methylcytidine (2c)

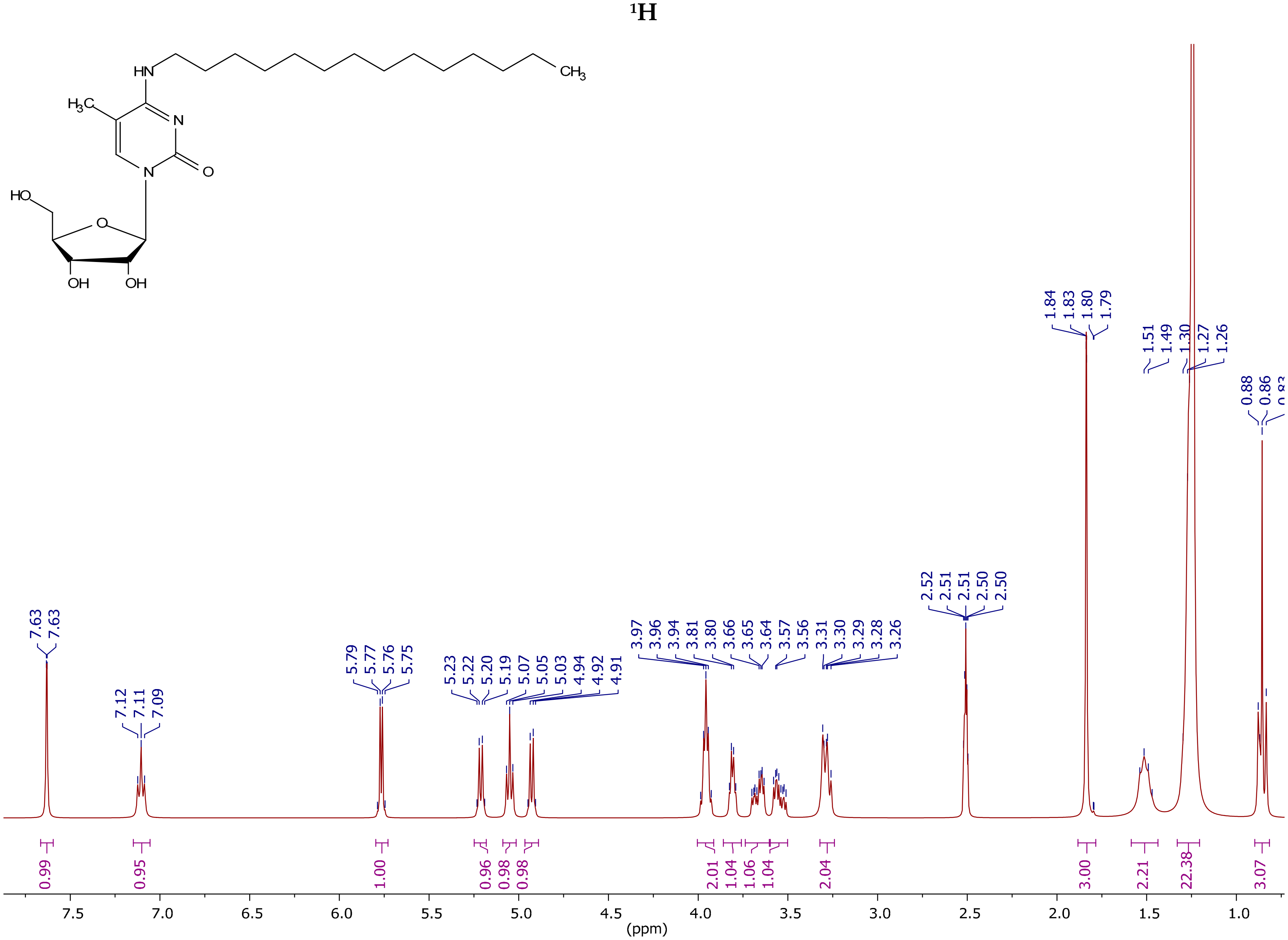

^{13}C

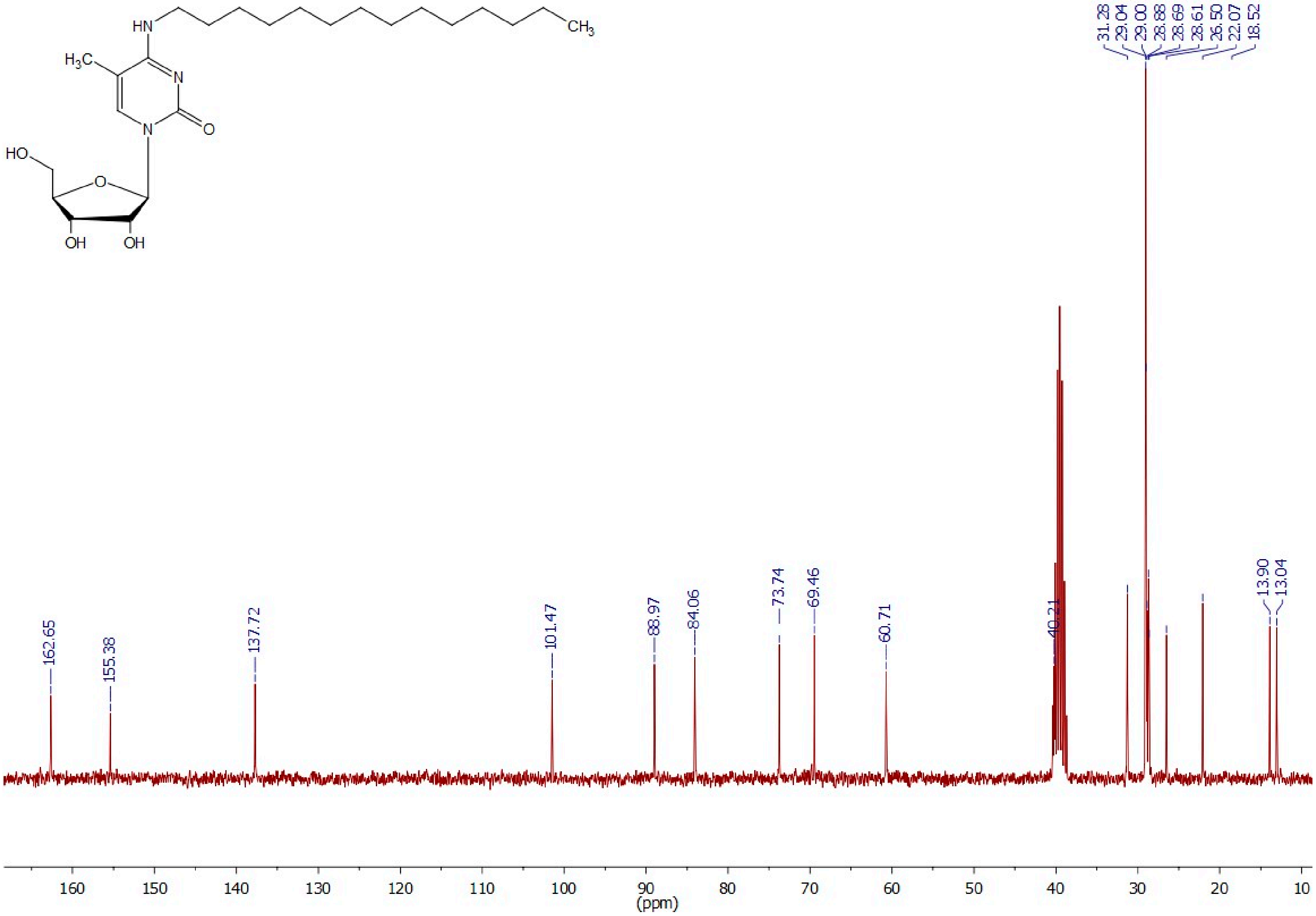

HN
CH3
H3C
N
N
O
HO
O
OH
OH
31.28
29.04
29.00
28.88
28.69
28.61
26.50
22.07
18.52
162.65
155.38
137.72
101.47
88.97
84.06
73.74
69.46
60.71
40.21
13.90
13.04
160
150
140
130
120
110
100
90
80
70
60
50
40
30
20
10
(ppm)

4) N^4-dodecylcytidine (2d)

^{1}H

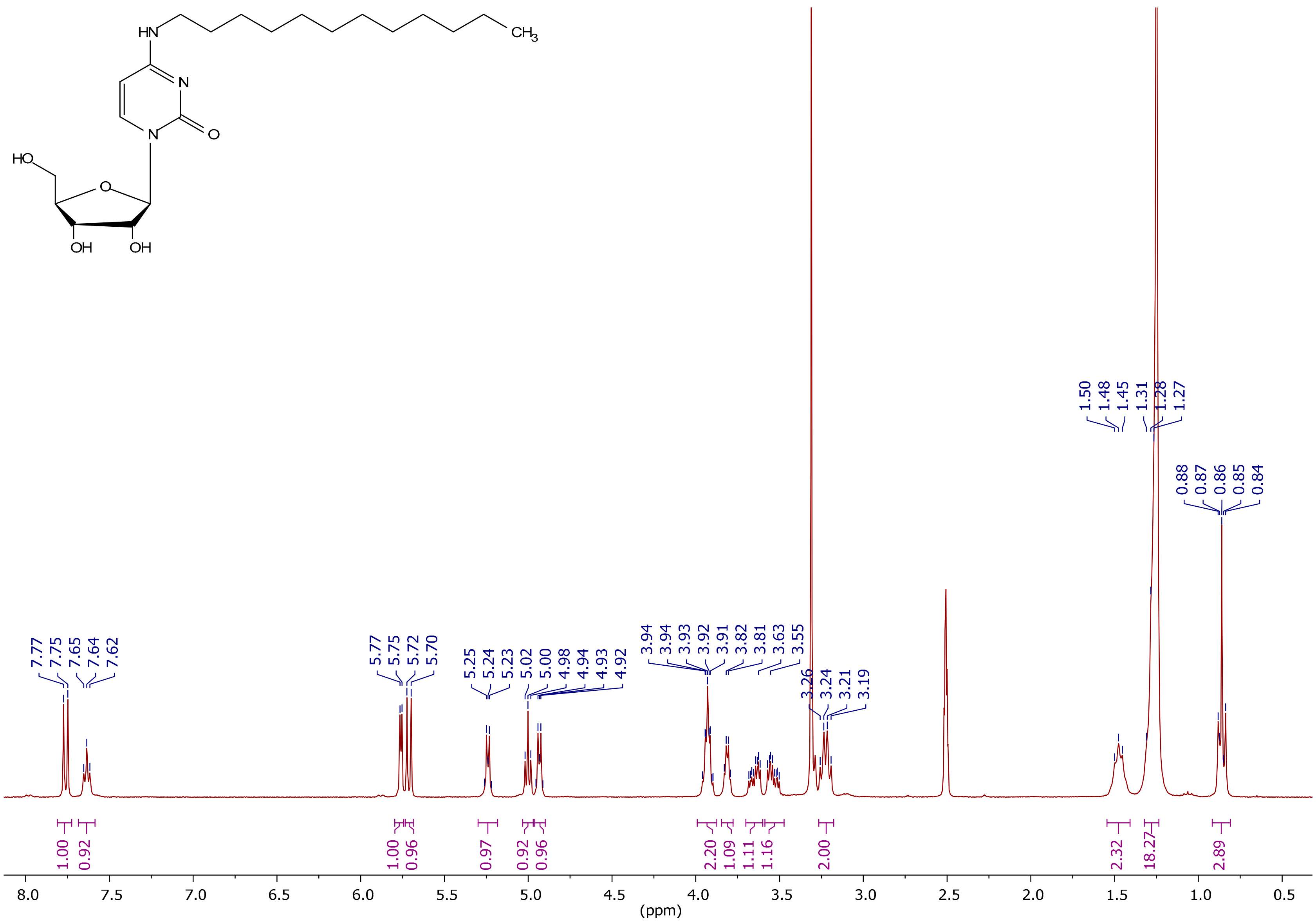

^{13}C

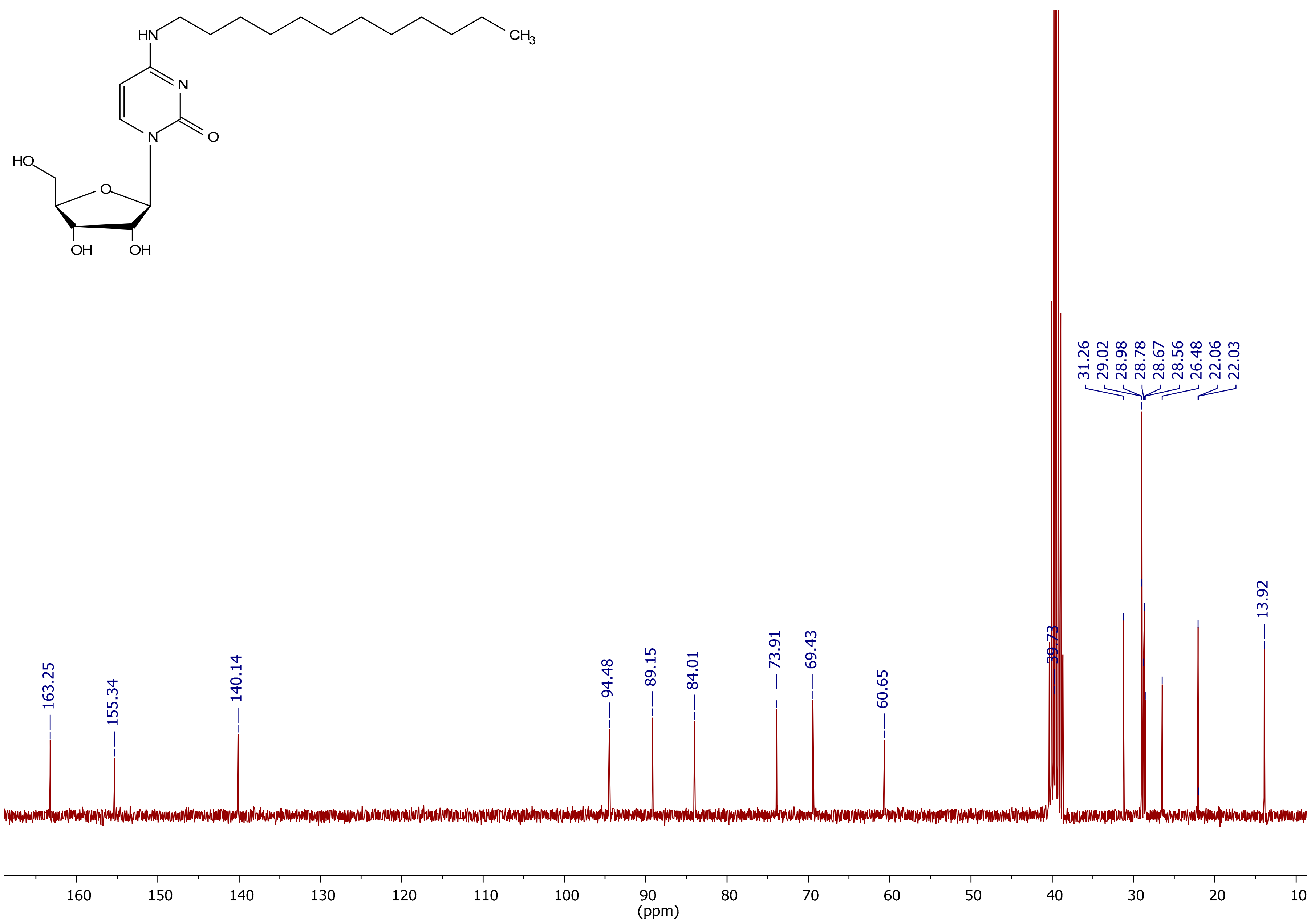
HN
CH3
N
N
O
HO
O
OH
OH
163.25
155.34
140.14
94.48
89.15
84.01
73.91
69.43
60.65
39.73
31.26
29.02
28.98
28.78
28.67
28.56
26.48
22.06
22.03
13.92
160 150 140 130 120 110 100 90 80 70 60 50 40 30 20 10
(ppm)

5) *N*[4]-tetradecylcytidine (2e)

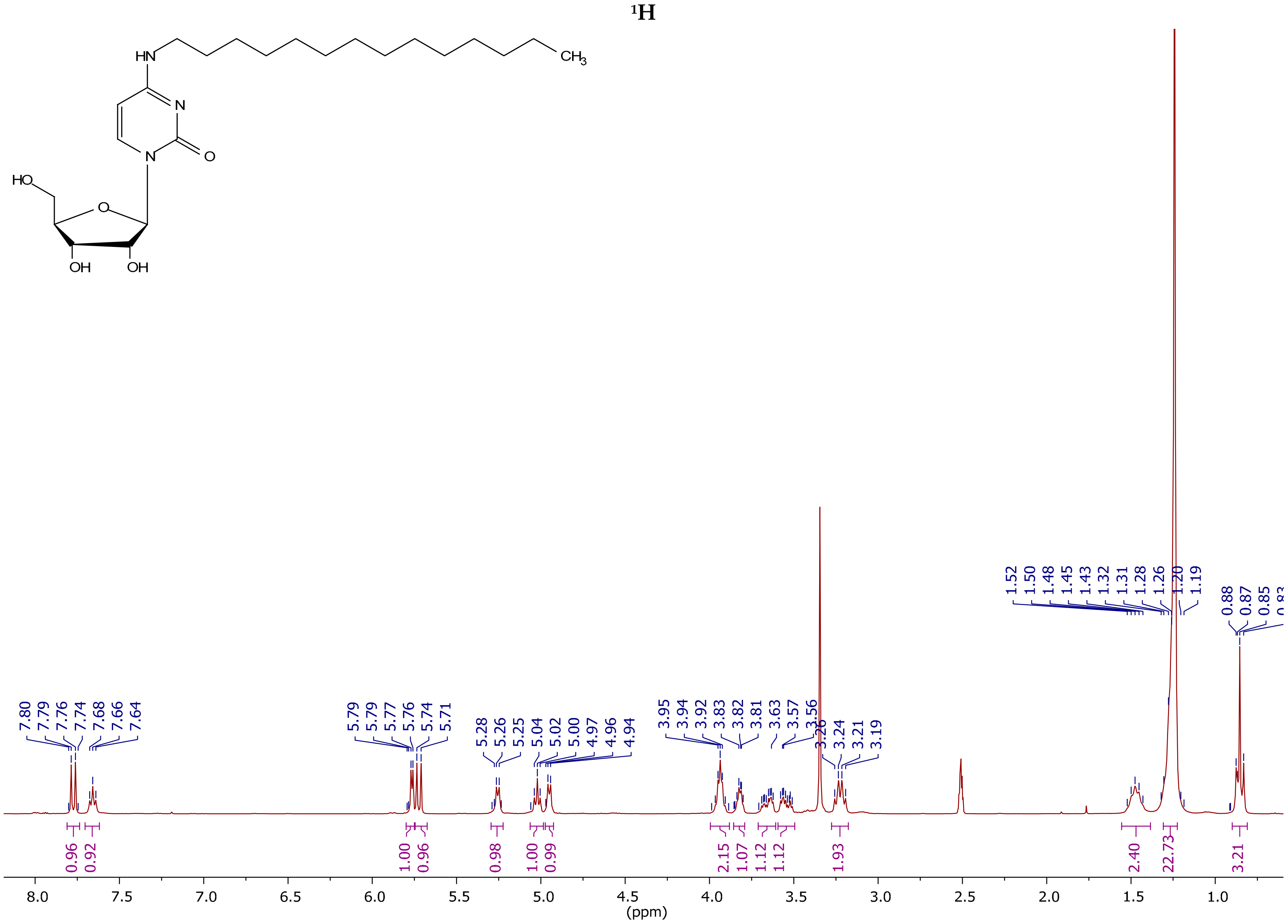

^{13}C

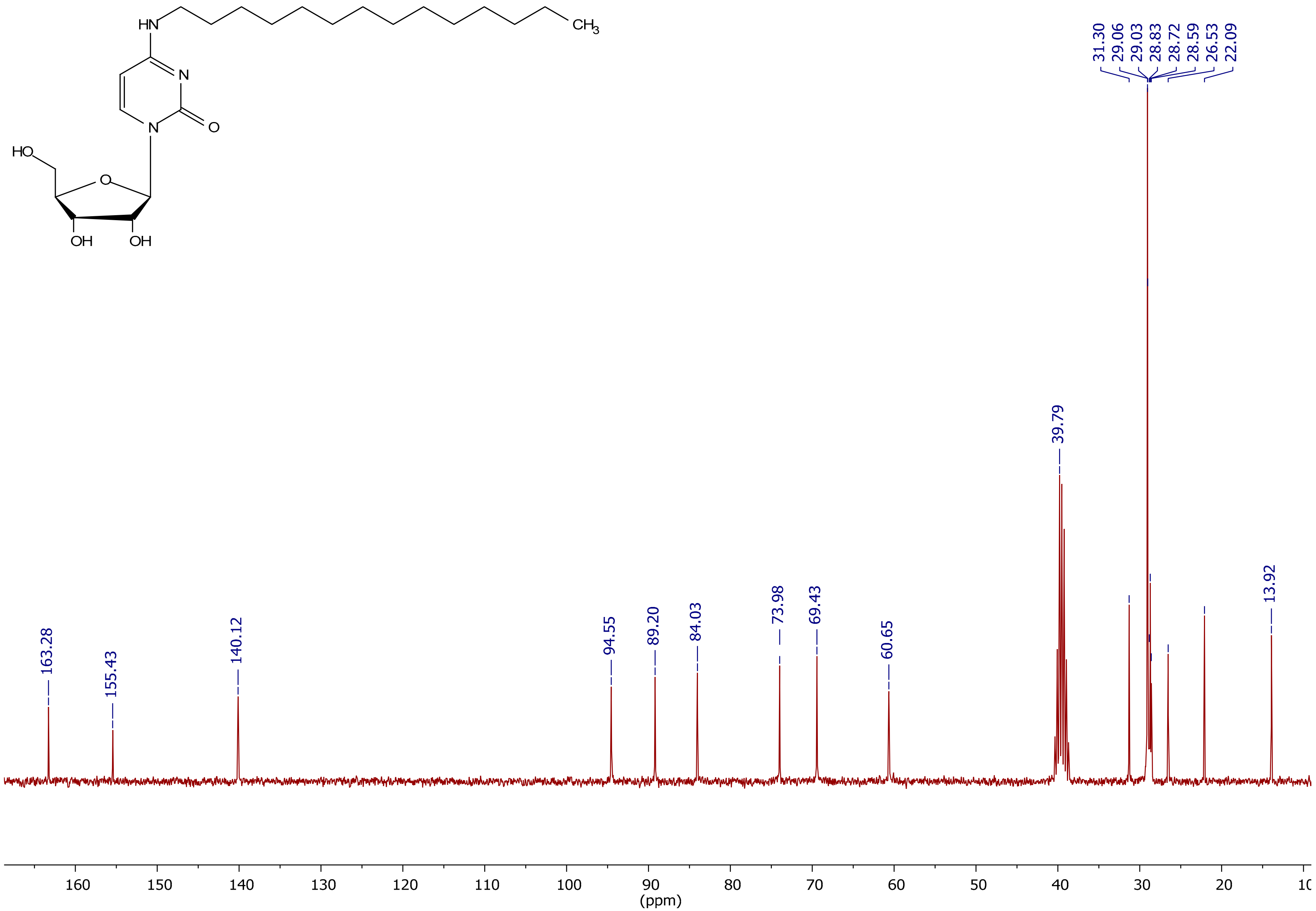
HN
CH3
N
N
O
HO
O
OH
OH
31.30
29.06
29.03
28.83
28.72
28.59
26.53
22.09
39.79
163.28
155.43
140.12
94.55
89.20
84.03
73.98
69.43
60.65
13.92
160
150
140
130
120
110
100
90
80
70
60
50
40
30
20
(ppm)

6) *N*[4]-Decyl-6-methylcytidine (3a)

^{1}H

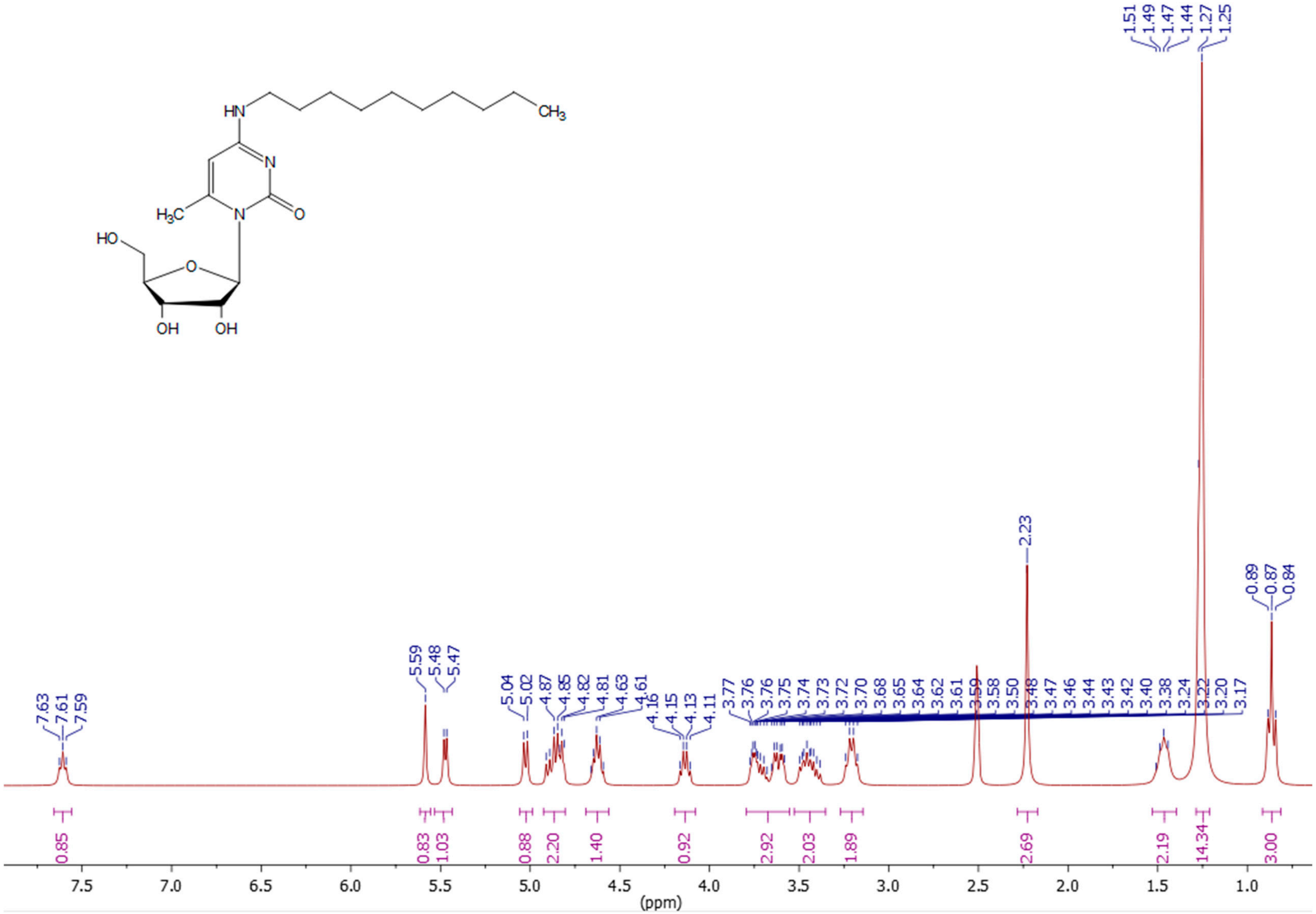

^{13}C

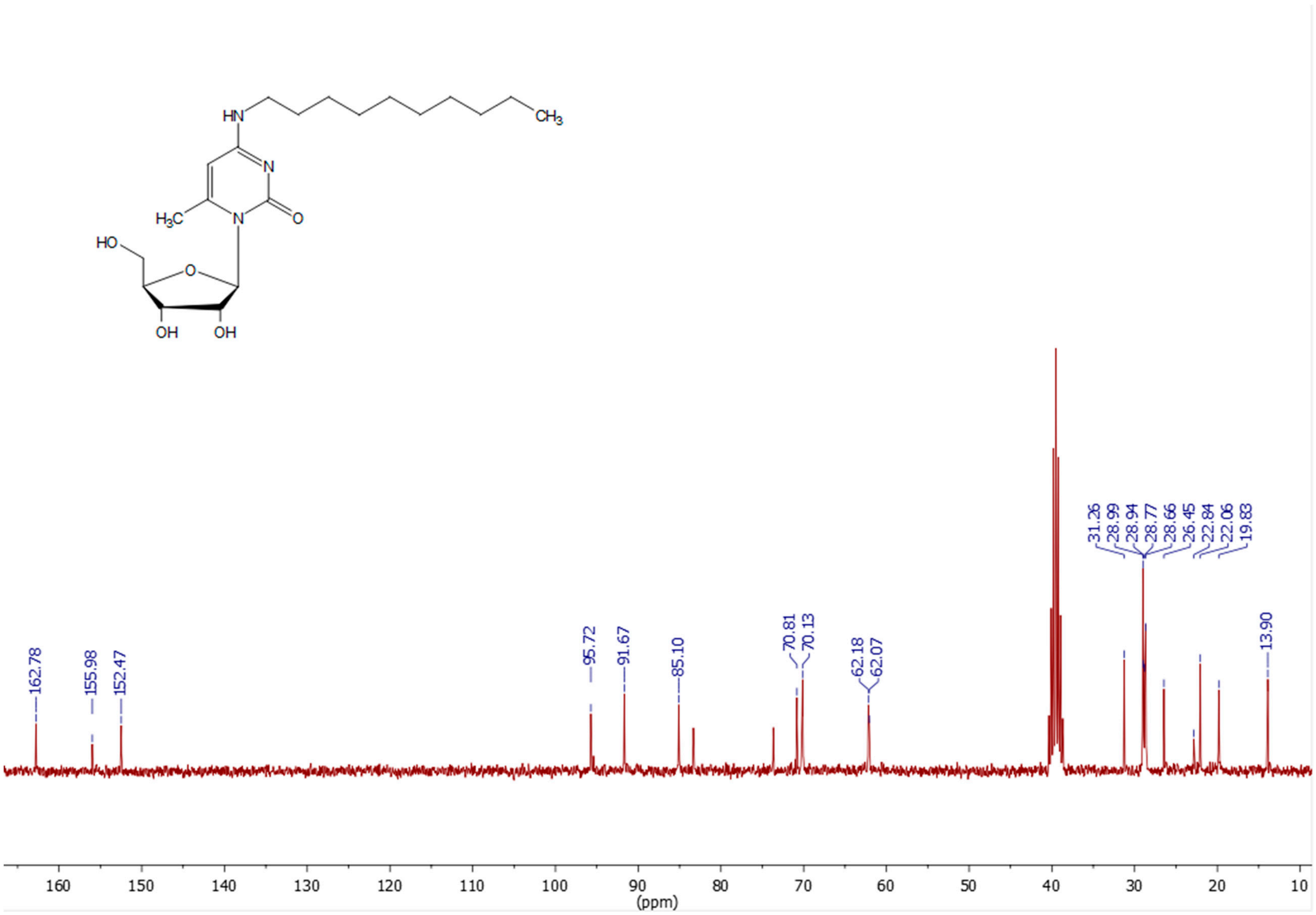
HN
CH3
N
H3C
N
O
HO
O
OH
OH
162.78
155.98
152.47
95.72
91.67
85.10
70.81
70.13
62.18
62.07
31.26
28.99
28.94
28.77
28.66
26.45
22.84
22.06
19.83
13.90
160
150
140
130
120
110
100
90
80
70
60
50
40
30
20
10
(ppm)

7) *N*⁴-Dodecyl-6-methylcytidine (3b)

¹H

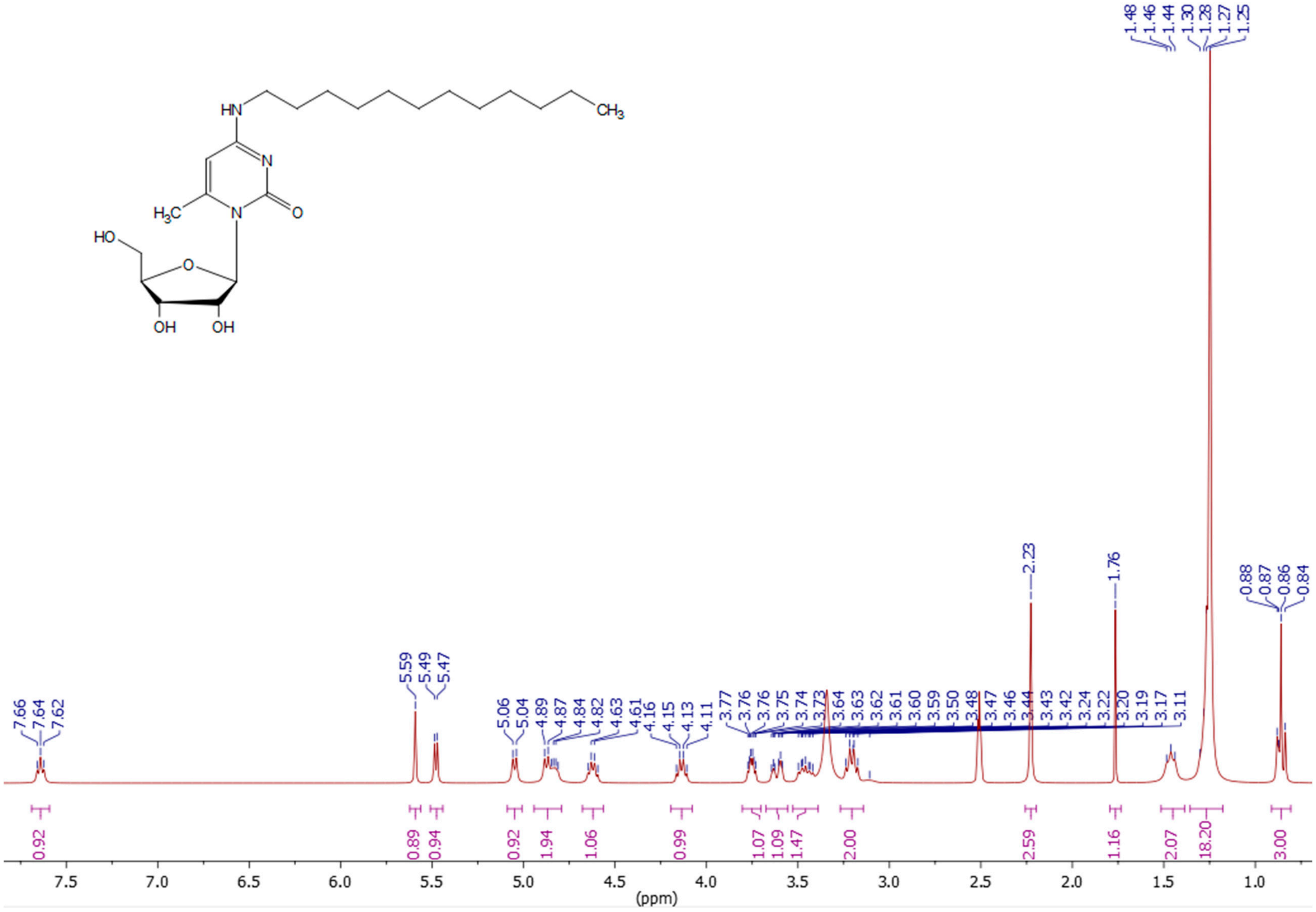

^{13}C

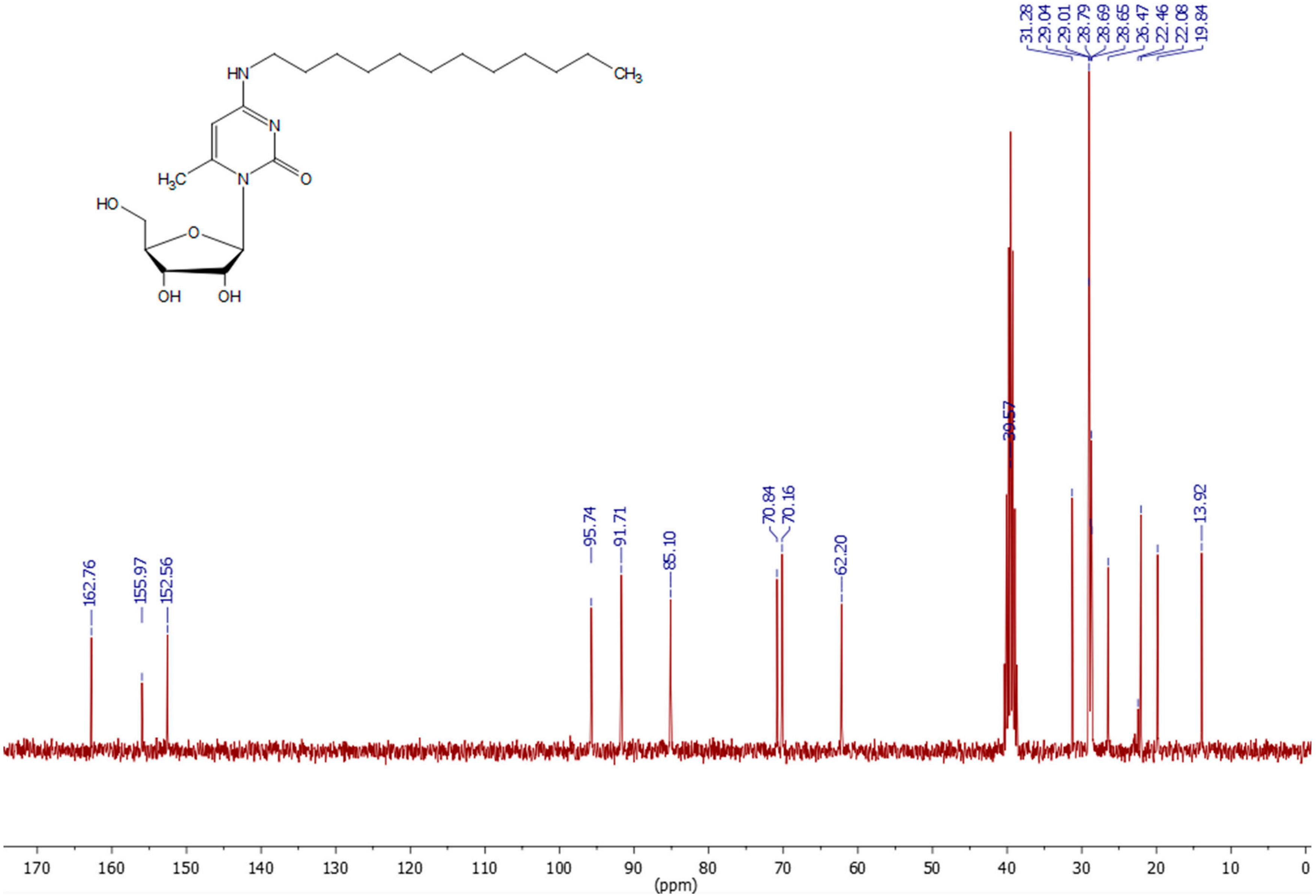
HN
CH3
N
O
H3C
N
HO
O
OH
OH
31.28
29.04
29.01
28.79
28.69
28.65
26.47
22.46
22.08
19.84
39.57
162.76
155.97
152.56
95.74
91.71
85.10
70.84
70.16
62.20
13.92
170
160
150
140
130
120
110
100
90
80
70
60
50
40
30
20
10
0
(ppm)

8) *N*⁴-Tetradecyl-6-methylcytidine (3c)

[1]H

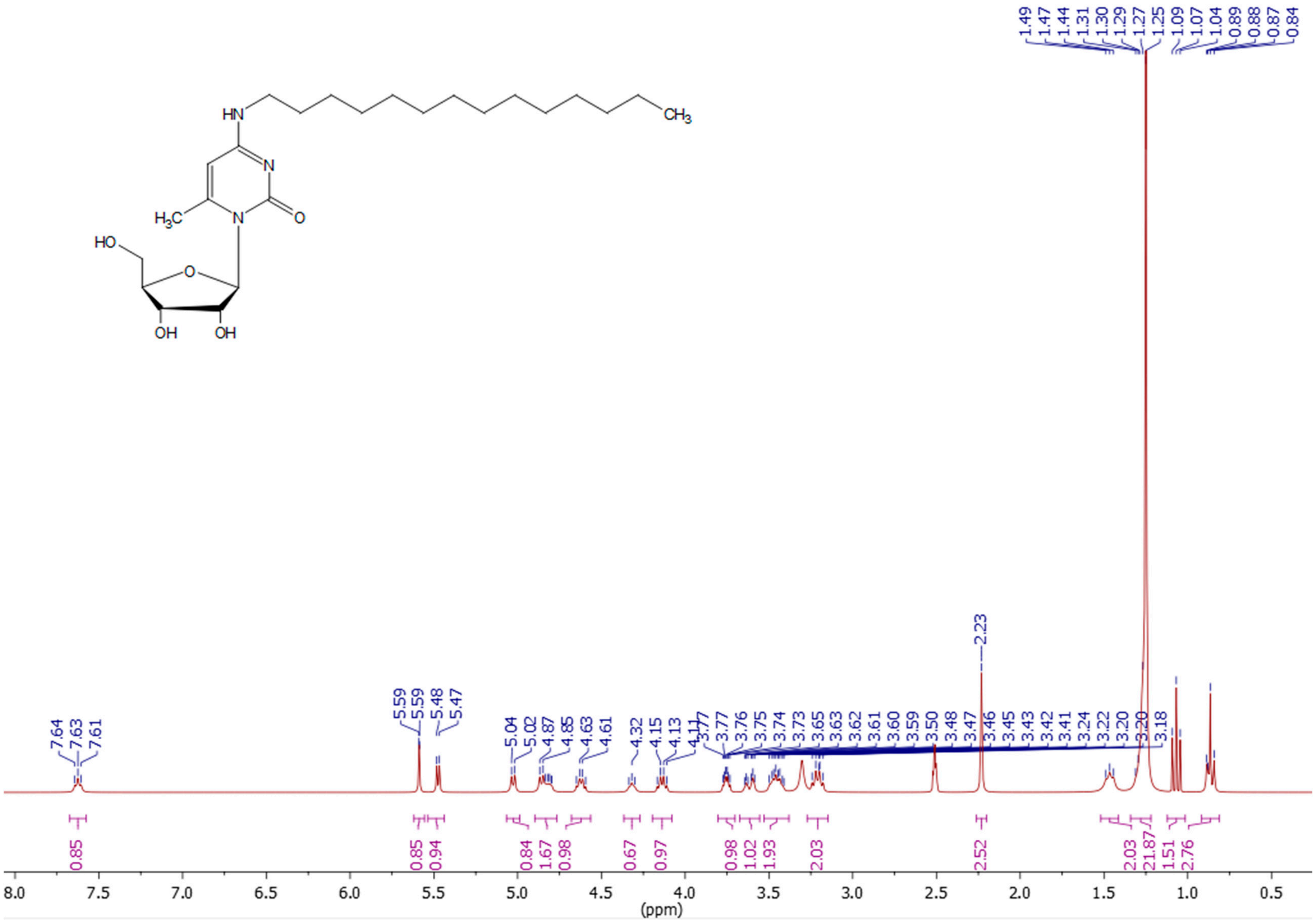

[13]C

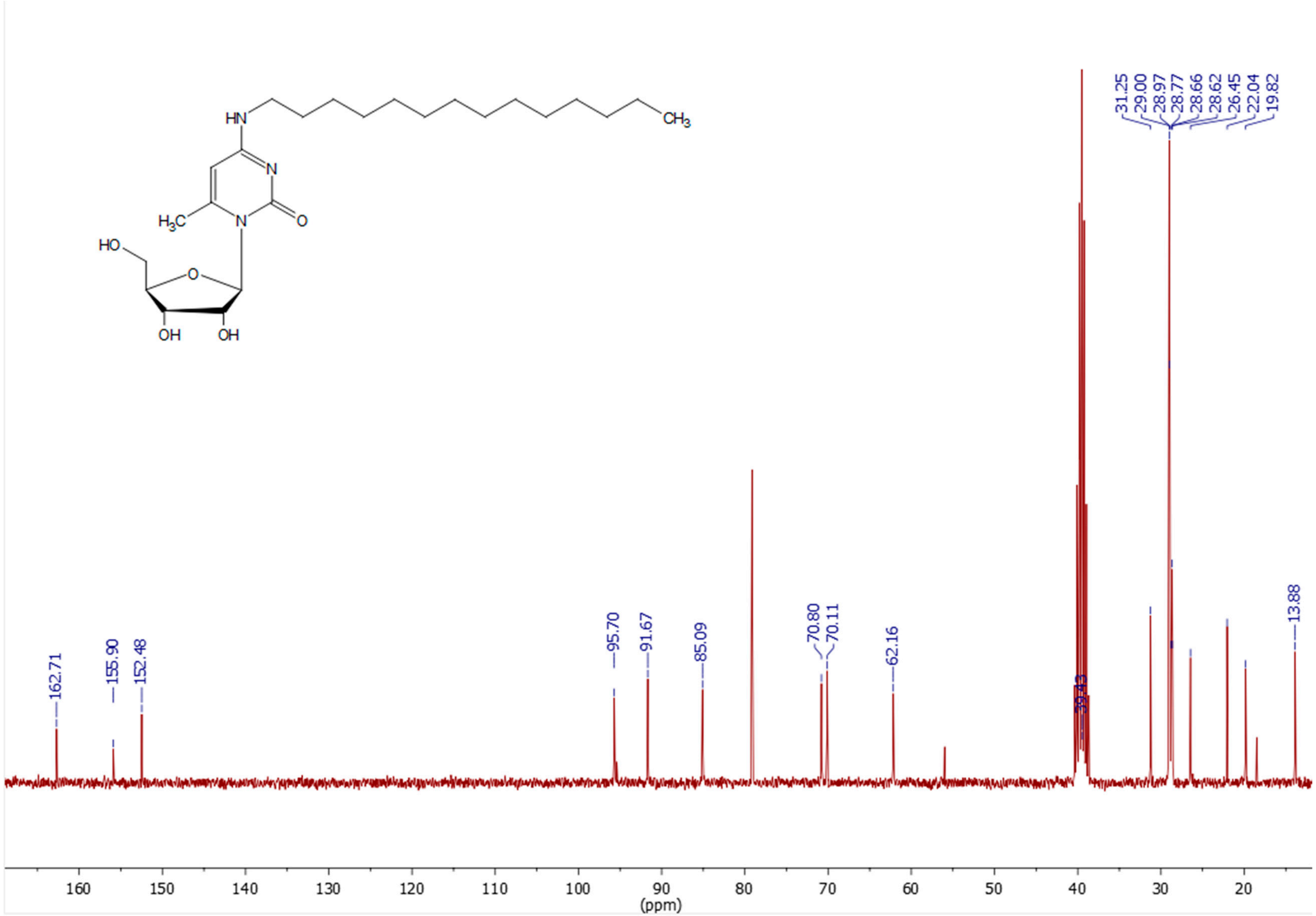

HN
CH3
N
H3C
N
O
HO
O
OH
OH
31.25
29.00
28.97
28.77
28.66
28.62
26.45
22.04
19.82
162.71
155.90
152.48
95.70
91.67
85.09
70.80
70.11
62.16
39.43
13.88
160
150
140
130
120
110
100
90
80
70
60
50
40
30
20
(ppm)

9) **2′,3′,5′-Tri-*O*-acetyl-6-methyluridine (8)**

[1]H

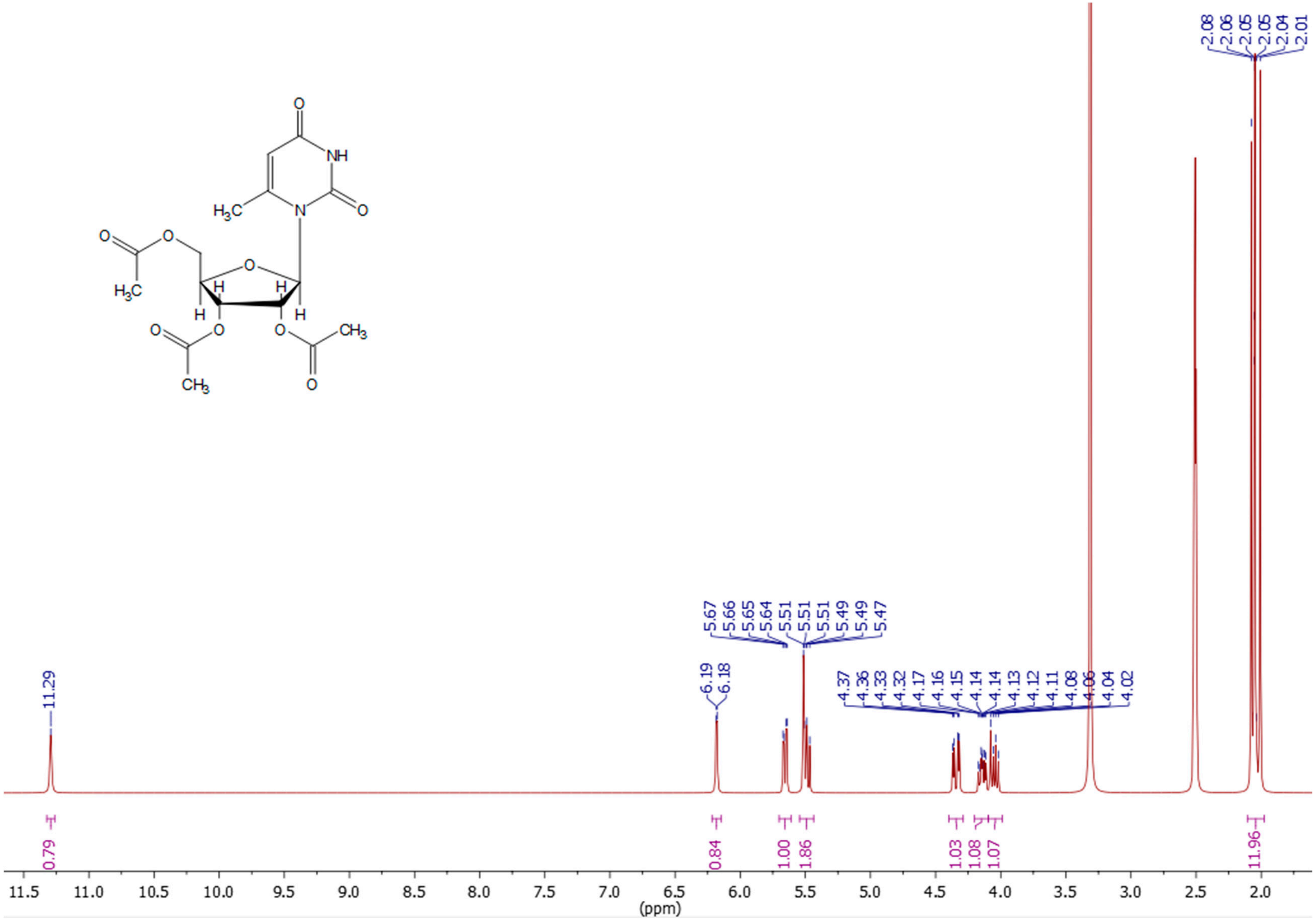

10) 2′,3′,5′-Tri-*O*-acetyl-6-methyl-4-thiouridine (10)

^{1}H

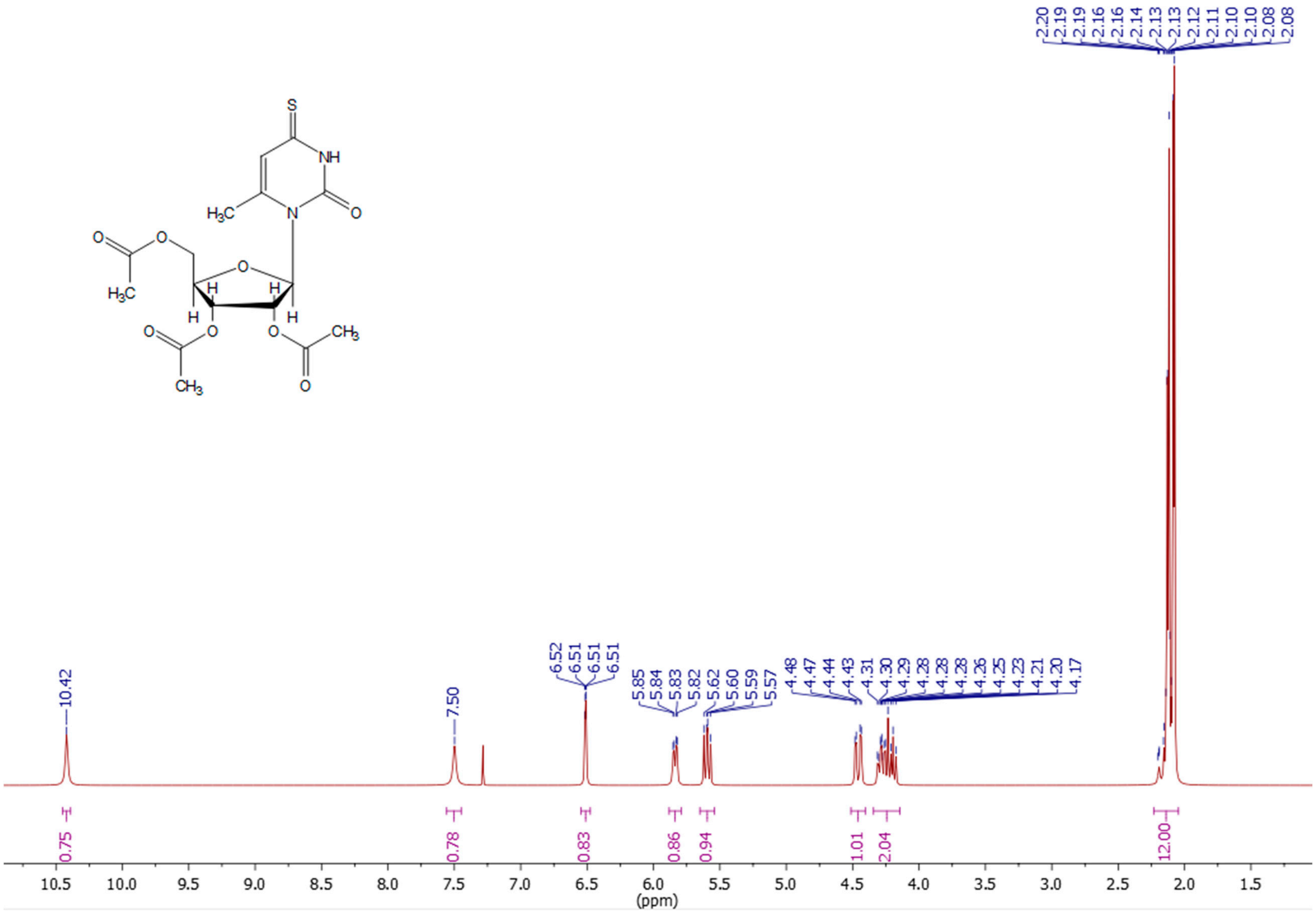

^{13}C

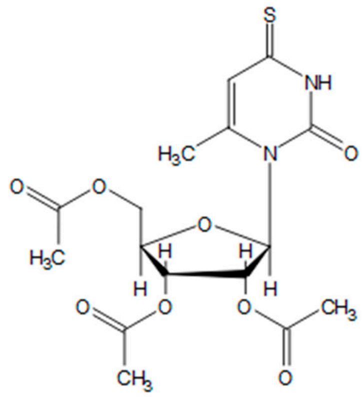
S
NH
O
N
H_3C
O
O
O
H_3C
H
H
H
H
O
O
O
CH_3
O
CH_3
O

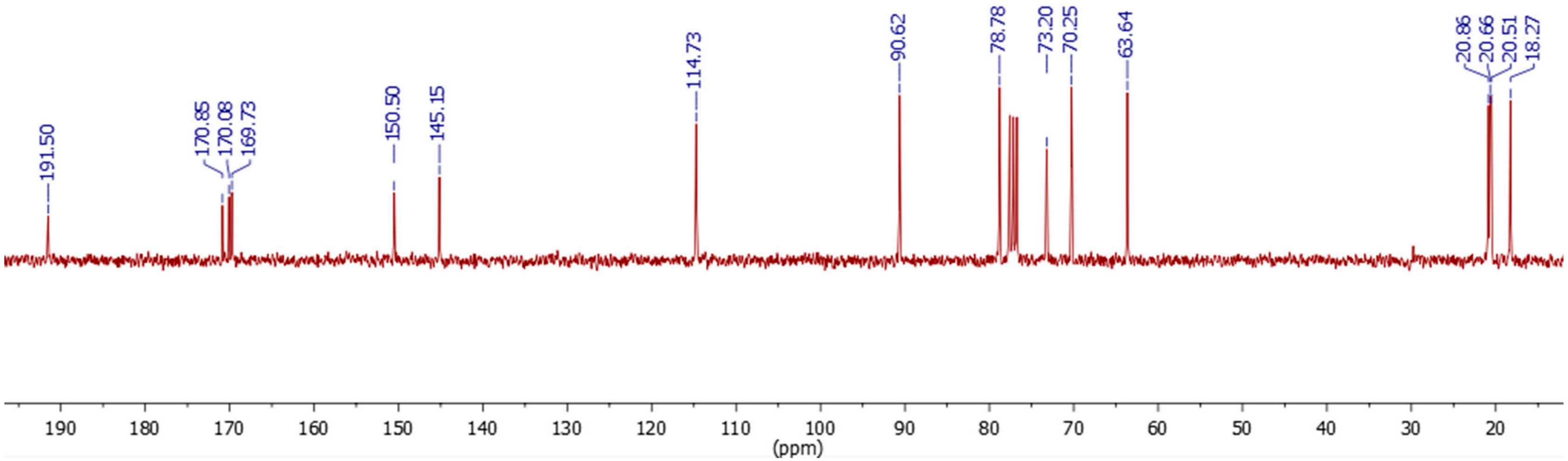
191.50
170.85
170.08
169.73
150.50
145.15
114.73
90.62
78.78
73.20
70.25
63.64
20.86
20.66
20.51
18.27
190
180
170
160
150
140
130
120
110
100
90
80
70
60
50
40
30
20
(ppm)

11) 4-Thio-6-methyluracyl (11)

^{1}H

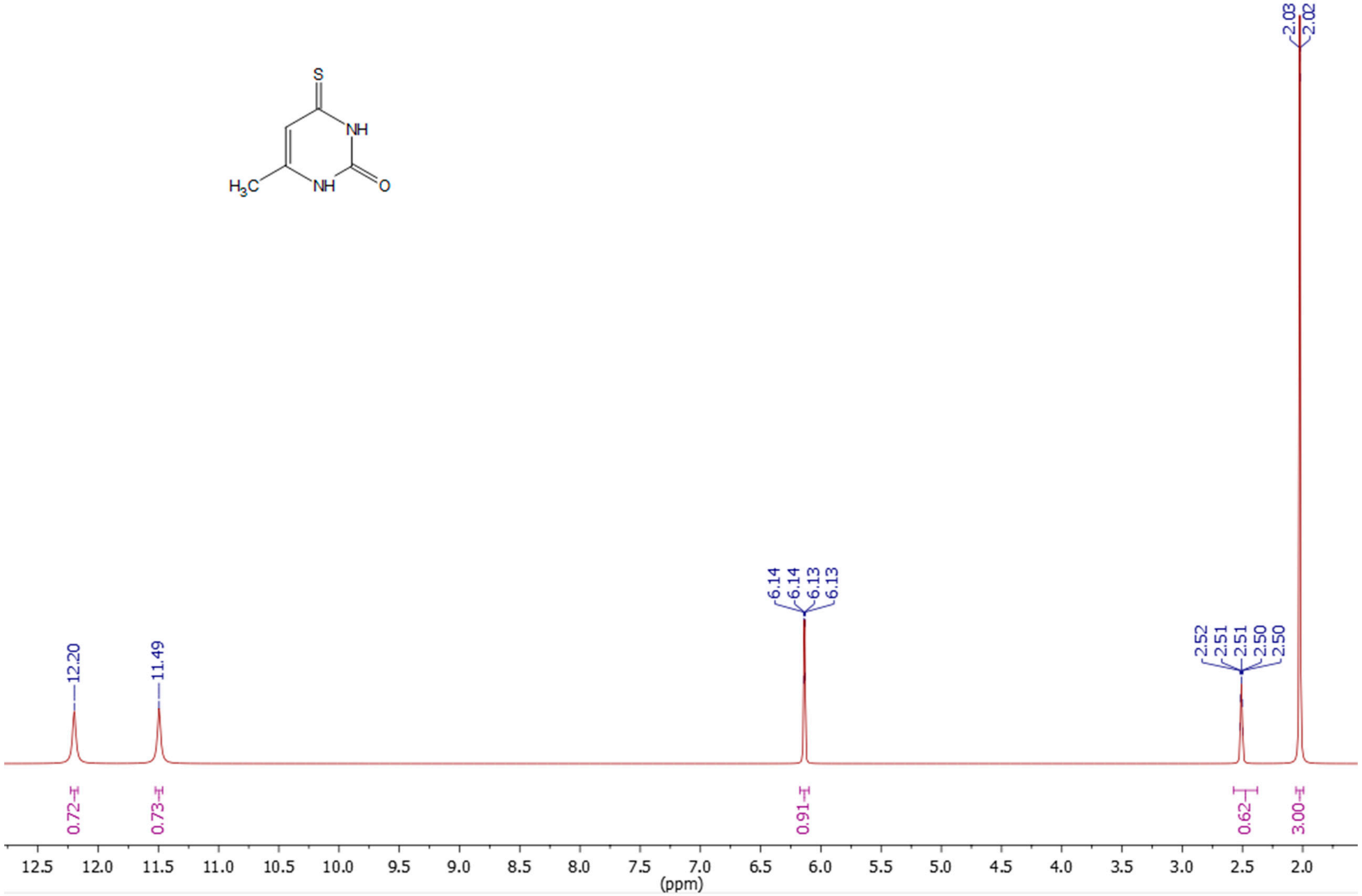

12) *N*[4]-Decyl-6-methylcytosine (12a)

[1]H

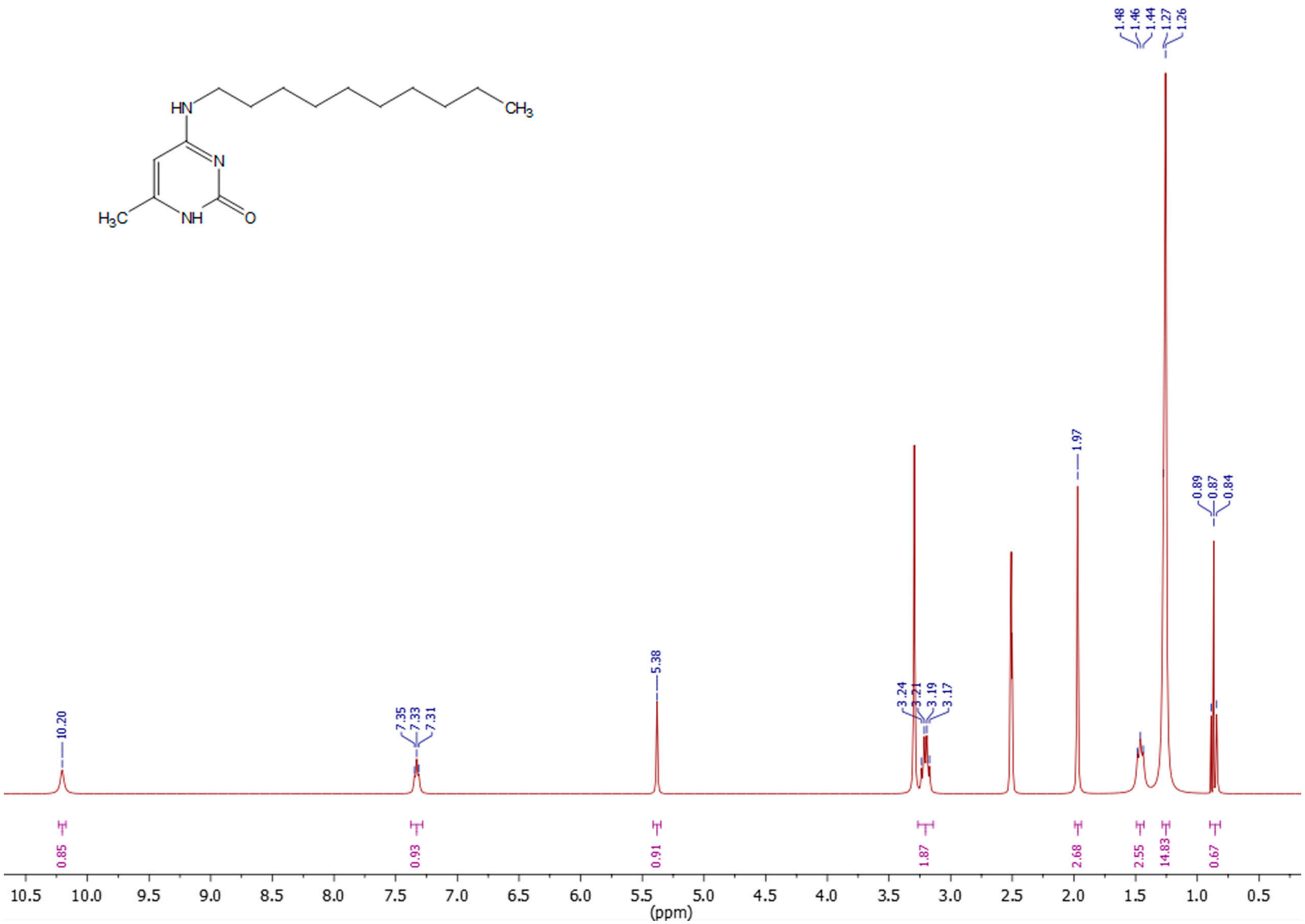

13) *N*[4]-Dodecyl-6-methylcytosine (12b)

^{1}H

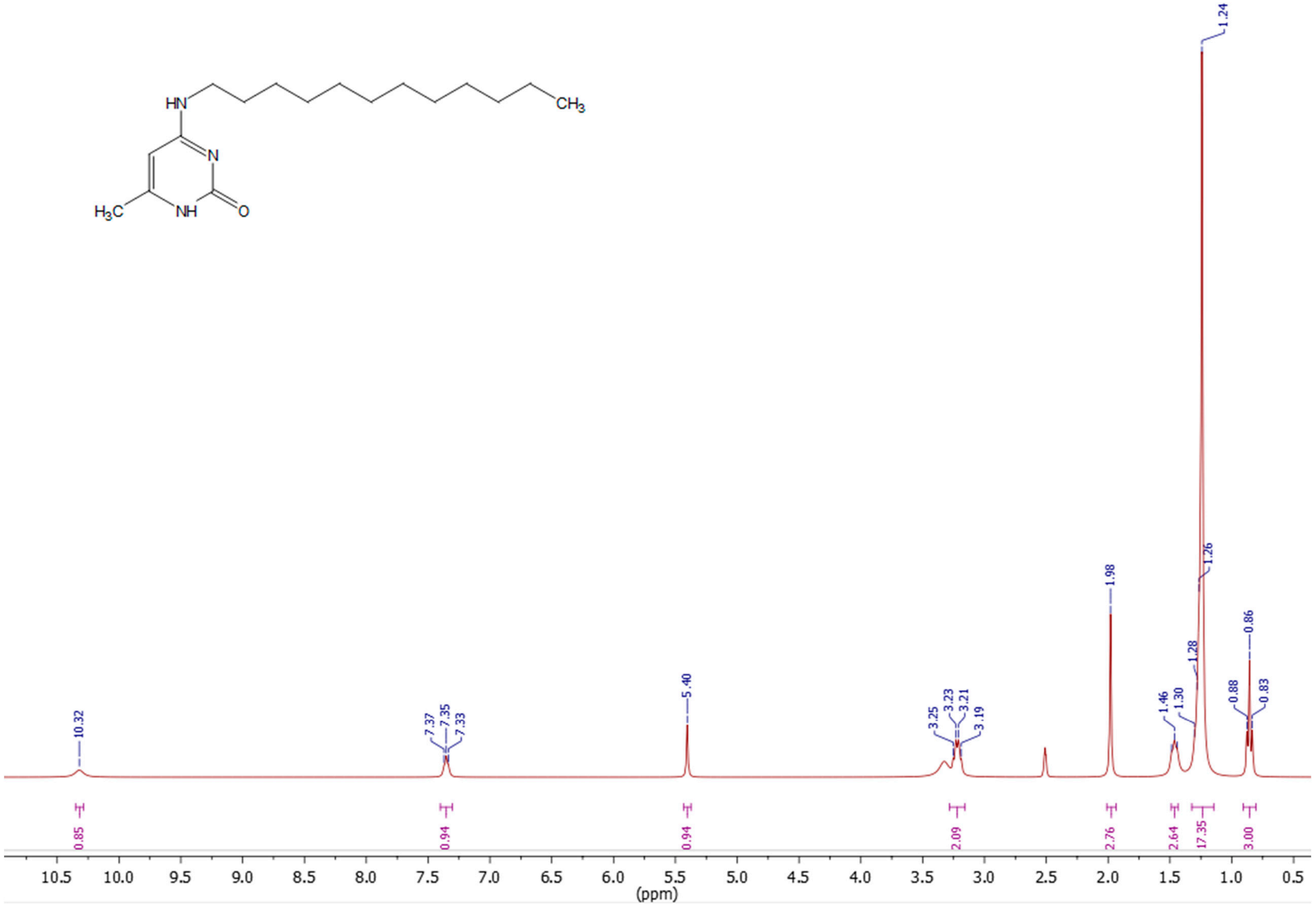

14) *N*[4]-Tetradecyl-6-methylcytosine (12c)

^{1}H

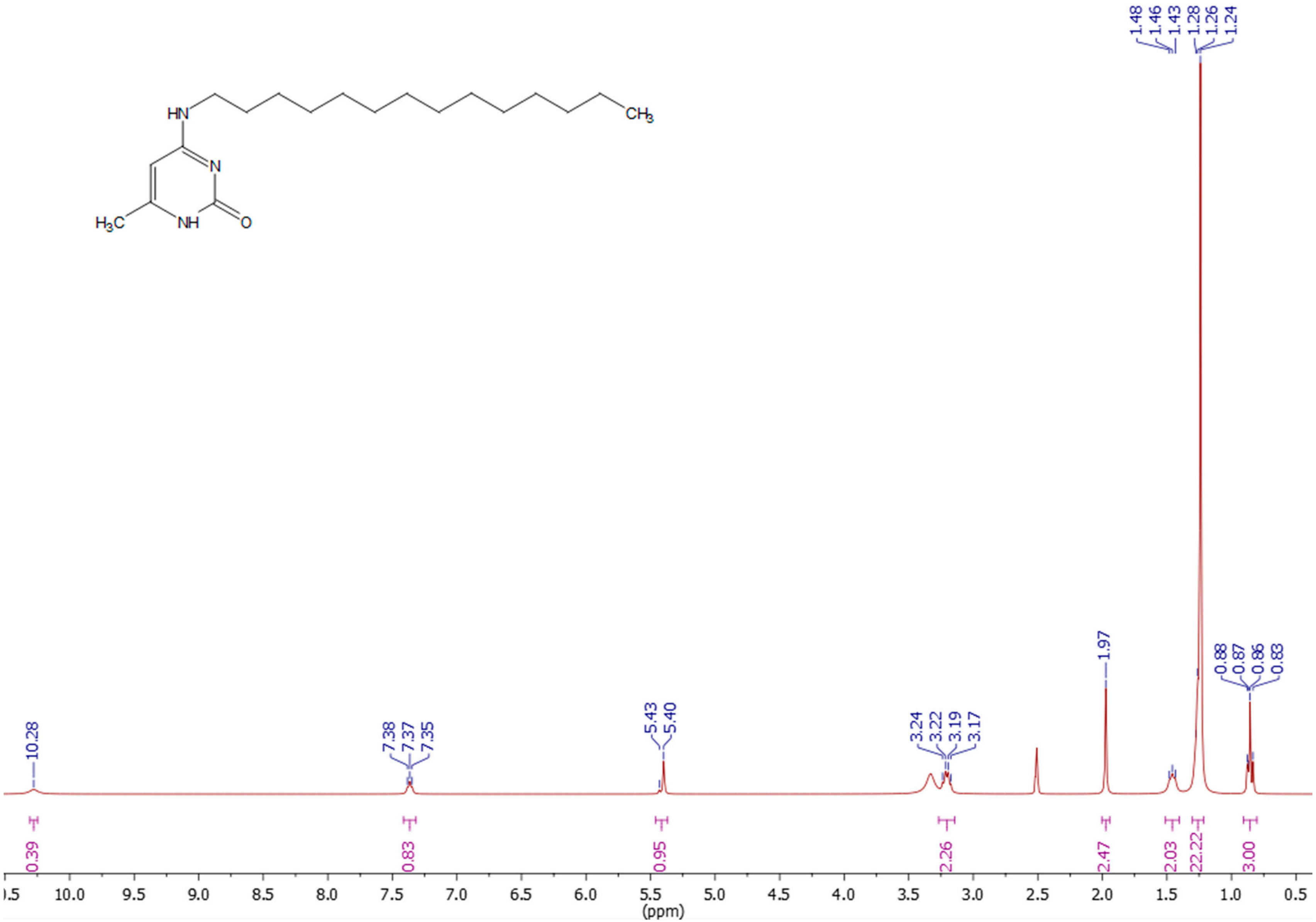

15) 2′,3′,5′-Tri-*O*-acetyl-*N*[4]-decyl-6-methylcytidine (13a)

[1]H

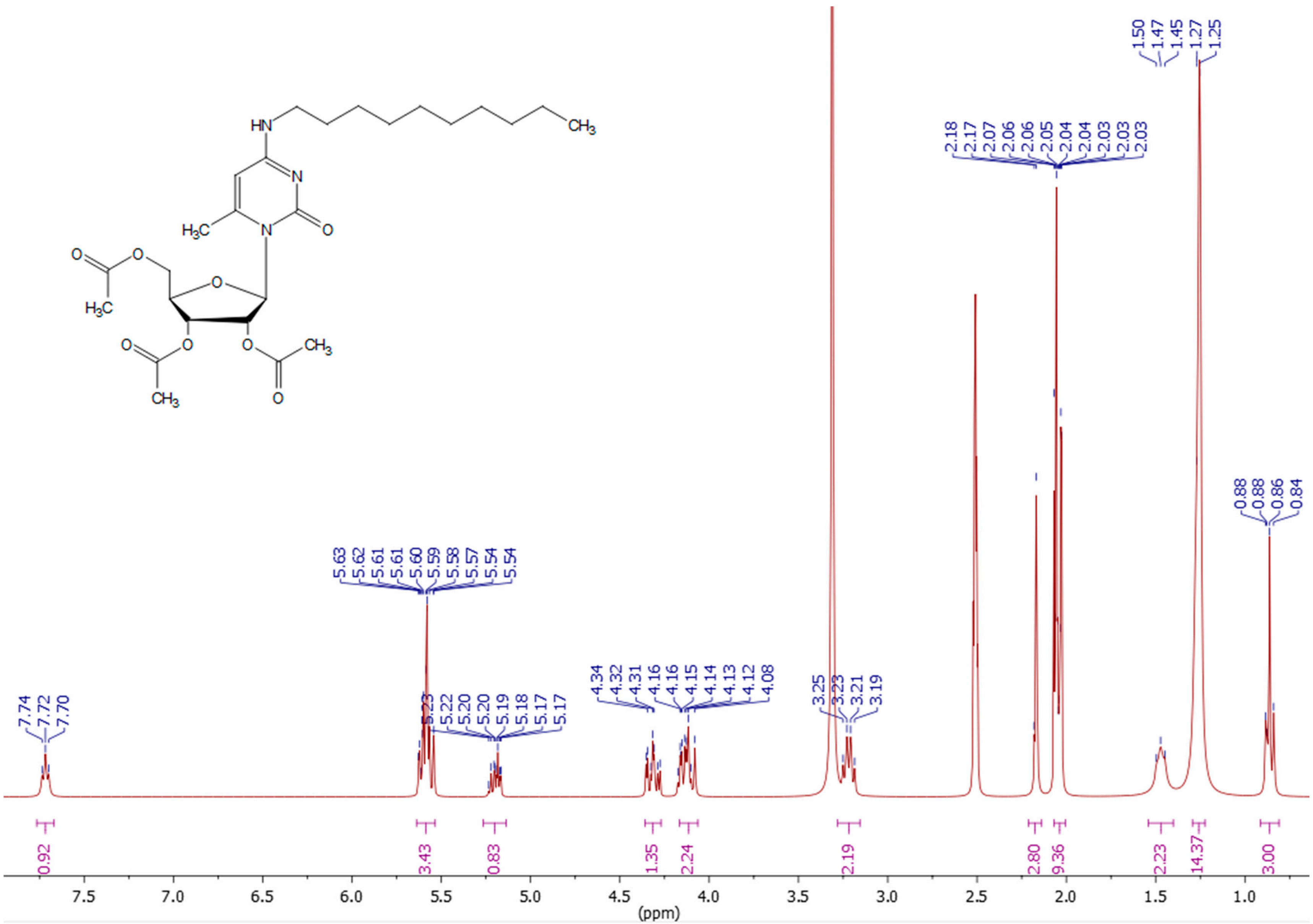

16) 2′,3′,5′-Tri-*O*-acetyl-*N*[4]-dodecyl-6-methylcytidine (13b)

^{1}H

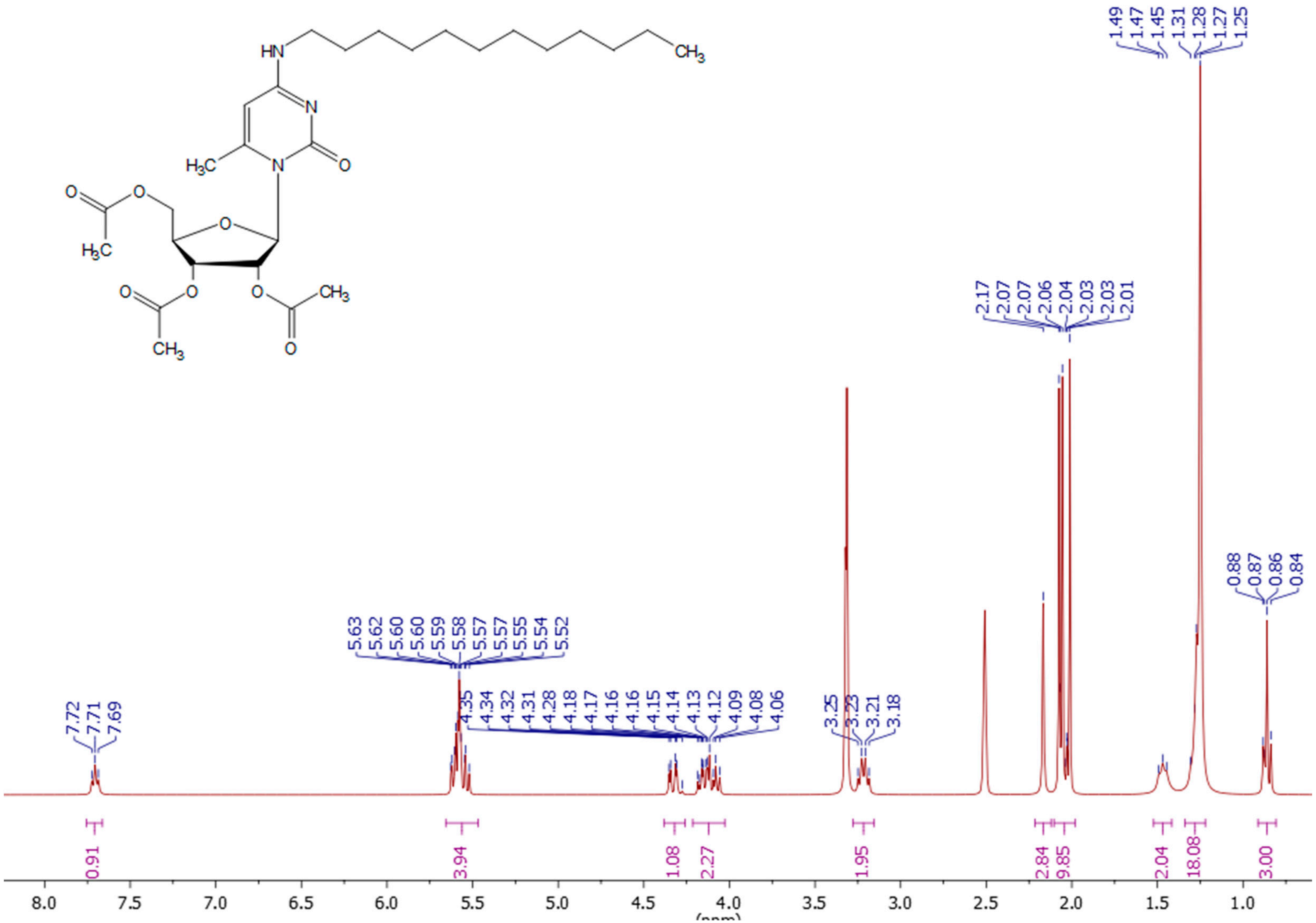

17) 2′,3′,5′-Tri-*O*-acetyl-*N*[4]-tetradecyl-6-methylcytidine (13c)

[1]H

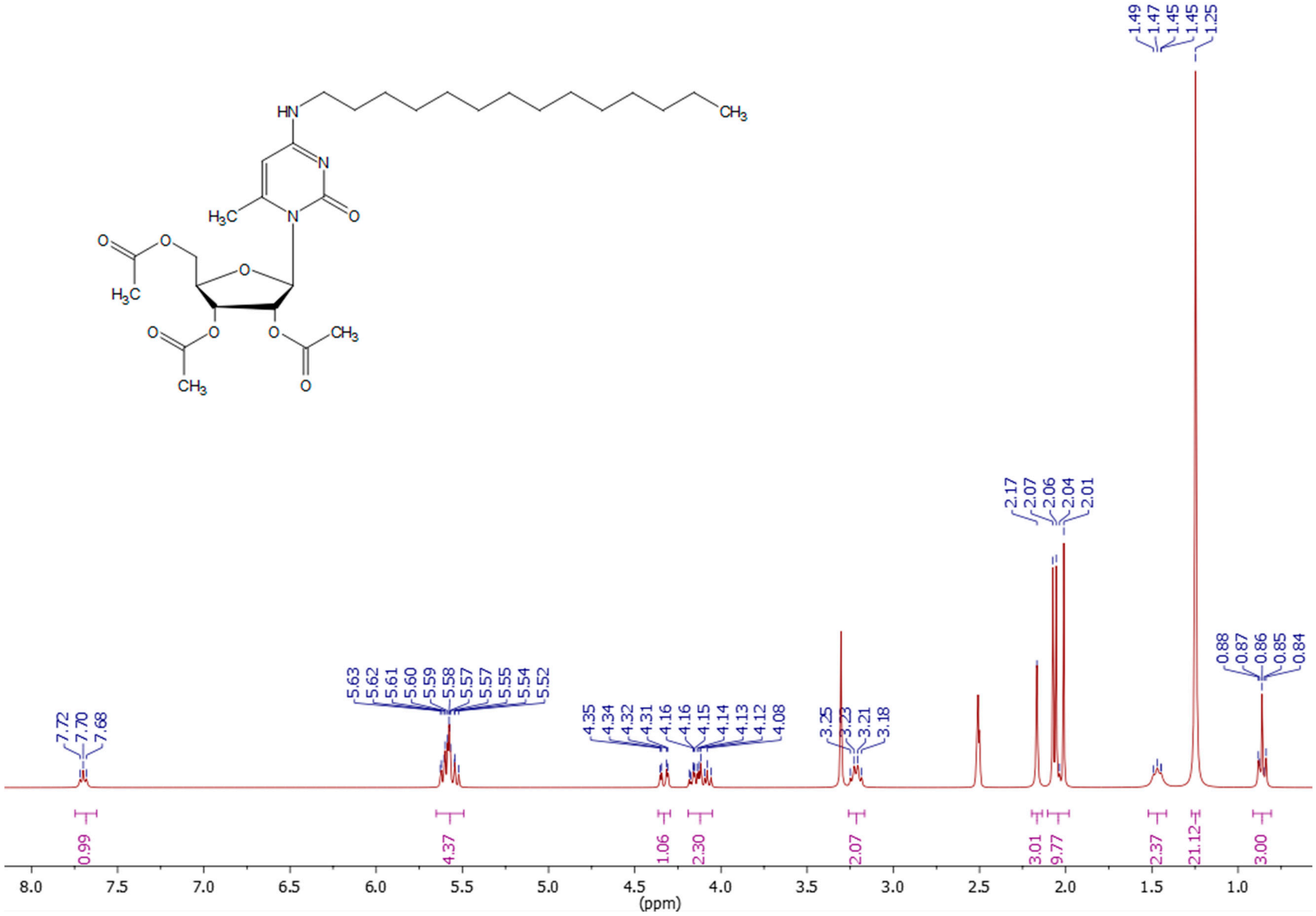

Figure S3. Mass spectra of new compounds

1) ***N*[4]-decyl-5-methylcytidine (2a)**

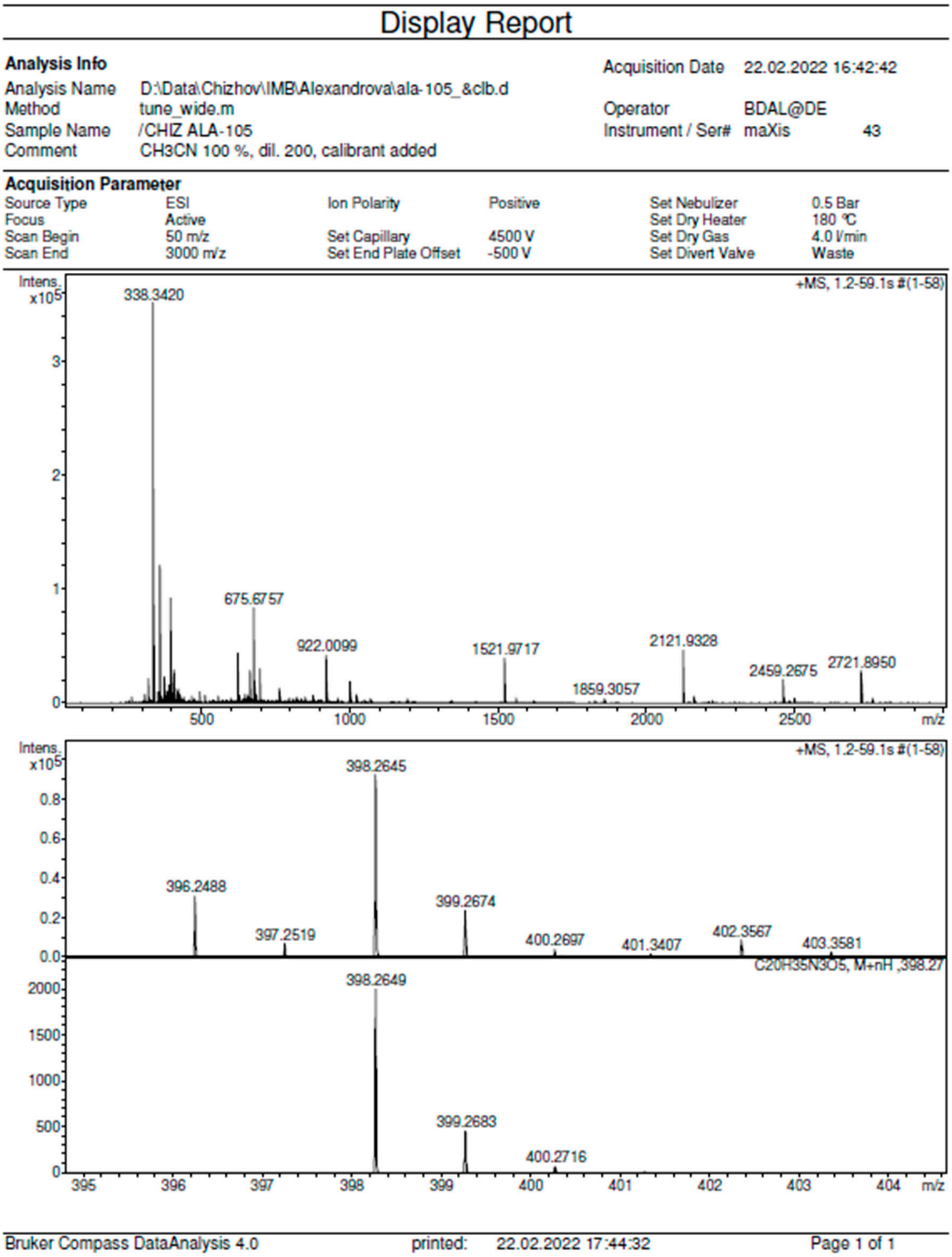

2) N^4-dodecyl-5-methylcytidine (2b)

Display Report

Analysis Info		Acquisition Date	22.02.2022 16:52:26
Analysis Name	D:\Data\Chizhov\IMB\Alexandrova\ala-106_&clb.d		
Method	tune_wide.m	Operator	BDAL@DE
Sample Name	/CHIZ ALA-106	Instrument / Ser#	maXis 43
Comment	CH3CN : H2O 50/50 %, dil. 400, calibrant added		

Acquisition Parameter

Source Type	ESI	Ion Polarity	Positive	Set Nebulizer	0.5 Bar
Focus	Active			Set Dry Heater	180 °C
Scan Begin	50 m/z	Set Capillary	4500 V	Set Dry Gas	4.0 l/min
Scan End	3000 m/z	Set End Plate Offset	-500 V	Set Divert Valve	Waste

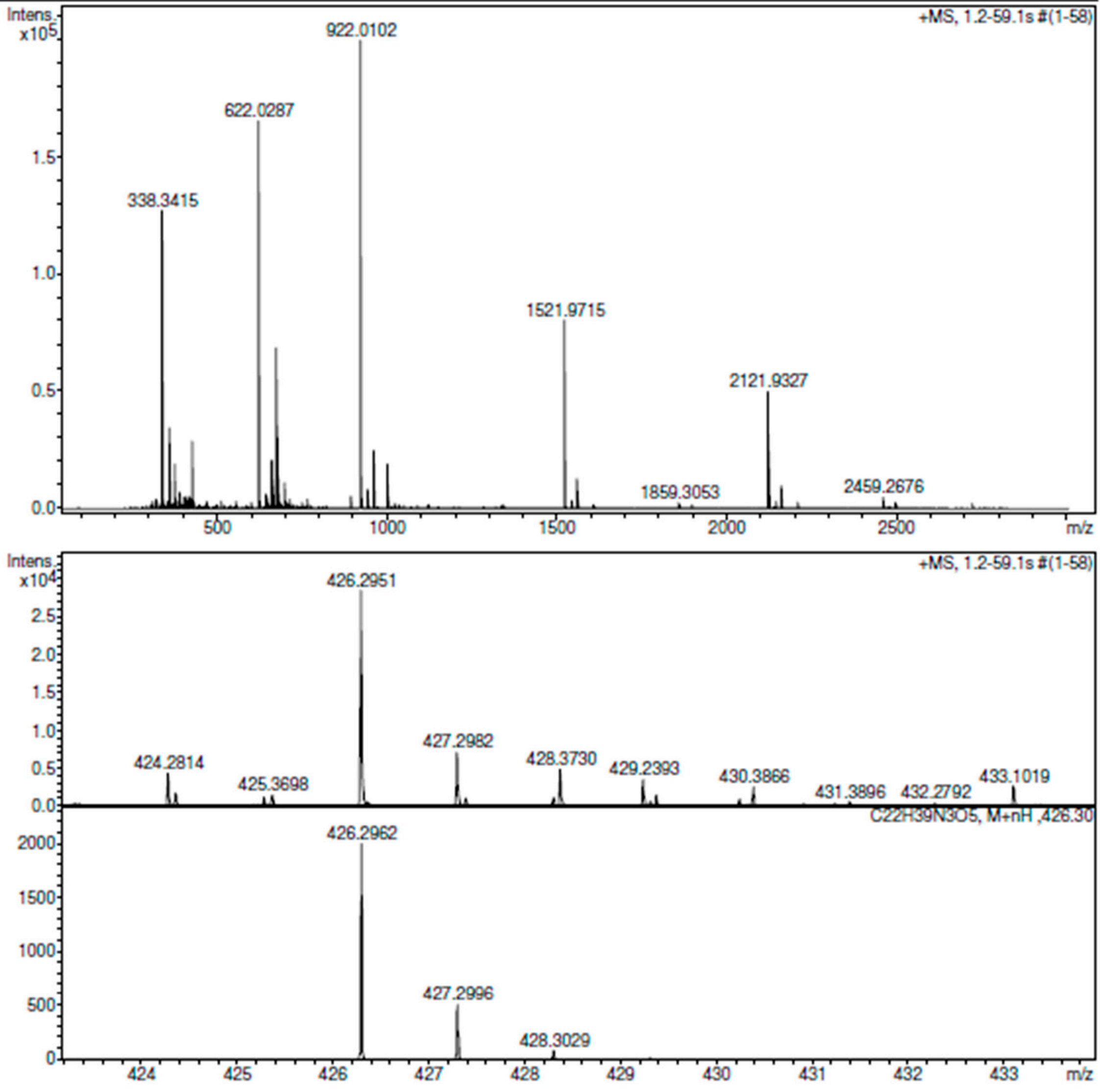

Bruker Compass DataAnalysis 4.0 printed: 22.02.2022 17:46:32 Page 1 of 1

3) *N*[4]-tetradecyl-5-methylcytidine (2c)

Display Report

Analysis Info

Analysis Name	D:\Data\Chizhov\IMB\Alexandrova\ala-107_&clb.d	Acquisition Date	22.02.2022 17:00:46
Method	tune_wide.m	Operator	BDAL@DE
Sample Name	/CHIZ ALA-107	Instrument / Ser#	maXis 43
Comment	CH3CN : H2O 50/50 %, dil. 400, calibrant added		

Acquisition Parameter

Source Type	ESI	Ion Polarity	Positive	Set Nebulizer	0.5 Bar
Focus	Active			Set Dry Heater	180 °C
Scan Begin	50 m/z	Set Capillary	4500 V	Set Dry Gas	4.0 l/min
Scan End	3000 m/z	Set End Plate Offset	-500 V	Set Divert Valve	Waste

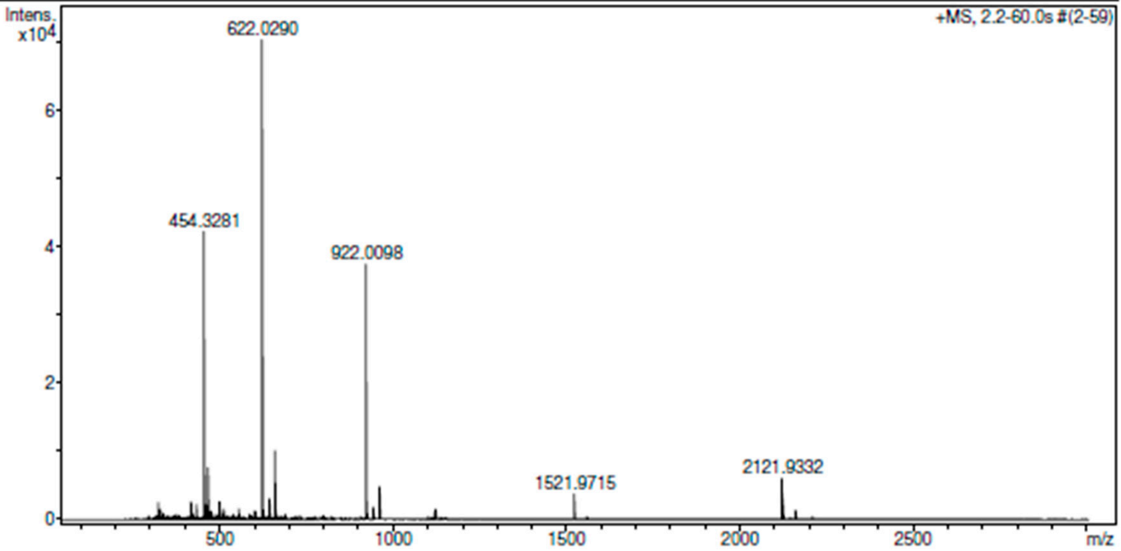

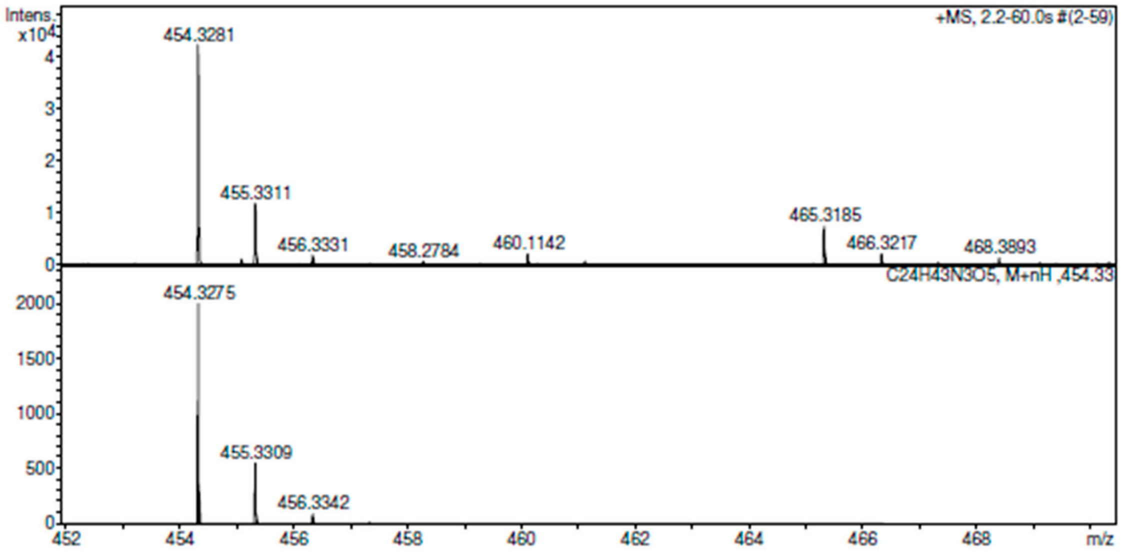

4) N^4-dodecylcytidine (2d)

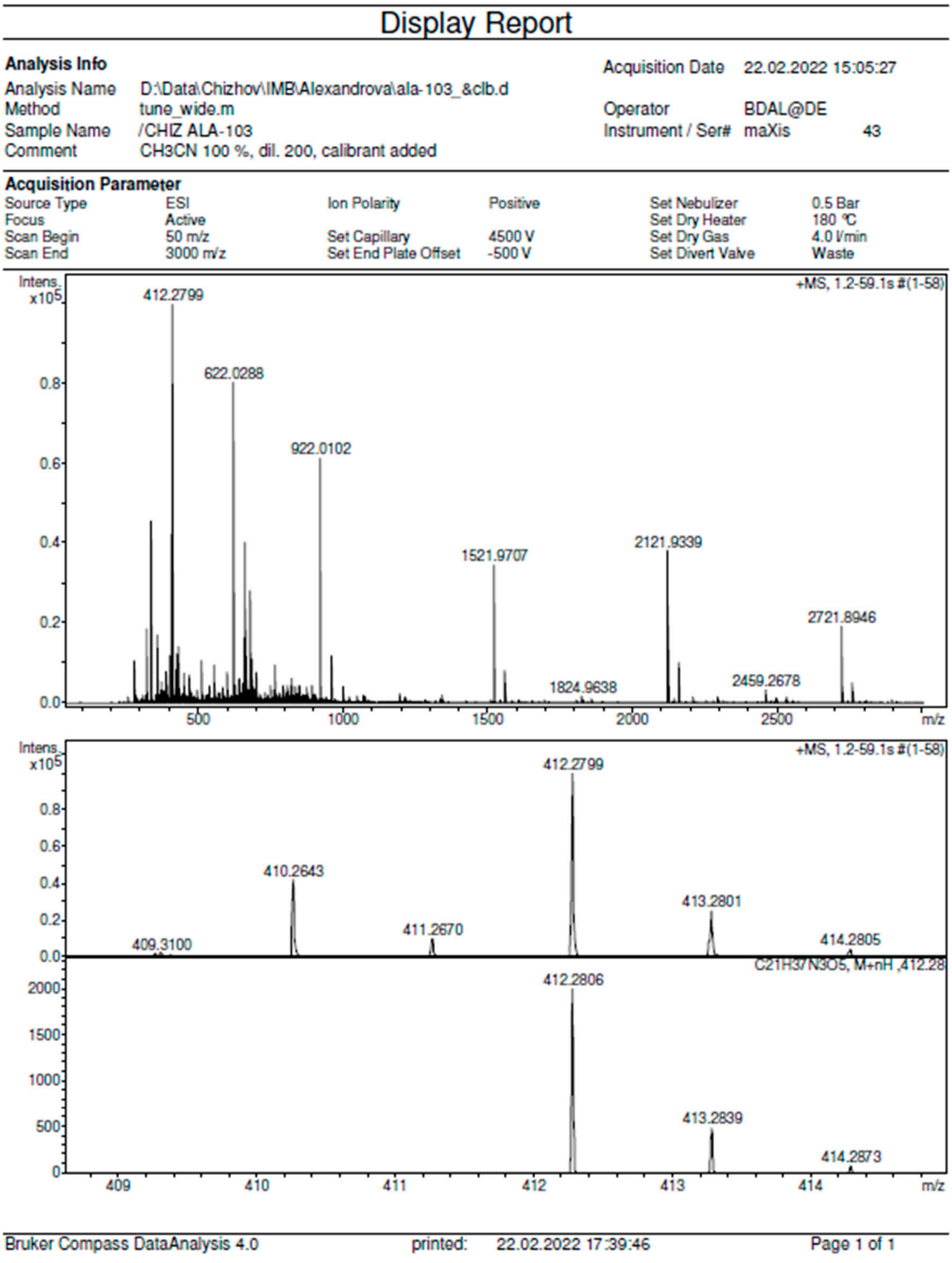

5) N^4-tetradecylcytidine (2e)

Display Report

Analysis Info

Analysis Name	D:\Data\Chizhov\IMB\Alexandrova\ala-104_&clb.d	Acquisition Date	22.02.2022 15:12:37
Method	tune_wide.m	Operator	BDAL@DE
Sample Name	/CHIZ ALA-104	Instrument / Ser#	maXis 43
Comment	CH3CN 100 %, dil. 2000, calibrant added		

Acquisition Parameter

Source Type	ESI	Ion Polarity	Positive	Set Nebulizer	0.5 Bar
Focus	Active			Set Dry Heater	180 °C
Scan Begin	50 m/z	Set Capillary	4500 V	Set Dry Gas	4.0 l/min
Scan End	3000 m/z	Set End Plate Offset	-500 V	Set Divert Valve	Waste

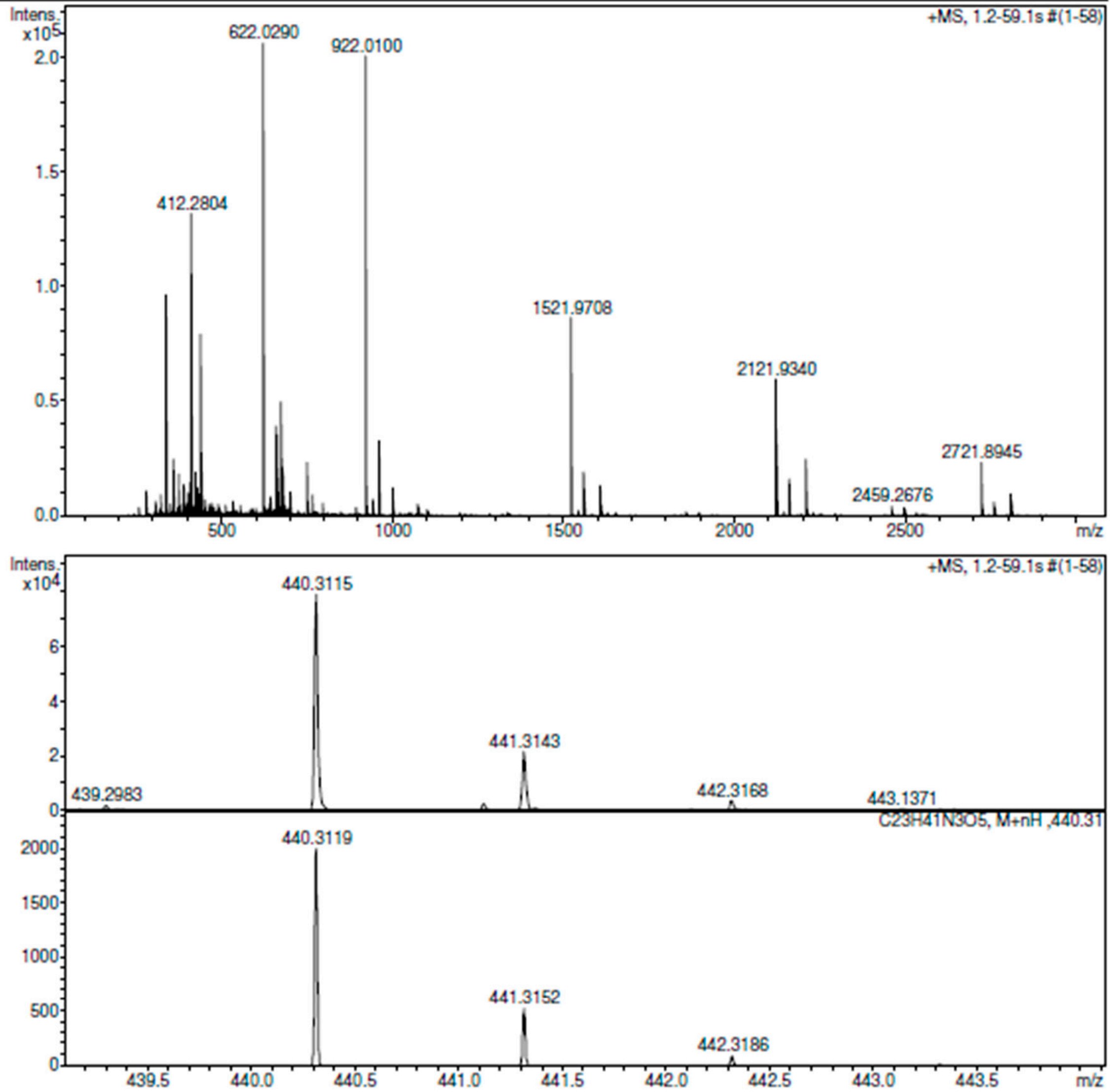

6) N^4-Decyl-6-methylcytidine (3a)

Display Report

Analysis Info

Analysis Name	D:\Data\Chizhov\IMB\Aleksandrova\Oskol'skii\osk-1_&clblow.d	Acquisition Date	19.01.2024 17:57:39
Method	tune_low.m	Operator	BDAL@DE
Sample Name	/CHIZ OSK-1	Instrument / Ser#	micrOTOF 10248
Comment	CH3CN:H2O 50/50 %, dil. 20, low conc. calibrant added		

Acquisition Parameter

Source Type	ESI	Ion Polarity	Positive	Set Nebulizer	0.4 Bar
Focus	Not active			Set Dry Heater	180 °C
Scan Begin	50 m/z	Set Capillary	4500 V	Set Dry Gas	4.0 l/min
Scan End	3000 m/z	Set End Plate Offset	-500 V	Set Divert Valve	Waste

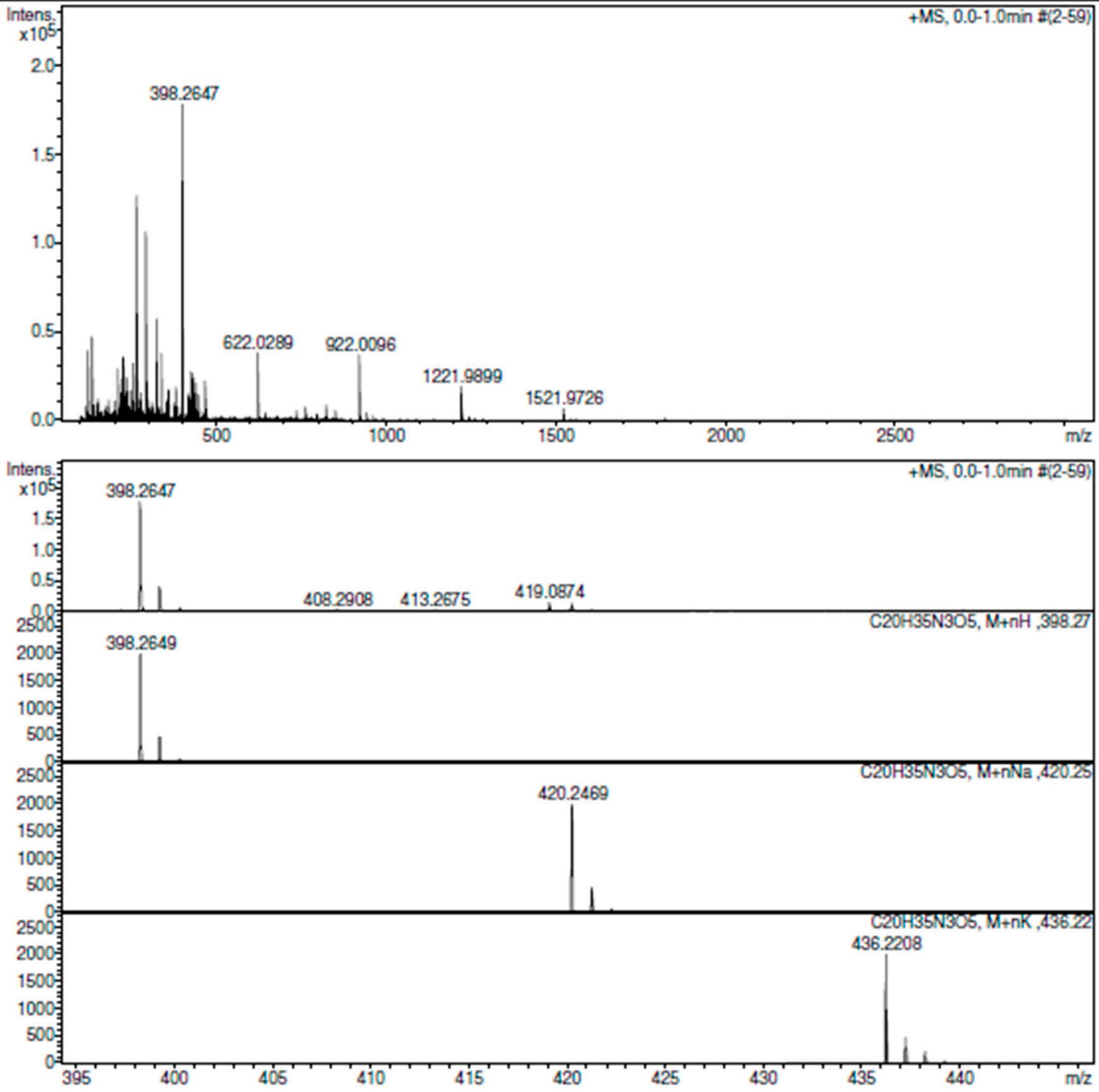

Bruker Compass DataAnalysis 4.0 printed: 19.01.2024 18:58:09 Page 1 of 1

7) N^4-Dodecyl-6-methylcytidine (3b)

Display Report

Analysis Info

Analysis Name	D:\Data\Chizhov\IMB\Aleksandrova\Oskol'skii\osk-2_&clblow.d	Acquisition Date	19.01.2024 18:02:22
Method	tune_low.m	Operator	BDAL@DE
Sample Name	/CHIZ OSK-2	Instrument / Ser#	micrOTOF 10248
Comment	CH3CN:H2O 50/50 %, dil. 20, low conc. calibrant added		

Acquisition Parameter

Source Type	ESI	Ion Polarity	Positive	Set Nebulizer	0.4 Bar
Focus	Not active			Set Dry Heater	180 °C
Scan Begin	50 m/z	Set Capillary	4500 V	Set Dry Gas	4.0 l/min
Scan End	3000 m/z	Set End Plate Offset	-500 V	Set Divert Valve	Waste

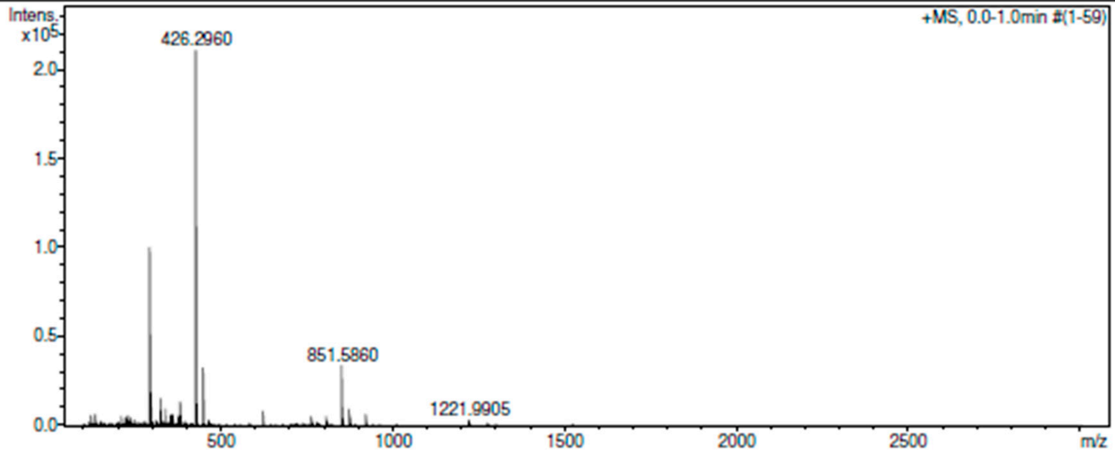

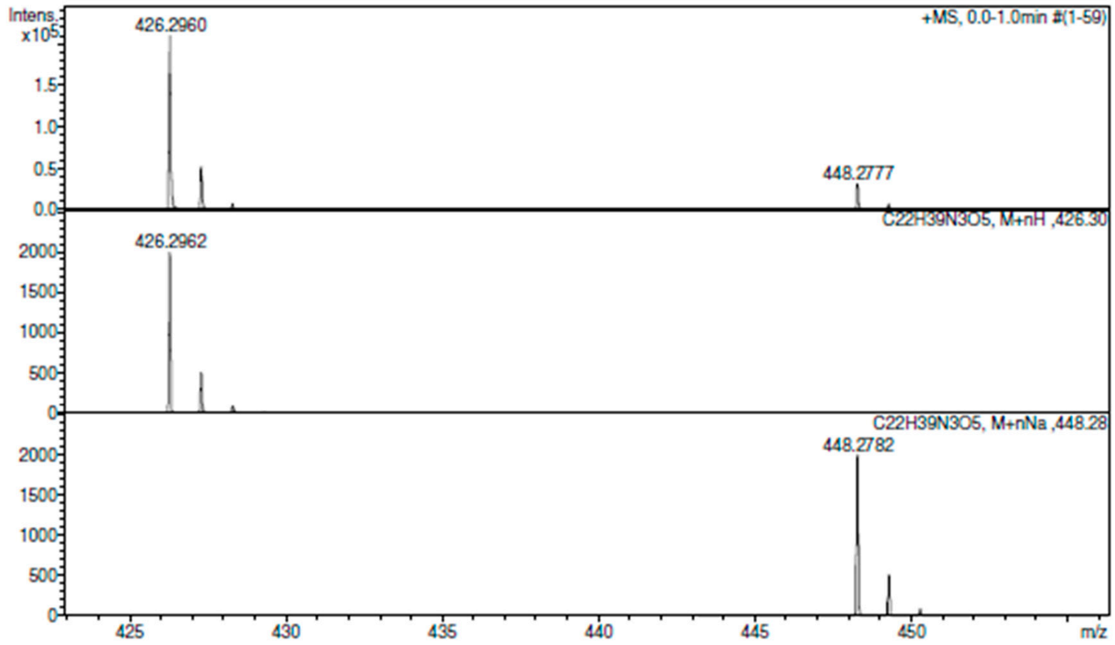

Bruker Compass DataAnalysis 4.0 printed: 19.01.2024 19:01:17 Page 1 of 1

8) *N*[4]-Tetradecyl-6-methylcytidine (3c)

Display Report

Analysis Info

Analysis Name	D:\Data\Chizhov\IMB\Aleksandrova\Oskol'skii\osk-3_&clblow.d	Acquisition Date	19.01.2024 18:09:39
Method	tune_low.m	Operator	BDAL@DE
Sample Name	/CHIZ OSK-3	Instrument / Ser#	micrOTOF 10248
Comment	CH3CN:H2O 50/50 %, dil. 200, low conc. calibrant added		

Acquisition Parameter

Source Type	ESI	Ion Polarity	Positive	Set Nebulizer	0.4 Bar
Focus	Not active			Set Dry Heater	180 °C
Scan Begin	50 m/z	Set Capillary	4500 V	Set Dry Gas	4.0 l/min
Scan End	3000 m/z	Set End Plate Offset	-500 V	Set Divert Valve	Waste

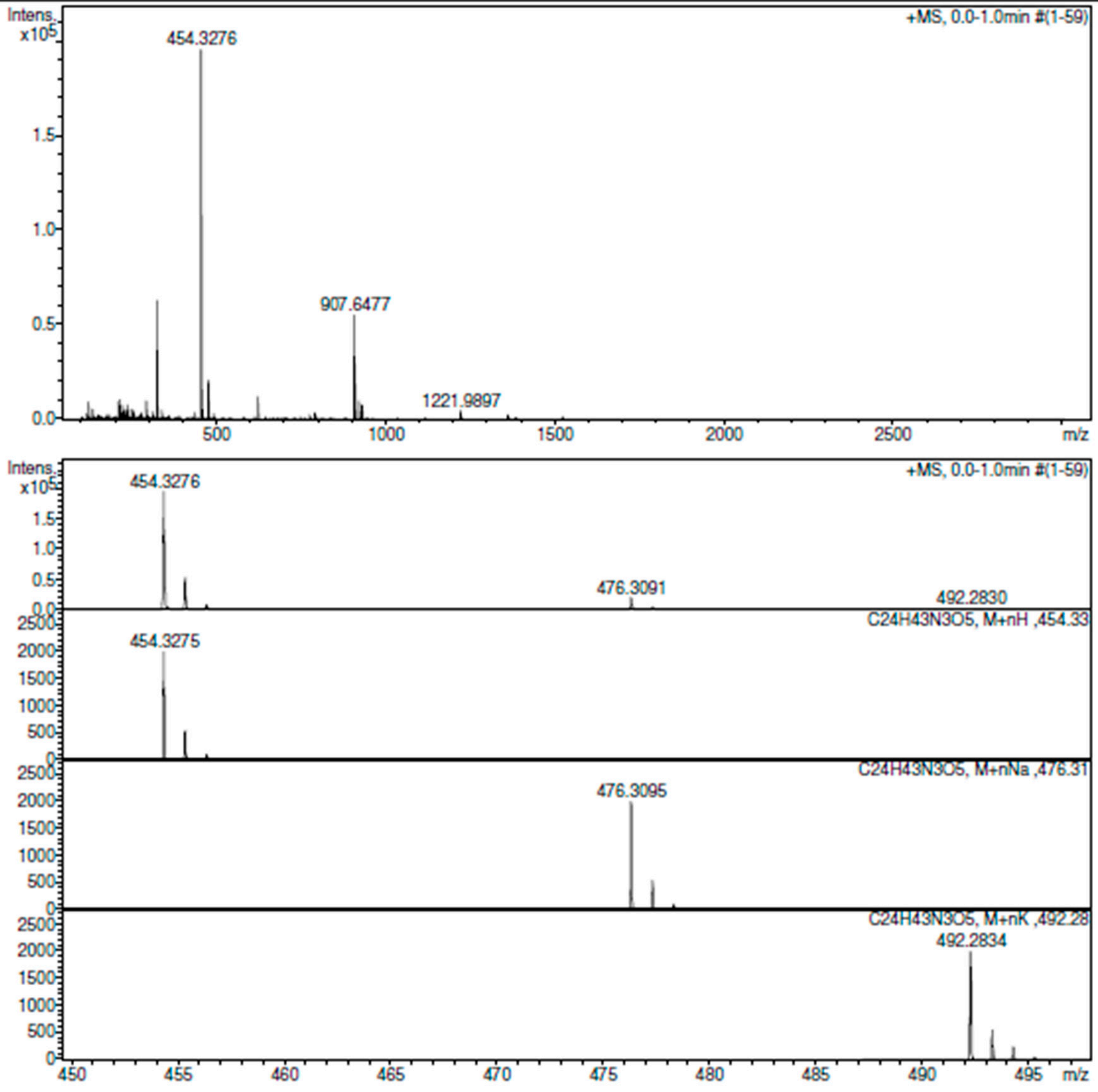

Bruker Compass DataAnalysis 4.0 printed: 19.01.2024 19:04:21 Page 1 of 1

9) N^4-Decyl-6-methylcytosine (12a)

Display Report

Analysis Info

Analysis Name	D:\Data\Chizhov\IMB\Aleksandrova\Oskol'skii\osk-4_&clblow.d	Acquisition Date	19.01.2024 18:19:07
Method	tune_low.m	Operator	BDAL@DE
Sample Name	/CHIZ OSK-4	Instrument / Ser#	micrOTOF 10248
Comment	CH3CN:H2O 50/50 %, dil.200, low conc. calibrant added		

Acquisition Parameter

Source Type	ESI	Ion Polarity	Positive	Set Nebulizer	0.4 Bar
Focus	Not active			Set Dry Heater	180 °C
Scan Begin	50 m/z	Set Capillary	4500 V	Set Dry Gas	4.0 l/min
Scan End	3000 m/z	Set End Plate Offset	-500 V	Set Divert Valve	Waste

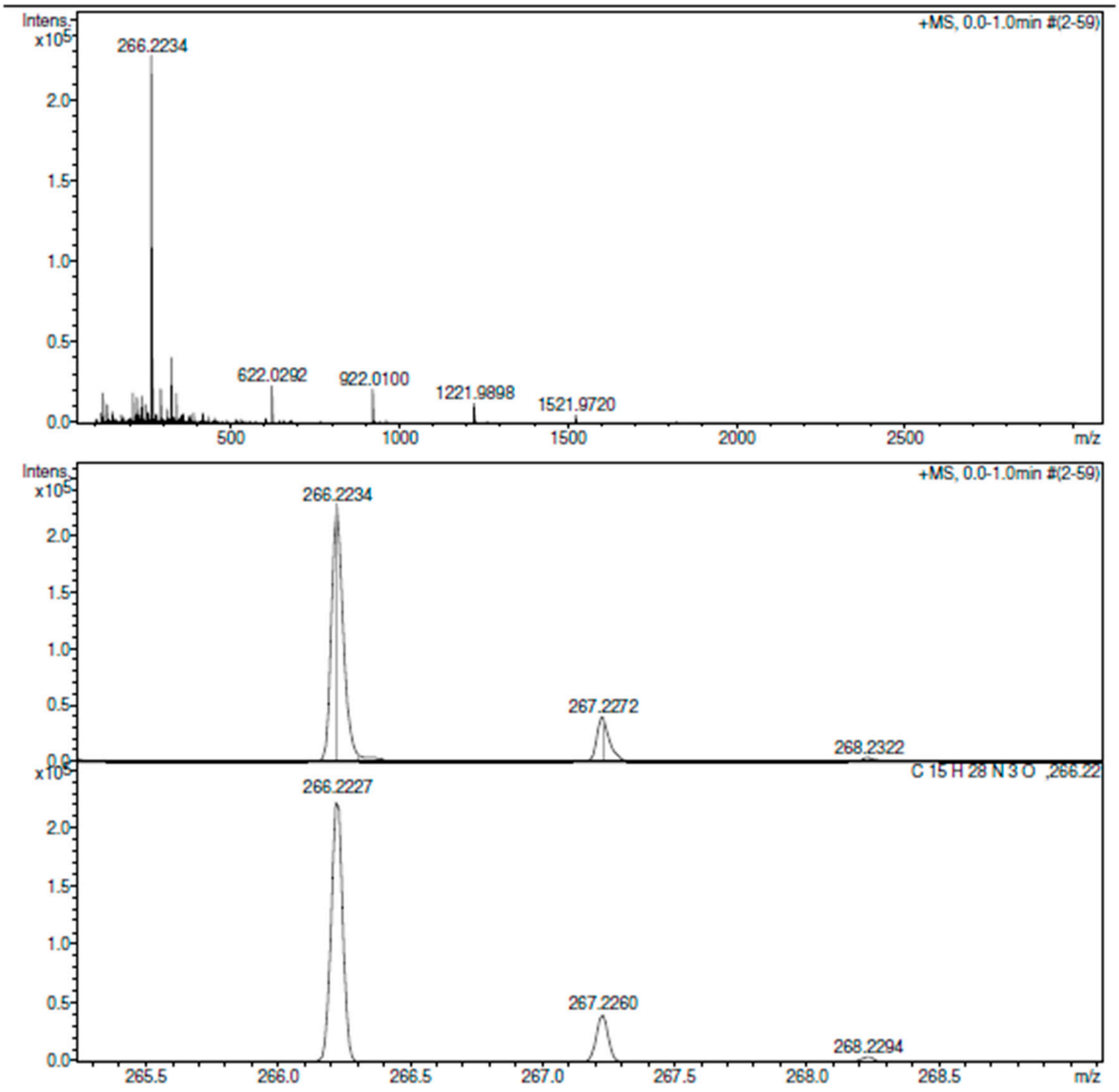

Bruker Compass DataAnalysis 4.0 printed: 19.01.2024 19:07:09 Page 1 of 1

10) *N*[4]-Dodecyl-6-methylcytosine (12b)

Display Report

Analysis Info

Analysis Name	D:\Data\Chizhov\IMB\Aleksandrova\Oskol'skii\osk-5_&clblow.d	Acquisition Date	19.01.2024 18:23:26
Method	tune_low.m	Operator	BDAL@DE
Sample Name	/CHIZ OSK-5	Instrument / Ser#	micrOTOF 10248
Comment	CH3CN:H2O 50/50 %, dil.200, low conc. calibrant added		

Acquisition Parameter

Source Type	ESI	Ion Polarity	Positive	Set Nebulizer	0.4 Bar
Focus	Not active			Set Dry Heater	180 °C
Scan Begin	50 m/z	Set Capillary	4500 V	Set Dry Gas	4.0 l/min
Scan End	3000 m/z	Set End Plate Offset	-500 V	Set Divert Valve	Waste

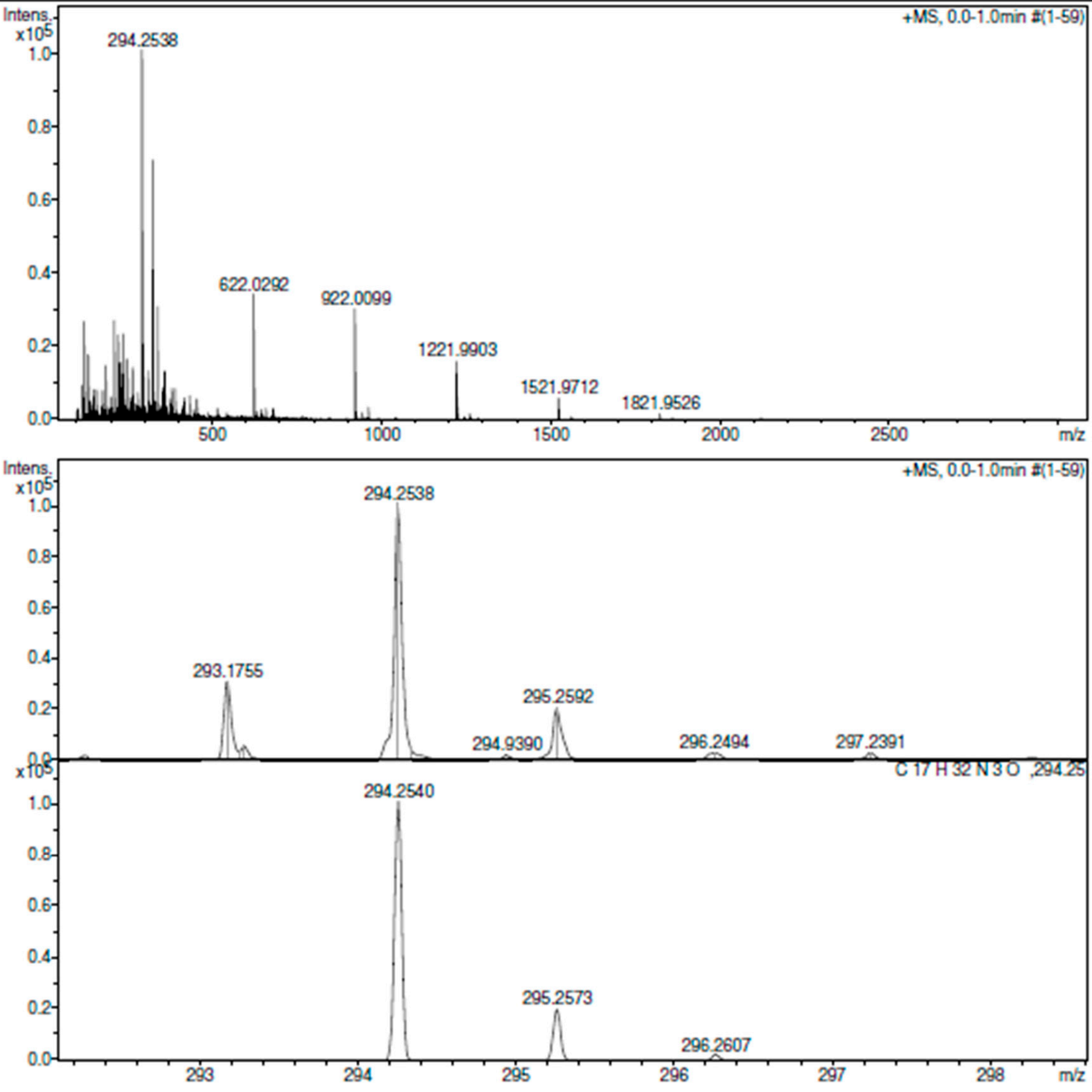

Bruker Compass DataAnalysis 4.0 printed: 19.01.2024 19:09:25 Page 1 of 1

11) *N*[4]-Tetradecyl-6-methylcytosine (12c)

Display Report

Analysis Info

Analysis Name	D:\Data\Chizhov\IMB\Aleksandrova\Oskol'skii\osk-6_low.d	Acquisition Date	19.01.2024 18:28:37
Method	tune_low.m	Operator	BDAL@DE
Sample Name	/CHIZ OSK-6	Instrument / Ser#	micrOTOF 10248
Comment	CH3CN:H2O 50/50 %, dil.200, no calibrant added		

Acquisition Parameter

Source Type	ESI	Ion Polarity	Positive	Set Nebulizer	0.4 Bar
Focus	Not active			Set Dry Heater	180 °C
Scan Begin	50 m/z	Set Capillary	4500 V	Set Dry Gas	4.0 l/min
Scan End	3000 m/z	Set End Plate Offset	-500 V	Set Divert Valve	Waste

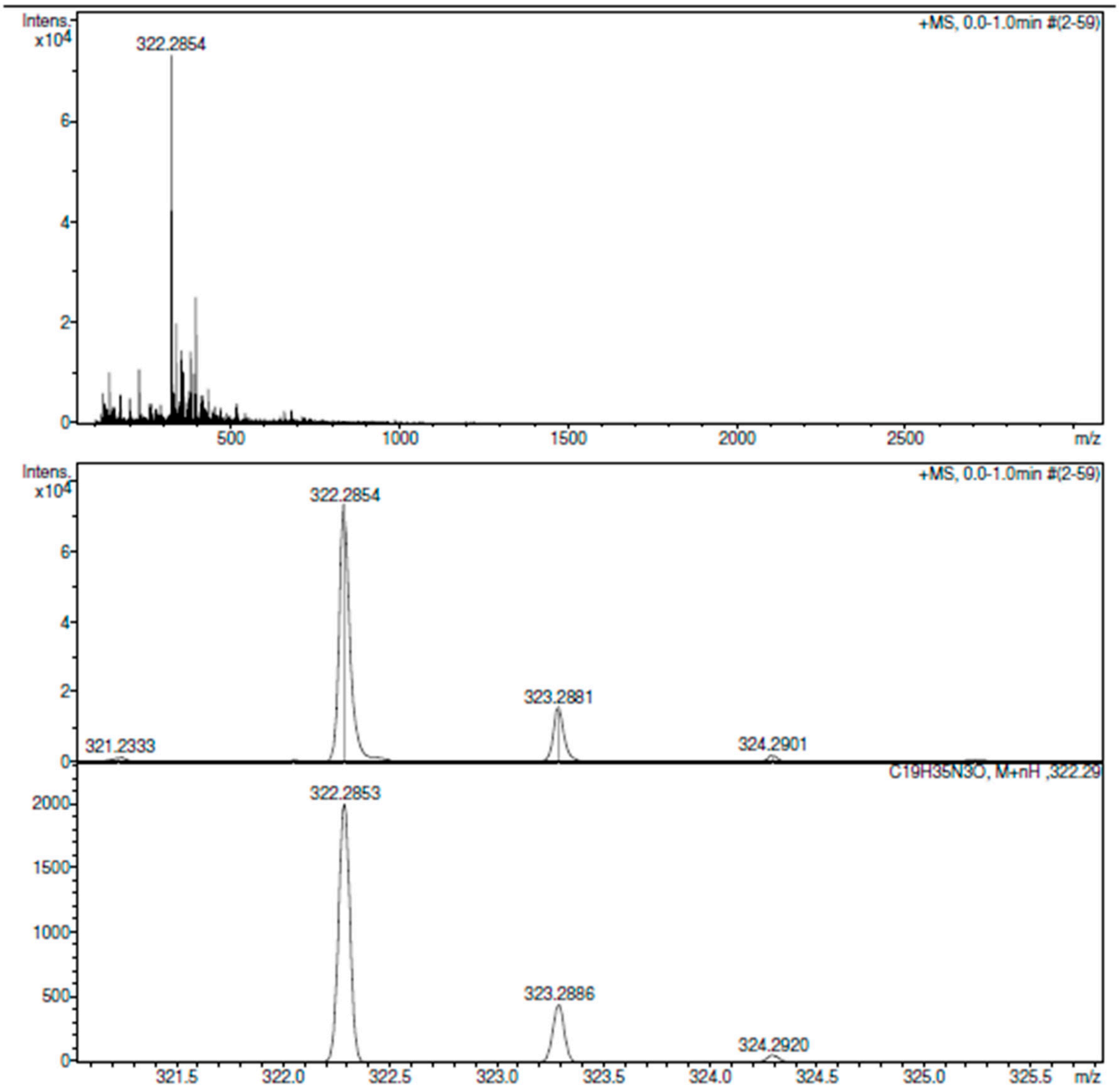

Bruker Compass DataAnalysis 4.0 printed: 19.01.2024 19:13:57 Page 1 of 1
